# Can Reactivity of Heart Rate Variability Be a Potential Biomarker and Monitoring Tool to Promote Healthy Aging? A Systematic Review With Meta-Analyses

**DOI:** 10.3389/fphys.2021.686129

**Published:** 2021-07-29

**Authors:** Patrick Manser, Melanie Thalmann, Manuela Adcock, Ruud H. Knols, Eling D. de Bruin

**Affiliations:** ^1^Department of Health Sciences and Technology, Movement Control and Learning–Institute of Human Movement Sciences and Sport, ETH Zurich, Zurich, Switzerland; ^2^Research and Education, Physiotherapy Occupational Therapy Research Center, University Hospital of Zurich, Zurich, Switzerland; ^3^Division of Physiotherapy, Department of Neurobiology, Care Sciences and Society, Karolinska Institutet, Stockholm, Sweden

**Keywords:** autonomic nervous system, biomarkers, cognition, exercise, healthy aging, heart rate variability, neurosciences, review (article)

## Abstract

**Background:** Monitoring phasic responses of heart rate variability (HRV) in terms of HRV reactivity [i. e., the absolute change from resting state to on-task (i.e., absolute values of HRV measured during exercise)] might provide useful insights into the individual psychophysiological responses of healthy middle-aged to older adults (HOA) to cognitive and physical exercises.

**Objectives:** To summarize the evidence of phasic HRV responses to cognitive and physical exercises, and to evaluate key moderating factors influencing these responses.

**Methods:** A systematic review with meta-analyses was performed. Publications up to May 2020 of the databases Medline (EBSCO), Embase, Cochrane Library, CINAHL, Psycinfo, Web of Science, Scopus, and Pedro were considered. Controlled clinical trials and observational studies measuring phasic HRV responses to cognitive and/or physical exercises in HOA (≥50 years) were included.

**Results:** The initial search identified 6,828 articles, of which 43 were included into the systematic review. Compared to resting state, vagally-mediated HRV indices were significantly reduced during all types of exercises [Hedge's g = −0.608, 95 % CI (−0.999 to −0.218), *p* = 0.002] indicating a significant parasympathetic withdrawal compared to rest. The key moderating variables of these responses identified included exercise intensity for physical exercises, and participant characteristics (i.e., level of cognitive functioning, physical fitness), task demands (i.e., task complexity and modality) and the individual responses to these cognitive challenges for cognitive exercises. In particular, higher task demands (task complexity and physical exercise intensity) were related to larger HRV reactivities. Better physical fitness and cognition were associated with lower HRV reactivities. Additionally, HRV reactivity appeared to be sensitive to training-induced cognitive and neural changes.

**Conclusion:** HRV reactivity seems to be a promising biomarker for monitoring internal training load and evaluating neurobiological effects of training interventions. Further research is warranted to evaluate the potential of HRV reactivity as a monitoring parameter to guide cognitive-motor training interventions and/or as a biomarker for cognitive impairment. This may facilitate the early detection of cognitive impairment as well as allow individualized training adaptations that, in turn, support the healthy aging process by optimizing individual exercise dose and progression of cognitive-motor training.

## Introduction

### Rationale

Maintaining good cognitive and physical functioning plays a fundamental role in healthy aging and well-being (Yam and Marsiske, 2013; Organization, 2015; Reuter-Lorenz et al., 2016; Zanjari et al., 2017). Nevertheless, the normal aging process is associated with structural and functional changes in the brain that are associated with a gradual decline in physical and cognitive abilities, possibly limiting functional abilities of daily life and quality of life (Harada et al., 2013; Bennett and Madden, 2014; Lockhart and DeCarli, 2014; Dumas, 2015; Murman, 2015; Organization, 2015; Cleeland et al., 2019; Salthouse, 2019). This functional decline exists on a continuum from healthy aging to pathological states like “mild cognitive impairment” or “dementia” (Petersen et al., 1997, 2014; Lindbergh et al., 2016; Sanford, 2017; Janelidze and Botchorishvili, 2018). In 2015, 46.8 million people were living with dementia (Prince et al., 2015). The prevalence for mild neurocognitive disorders (mNCD) is more than twice as high as for dementia and ranges between 3 and 54% depending on the clinical classification (Petersen et al., 2009, 2014, 2018; Hu et al., 2017; Janelidze and Botchorishvili, 2018; Parnetti et al., 2019). The globally growing life expectancy serves as a risk the factor for cognitive decline and is accordingly expected to boost the incidence and prevalence of neurocognitive disorders including dementia (Hu et al., 2017; Kontis et al., 2017; Sanford, 2017; Janelidze and Botchorishvili, 2018; Levine et al., 2018; Gillis et al., 2019; Parnetti et al., 2019). A physically or cognitively sedentary lifestyle is another highly prevalent risk factor associated with cognitive decline and increased risk for cognitive impairment (e.g., dementia) in the aging population (Verghese et al., 2006; Geda et al., 2010; Guthold et al., 2018; Licher et al., 2019). Consequently, the worldwide prevalence of dementia is expected to nearly double every 20 years (Lindbergh et al., 2016).

To counteract the expected cognitive decline in individuals at risk, an early detection and prevention of cognitive impairment is crucial (Morley et al., 2015). Adaptations in lifestyle can endorse a healthier aging process, improve the aging immune system and slow down cognitive decline (Daskalopoulou et al., 2017; Bosnes et al., 2019; Erickson et al., 2019; Nieman and Wentz, 2019). Recent investigations have shown that non-pharmacological interventions (e.g., changes in lifestyle like physical activity, cognitive stimulation, and/or reductions of vascular risk factors) are powerful protectors for brain atrophy and cognitive decline (Erickson et al., 2010; Sofi et al., 2011; Beydoun et al., 2014; Blondell et al., 2014; Carvalho et al., 2014; Beckett et al., 2015; Guure et al., 2017; Hill et al., 2017; Mewborn et al., 2017; Brasure et al., 2018; Butler et al., 2018; Gomes-Osman et al., 2018; Lee, 2018; Liang et al., 2018; Nguyen et al., 2019; Sanders et al., 2019; Cunningham et al., 2020). Especially simultaneous cognitive-motor training, often incorporated in exergames, seems to be effective to improve cognition in both HOA and older adults with mNCD (Ogawa et al., 2016; Howes et al., 2017; Levin et al., 2017; Stanmore et al., 2017; Tait et al., 2017; Joubert and Chainay, 2018; Northey et al., 2018; Wang et al., 2019; Wu et al., 2019; Biazus-Sehn et al., 2020; Chan et al., 2020; Chen et al., 2020; Mansor et al., 2020) while they are, at the same time, able to improve physical (i.e., gait, mobility, activities of daily living) and psychosocial (i.e., motivation, anxiety, well-being, and quality of life) aspects (Theill et al., 2013; Schoene et al., 2014; Levin et al., 2017; van Santen et al., 2018; Farhang et al., 2019; Yang et al., 2019; Swinnen et al., 2020; Zhao et al., 2020). Nevertheless, despite numerous investigations, it is currently difficult to draw reliable conclusions about the underlying mechanisms and effectiveness of exergames. This is mainly due to the large heterogeneities between studies and inconsistencies in reporting training components (Ogawa et al., 2016; Howes et al., 2017; Stanmore et al., 2017; Tait et al., 2017; van Santen et al., 2018; Sokolov et al., 2020; Swinnen et al., 2020; Zhao et al., 2020). Therefore, further investigations are needed “to establish the neurobiological mechanisms and effective components of exergames for cognition and apply this understanding in the development of evidence-based exergame interventions” (Stanmore et al., 2017).

In most training studies, exercise programs are developed and applied based on scientific literature, guidelines, and recommendations in combination with the practical experience of coaches. This approach requires that training programs are prescribed on a group level without information on how the individual has responded to previous training sessions. Such an approach may lead to success on a group level but might, at the same time, hide inter-individual differences in training response. The response of (older) individuals to different training modalities (e.g., types and intensities) depends on individual capabilities such as cognitive abilities, physical fitness and motor abilities, as well as demographic characteristics (e.g., age, gender, health status, and the socioemotional status including motivation, mood, or stress; Bouchard and Rankinen, 2001; Hautala et al., 2003; Herold et al., 2018). To overcome this limitation of a generalized exercise program offering, suggestions are made toward an individualized approach and application of adapted exercise prescription (Herold et al., 2019). As an example, Herold et al. (2019) recommend tailoring exercise loads (e.g., by manipulating exercise intensity) to the capabilities of each individual person. Optimally, the exercise parameters are operationalized and adapted to the individual using specific markers of the internal training load to provide comparable inter-individual exercise doses (Herold et al., 2019). This approach is believed allowing further insights into dose-response relationships and to result in more distinct training effects (Herold et al., 2019; Stojan and Voelcker-Rehage, 2019).

Exercise dose is defined as “a product of exercise variables (e.g., exercise intensity, exercise duration, type of exercise), training variables (e.g., frequency of training sessions), and the application of training principles and should be operationalized by using (a) specific marker(s) of internal load” (Wasfy and Baggish, 2016; Northey et al., 2018; Cabral et al., 2019; Erickson et al., 2019; Etnier et al., 2019; Falck et al., 2019; Herold et al., 2019; Ross et al., 2019). The internal training load, hence, is supposed to determine training outcomes (Impellizzeri et al., 2019). Thus, internal training load can be used and should be monitored as a primary parameter to maximize training benefits (Impellizzeri et al., 2019). It can be described as acute individual response [i.e., biomechanical, physiological, and/or psychological response(s)] to training characteristics (external load) and other influencing factors (e.g., climatic conditions, equipment, and ground condition; Impellizzeri et al., 2019).

An optimal measure of internal training load should reflect the “actual psychophysiological response that the body initiates to cope with the requirements elicited by the external load” (Impellizzeri et al., 2019). During cognitive-motor training (e.g., exergaming), the internal training load is mainly influenced by neurocognitive task demands and the physical exercise intensity (Netz, 2019). Comprehensive guidelines and checklists are available that provide classifications of training load regarding physical exercise intensity (e.g., percentage of individual maximal heart rate; Halson, 2014; Hoffman, 2014; Slade et al., 2016; American College of Sports Medicine et al., 2017; Herold et al., 2019). Therefore, objective monitoring of the relative physical intensity is readily applicable. However, for neurocognitive task demands—that serve as the driving mechanisms for task-specific neuroplasticity (Netz, 2019)—it is difficult to quantify the internal training load. So far, subjective measures such as ratings of perceived task difficulty or cognitive effort, objective performance measures (e.g., reaction time, accuracy, and error rate), and physiological measures including cardiac measures (e.g., heart rate, HRV, and blood pressure), brain activity (e.g., task-evoked electric brain potentials), and eye activity (e.g., pupillary dilation, blink rate) have been used to assess training load related to neurocognitive task demands (Paas et al., 2003; Brünken et al., 2010; Hughes et al., 2019).

According to the “cardiovascular reactivity hypothesis” (Obrist, 2012), real-time monitoring of cardiovascular responses to physical or cognitive stressors provides useful insights into individual psychophysiological response patterns. Effort-related cardiovascular reactivity has been reported to be related to cognitive (i.e., executive functioning) as well as physical (i.e., aerobic fitness, exercise performance) capabilities (D'Agosto et al., 2014; Silvestrini, 2017). Therefore, monitoring cardiovascular reactivity could be useful to evaluate training adaptations and may additionally be predictive of certain health conditions (Treiber et al., 2003; Lovallo, 2005; Borresen and Lambert, 2008; Phillips, 2011a,b; D'Agosto et al., 2014; Schiweck et al., 2019). In particular, quantifying beat-to-beat variation of the duration between heart beats (i.e., R-R-Interval), referred to as HRV, has gained considerable interest in diverse fields (Thayer, 2009). HRV reflects cardiac autonomic activity (i.e., parasympathetic modulation), which indicates the capability of the autonomic nervous system to respond flexibly to external stimuli and is sensitive to psychophysiological stressors (Rajendra Acharya et al., 2006; Singh et al., 2018a; Forte et al., 2019; Giannakakis et al., 2019; Hillmert et al., 2020).

Decreases in parasympathetic activity (i.e., lower vagally-mediated HRV) at rest were reported to be related to worse performance in cognitive exercises, especially in the domain of executive functioning (Forte et al., 2019). This observation is in line with the predictions of the “neurovisceral integration” model (Thayer and Lane, 2000) and its advancements (Thayer, 2009; Smith et al., 2017) suggesting that HRV is able to index the functional integrity of the central autonomic network (CAN) that regulates physiological, emotional and cognitive responses to environmental challenges (Thayer, 2009). The CAN consists of cortical-subcortical pathways including the prefrontal cortex (PFC), the anterior cingulate cortex (ACC), the insula, the hypothalamus, and the brainstem. The CAN mediates the internal regulation system that innervates the preganglionic sympathetic and parasympathetic (vagal) neurons, which control the heart rhythm *via* the sinoatrial node (Benarroch, 1993; Thayer, 2009; Gordan et al., 2015). In particular, the prefrontal cortex exerts a top-down inhibitory regulation of the limbic system, which, in turn, suppresses the activity of the parasympathetic input to the heart (Thayer, 2009). As a result, higher activities of prefrontal brain structures increase tonic HRV, whereas hypo-activation reduces HRV (Thayer and Sternberg, 2006; Thayer et al., 2012; Park and Thayer, 2014). A predominantly vagal control of the heart permits quick and flexible responses to environmental demands and promotes effective executive performance (Thayer and Lane, 2000; Thayer, 2009; Thayer et al., 2012; Smith et al., 2017). Taken together, higher resting HRV has been related to better cognitive performance whereas lower resting HRV has been associated with cognitive impairment, and was even considered as an early biomarker of cognitive deteriorations (Ranchet et al., 2017; Forte et al., 2019). However, the neurovisceral integration theory primarily focuses on tonic cardiac vagal control (Thayer and Lane, 2000; Thayer, 2009; Smith et al., 2017) while markers for internal training load necessitate measurement during exercise (Impellizzeri et al., 2019). Moreover, according to the “vagal tank theory” (Laborde et al., 2018), it is argued, that different levels of adaptability of cardiac vagal control should be considered (i.e., resting, reactivity, and recovery). In particular, considering the cardiac vagal reactivity to cognitive or physical exercises is important to understand the individual's adaptability to a specific situation (Laborde et al., 2018). In fact, recent systematic reviews have concluded, that phasic HRV responses are sensitive to task demands (e.g., difficulty, complexity, and duration) related to cognitive and mental effort in older adults with and without cognitive impairment (Castaldo et al., 2015; Ranchet et al., 2017; Kim et al., 2018; Charles and Nixon, 2019; Hughes et al., 2019; Tao et al., 2019). Furthermore, measures of phasic HRV responses are suitable to distinguish between different intensities and durations of physical exercises (e.g., cardiorespiratory; Dong, 2016; Michael et al., 2017; Gronwald and Hoos, 2020).

Taken together, phasic HRV responses seem to hold promise as a biomarker to monitor internal training load of cognitive-motor training. This would enable individualized training adaptations that, in turn, would allow the application of the optimal individual exercise dose and progression. However, to gain a better understanding of the possible applications of phasic HRV responses (in terms of HRV reactivity (i.e., the absolute change from resting state to on-task (i.e., absolute values of HRV measured during exercise) HRV), and to evaluate whether HRV reactivity indeed could be used as a proxy measure for internal training load, it is important to establish a comprehensive understanding of moderating variables on HRV reactivity in HOA.

### Objectives

The aim of this systematic review and meta-analysis was: (a) to summarize relevant literature monitoring phasic HRV responses to (1) cognitive exercises, (2) physical exercises, and (3) simultaneous cognitive-motor training in HOA, and; (b) to evaluate key moderating parameters influencing phasic HRV responses during these exercises.

#### PICOS-Scheme

To achieve the purpose of this systematic review a PICO research question “In healthy middle-aged to older human adults (P), how does physical, cognitive and cognitive-motor training (I) compared to rest (C) influence phasic HRV responses (O)?” was formulated.

**P**articipants: Healthy middle-aged to older human adults (mean age ≥50 years).
**I**nterventions: (1) Cognitive exercises [i.e., cognitive tasks requiring cognitive processes (e.g., attentional, executive, memory or visuo-spatial functions)], (2) physical exercises [e.g., cardiorespiratory exercise, resistance exercises, or neuromotor exercise training as defined by the American College of SportsMedicine (ACSM) (Garber et al., 2011)], and/or (3) simultaneous cognitive-motor training [as defined by Herold et al. (2018) (Herold et al., 2018)].
**C**omparison: Resting state HRV.
**O**utcomes: Phasic HRV responses [in terms of HRV reactivity i.e., the absolute change from resting state to on-task (i.e., absolute values of HRV measured during exercise) HRV].
**S**tudy Type: Controlled clinical trials (e.g., randomized controlled trials, non-randomized controlled trials), observational studies (e.g., cohort studies, cross-sectional studies, and case-control studies).

#### Research Questions

In healthy middle aged and older human adults (P), how does physical, cognitive and cognitive-motor training (I) compared to rest (C) influence phasic HRV responses (O)?

## Materials and Methods

This systematic review with meta-analysis was conducted in accordance with the established guidelines from the “Preferred Reporting Items for Systematic Reviews and Meta-Analyses: The PRISMA Statement” (Liberati et al., 2009; Moher et al., 2009; Page et al., 2020).

### Protocol and Registration

A protocol (not registered) for this systematic review with meta-analysis (Supplementary File A) was developed in accordance with the established guidelines from the “Preferred Reporting Items for Systematic Review and Meta-Analysis Protocols (PRISMA-P) 2015 Statement” (Moher et al., 2015).

### Eligibility Criteria

Controlled clinical trials and observational studies assessing phasic HRV responses to (1) cognitive exercises (2) physical exercises, and (3) simultaneous cognitive-motor training in HOA (i.e., mean age ≥50 years) were considered for this systematic review.

Studies were considered eligible if they fulfilled the following criteria: (1) monitoring of phasic HRV responses to (1) cognitive exercises [i.e., cognitive tasks requiring cognitive processes (e.g., attentional, executive, memory, or visuo-spatial functions)], (2) physical exercises [e.g., cardiorespiratory exercise, resistance exercises, or neuromotor exercise training as defined by the American College of Sports Medicine (ACSM) (Garber et al., 2011)], and/or (3) simultaneous cognitive-motor training [as defined by Herold et al. (2018)] (2) in HOA (i.e., mean age ≥50 years); (3) by means of validated devices based on electrocardiography (ECG), photoplethysmography (PPG), or pulsoxymetry; (4) meeting the standards of HRV measurement (Electrophysiology TFotESoCtNASoP, 1996; Shaffer and Ginsberg, 2017). Studies were excluded in case: (1) full text was not accessible (i.e., access was not provided by the author within a 30-day response window) and/or not written in English; (2) published before 1996; (3) sources were review articles, meta-analyses, preliminary reports, dissertations, conference abstracts, or posters, or (4) no additional resting-state measurement of HRV (to calculate HRV reactivity based on the absolute change of resting-state HRV to on-task HRV) was available.

### Information Sources

The databases Medline (EBSCO), Embase, Cochrane Library, CINAHL, Psycinfo, Web of Science, Scopus, and Pedro were consulted for publications up to Mai 2020 by a professional librarian of the University of Zurich.

### Search Strategy

In order to identify the key articles for the study objectives, a search strategy was developed based on the PICOS approach and predefined eligibility criteria. The search strategy was translated into precise search strings for each database in collaboration with a librarian. The search strings consisted of “Medical subject headings” (MeSH), free text words, and Boolean operators. They were constructed to combine predefined terms for population (e.g., adult), intervention (e.g., exercise, training, cognition, cognitive challenge, mental effort, and processing speed), outcome (e.g., autonomic nervous system, real-time HRV, cardiac autonomic response, and neuro-physiological measure), and study type (e.g., randomized controlled clinical trial, cross-over, and observational study). Within these groups, all terms were combined with OR operators. The search strings were applied without using further filtering options or limits. Consider Supplementary File A1 for a description of the complete search strategy including search strings.

### Study Selection and Data Collection

#### Data Management and Selection Process

All records were systematically screened using EPPI-Reviewer software (Version: 4.11.5.2) (Thomas et al., 2010). The provided standard coding scheme was adapted to meet all eligibility criteria. The screening- and selection process was pilot tested and executed by two independent reviewers (PM, MT) according to a predefined screening protocol (Supplementary File B). The retrieved results were matched and discussed for final inclusion by (PM / MT). In case of disagreements, EdB served as referee. By calculating Cohen's kappa, the strength of the inter-rater agreement of the study selection process was rated to be poor (0), slight (0.1–0.20), fair (0.21–0.40), moderate (0.41–0.60), substantial (0.61–0.80), or almost perfect (0.81–1.0) (Cohen, 1960; Landis and Koch, 1977; McHugh, 2012).

#### Data Collection Process

The EPPI-Reviewer software was used to then extract relevant data by two reviewers (PM, MT) (Thomas et al., 2010). The extracted data was cross-checked after completion of the data collection process. In case of mismatches, MA inspected the discrepancies and decided on the final data set.

### Data Items

Information was extracted from each included trial on: (1) study characteristics (i.e., author, year of publication, study design), (2) demographic characteristics of study participants (i.e., sample size, gender, age), (3) exercise characteristics [i.e., type, duration and intensity/complexity of the intervention(s)], (4) type and duration of HRV resting-state measurements, (5) HRV measurement technique and device, (6) controlling of confounders in each study, and (7) all reported HRV measures including statistically analyzed moderators or covariates (e.g., age, gender, fitness level, and cognitive functioning). The outcome measures that were considered for data synthesis included phasic HRV responses (in terms of HRV reactivity [i.e., the absolute change from resting state to on-task (i.e., absolute values of HRV measured during exercise) HRV] of time-domain HRV [i.e., mean of the time interval between two consecutive R waves on the electrocardiogram (mRR), standard deviation of NN intervals (SDNN), standard deviation of RR intervals (SDRR), percentage of successive RR intervals that differ by more than 50 ms (pNN50), root mean square of successive RR interval differences (RMSSD)], frequency-domain HRV (i.e., absolute power of the very-low-frequency (0.0033–0.04 Hz; VLF), low-frequency (0.04–0.15 Hz; LF) and the high-frequency (0.15–0.4 Hz; HF) band, relative power (in normal units) of the low-frequency (0.04–0.15 Hz; LFnu) and high-frequency (0.15–0.4 Hz; HFnu) band, and respiratory sinus arrhythmia (RSA)], as well as non-linear HRV measures [i.e., Poincaré plot standard deviations of the perpendicular line of identity (SD1) and along the line of identity (SD2), ratio of SD1 to SD2 (SD1/SD2), detrended fluctuation analyses which describe short-term fluctuations (DFA-α1), the sample entropy of successive R-R intervals (SampEn), as well as the Coefficient of Variation of R-R intervals (CoV)]. Confounders that were considered in the analysis of methodological quality were based on the selection of Laborde et al. (2017): (1) age and gender, (2) smoking, (3) habitual levels of alcohol consumption, (4) weight, height and waist-to-hip ratio, (5) cardioactive medication, such as antidepressants, antipsychotics or antihypertensives, (6) follow a normal sleep routine the day before the experiment, record the typical bedtime and typical waking time, (7) no intense physical training the day before the experiment, (8) no meal the last 2 h before the experiment, (9) no coffee—or caffeinated drinks such as energizing drinks—in the 2 h before the experiment, (10) questioning whether study participants needed to use the bathroom before the experiment begins, and (11) no alcohol for 24 h prior to the experiment (Laborde et al., 2017).

### Risk of Bias in Individual Studies

Methodological quality of all included studies was assessed by two independent raters (PM / MT) using the Quality Assessment Tool for Quantitative Studies (QATQS) of the Effective Public Health Practice Project assessment tool (EPHPP) and its corresponding guidelines (Thomas et al., 2004, 2020a,b). This tool was developed to evaluate the methodological quality of a variety of study designs, including randomized and non-randomized controlled trials, as well as observational studies (Thomas et al., 2004). The EPHPP was judged to be a suitable and reliable tool for systematic reviews and demonstrated content and construct validity (Deeks et al., 2003; Thomas et al., 2004). The tool comprises 14 items separated into six components: (1) sample selection, (2) study design, (3) identification and treatment of confounders, (4) blinding of outcome assessors and of participants, (5) reliability and validity of data collection methods, and (6) withdrawals and dropouts. Each component was rated “strong,” “moderate,” or “weak” according to objective criteria of the standardized guidelines and dictionary. After matching and discussing all assigned component ratings, the overall methodological quality of each study was defined and considered to be “strong” (i.e., no weak ratings), “moderate” (i.e., one weak rating) or “weak” (i.e., two or more weak ratings) (Thomas et al., 2004, 2020a,b). In case of disagreements, RHK served as referee.

### Data Synthesis

Conclusions were mainly drawn based on the findings of a qualitative synthesis of phasic HRV responses and its key moderating parameters (section Qualitative Synthesis). Additionally, the interpretations and conclusion were put in context and supported by a closer analysis of different types of exercises and between-group comparisons of different age-groups of the quantitative synthesis (section Quantitative Synthesis).

#### Qualitative Synthesis

A narrative synthesis of the included studies was conducted guided by Popay et al. (2006). Phasic responses of HRV as well as all reported and statistically analyzed moderators and covariates (e.g., age, gender, fitness level, and cognitive functioning) of phasic HRV responses (in terms of HRV reactivity (i.e., the absolute change from resting state to on-task (i.e., absolute values of HRV measured during exercise) HRV) were extracted from each included trial. Additionally, the statistical methods used to assess the analyzed moderators and covariates were summarized. Finally, all statistically analyzed moderators or covariates were summarized for each type of exercise (i.e., cognitive exercises, cardiorespiratory exercises, resistance exercises, and cognitive-motor training) to gain an overview of each moderating variable. The number, characteristics, and quality of studies reporting significant effects vs. no effect for each moderating variable were then considered for the reporting and interpreting of the results.

#### Quantitative Synthesis

Next to the qualitative synthesis, meta-analyses were performed to evaluate HRV reactivity to different types of exercises (i.e., cognitive exercises, cardiorespiratory exercises, resistance exercises, and cognitive-motor training) and to compare different age-groups (i.e., HOA vs. HA) on HRV reactivity.

Only studies with moderate to strong methodological quality and outcome measures meeting the standards of HRV measurement evaluated by a validated device were considered eligible for the quantitative synthesis (Electrophysiology TFotESoCtNASoP, 1996; Shaffer and Ginsberg, 2017). Outcome measures reflecting mainly cardiac vagal tone were included in hierarchical order: (1) RMSSD, (2) pNN50, (3) HFnu, (4) HF, and (5) SD1 (Electrophysiology TFotESoCtNASoP, 1996; Alvares et al., 2016; Ernst, 2017; Laborde et al., 2017; Shaffer and Ginsberg, 2017; Mika et al., 2020). Both, absolute and log-transformed values, were synthesized according to the Cochrane guidelines (Julian et al., 2019).

A pooled estimate was calculated for HRV reactivity by conducting a meta-analysis in R [R Version R 3.6.2 GUI 1.70 El Capitan build (7735) ( The R Foundation)] in line with RStudio Version 1.2.5033 (RStudio, Inc.) (Team, 2019) using a fixed-effects model of the “metaphor” package (Viechtbauer, 2010) to calculate standardized mean differences (Hedge's g) (Viechtbauer, 2010) and 95% confidence intervals (CI) between vagally-mediated HRV on-task and at resting state. Level of significance was set to *p* ≤ 0.05 and effect sizes were interpreted to be small (Hedge's g <0.5), medium (0.5 ≤ Hedge's g <0.8) or large (Hedge's g ≤ 0.8) (Cohen, 1988).

A planned subgroup analysis was performed for cognitive and physical (i.e., cardiorespiratory exercises and resistance training) exercises. Furthermore, to evaluate the effect of age, planned subgroup analyses were computed to compare on-task values of vagally-mediated HRV between HOA and healthy adults (i.e., mean age ≤ 50 years; HA).

### Risk of Bias Across Studies

Possible sources of heterogeneity among trials were investigated by using Cochrane Q in line with *I*^2^ statistics. In case of significant heterogeneity, indicated by significant Q-statistics (*p* <0.05), random-effect models were employed (Higgins and Thompson, 2002). To detect possible publication bias, funnel plots (i.e., standard error) were assessed both visually and formally with Egger's test (Egger et al., 1997; Sterne and Egger, 2001). When publication biases were indicated (i.e., Egger's regression test: *p* < 0.1), sensitivity analyses were performed by (1) comparing fixed- and random-effect models, and (2) applying the trim and fill method for random-effects models. The trim and fill method redresses funnel plot asymmetries by adjusting the point estimated of the pooled effect sizes and 95% CI for missing studies (Duval and Tweedie, 2000).

## Results

### Study Selection

**Figure 1:**
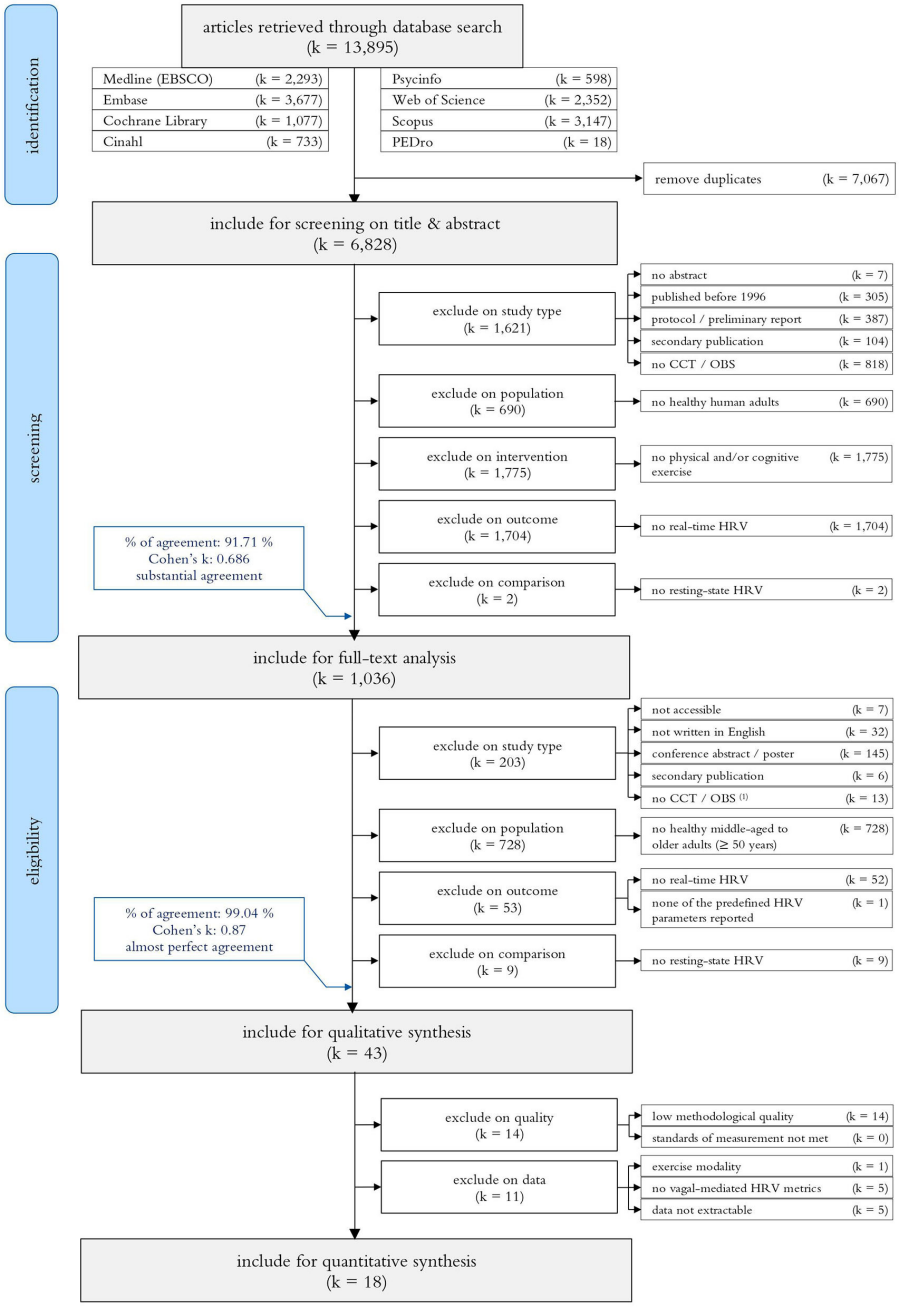
PRISM flow diagram of the search-, screening- and study selection process. CCT, Controlled Clinical Trial; OBS, Observational Study.

The systematic search for publications from 1996 up to Mai 2020 identified 13,895 records. After removing duplicates (*k* = 7,067) and screening on title and abstract (*k* = 6,828), 1,036 articles were included for a full text analysis on eligibility. The majority of the remaining studies were excluded on participant characteristics (i.e., no HOA with a mean age ≥50 years; *k* = 728). Additional studies were excluded on study report characteristics (*k* = 190), study type (*k* = 13), outcome measures (*k* = 53), or comparison (*k* = 9). The remaining 43 studies (Piepoli et al., 1996; Cacioppo et al., 2000; Perini et al., 2000; Steptoe et al., 2002, 2005; Wood et al., 2002; Bartels Matthew et al., 2003; Kunz-Ebrecht et al., 2003; Takahashi et al., 2003; Steptoe and Marmot, 2005, 2006; Davrath Linda et al., 2006; Hamer and Steptoe, 2007; Virtanen et al., 2007; Mayumi et al., 2008; Karavirta et al., 2009; Petrofsky et al., 2009; Dourado et al., 2010; Alves Naiane Ferraz et al., 2011; Millar et al., 2011; Sales et al., 2011; Wang Norman et al., 2011; Capuana et al., 2012; Archiza et al., 2013; Corrêa et al., 2013; Christensen Stephanie and Wright Heather, 2014; Collste et al., 2014; Lin et al., 2014, 2017a; Machado-Vidotti et al., 2014; Ahmadian and Dabidi Roshan, 2015; Crowley Olga et al., 2016; Norcliffe-Kaufmann et al., 2016; Wawrzyniak Andrew et al., 2016; Beer et al., 2017; Beer Noa et al., 2017; Betz Linda et al., 2017; Junior Adalberto et al., 2019; Kuraoka et al., 2019; Perpetuini et al., 2019; Rodrigues Jhennyfer et al., 2019; Wittstein et al., 2019; Kaltsatou et al., 2020) were included for the qualitative synthesis and—in case of sufficient quality and data reporting—considered for the meta-analyses (*k* = 18). Consider Figure 1 for a detailed overview of the study selection.

### Study Characteristics

All finally selected studies were conducted between 1996 (Piepoli et al., 1996) and 2020 (Kaltsatou et al., 2020), included sample sizes ranging between 7 (Takahashi et al., 2003) and 817 (Crowley Olga et al., 2016) HOA with mean ages between 50.7 (Wang Norman et al., 2011; Betz Linda et al., 2017)−85 (Wood et al., 2002) years. In total, 3,725 HOA (49.5 % females) with a mean age of 58.2 years were included. The majority of studies were designed as observational trials (Cacioppo et al., 2000; Perini et al., 2000; Steptoe et al., 2002, 2005; Wood et al., 2002; Bartels Matthew et al., 2003; Kunz-Ebrecht et al., 2003; Takahashi et al., 2003; Steptoe and Marmot, 2005, 2006; Davrath Linda et al., 2006; Hamer and Steptoe, 2007; Virtanen et al., 2007; Mayumi et al., 2008; Petrofsky et al., 2009; Dourado et al., 2010; Alves Naiane Ferraz et al., 2011; Sales et al., 2011; Wang Norman et al., 2011; Capuana et al., 2012; Archiza et al., 2013; Corrêa et al., 2013; Christensen Stephanie and Wright Heather, 2014; Collste et al., 2014; Lin et al., 2014, 2017a; Machado-Vidotti et al., 2014; Ahmadian and Dabidi Roshan, 2015; Crowley Olga et al., 2016; Norcliffe-Kaufmann et al., 2016; Wawrzyniak Andrew et al., 2016; Beer et al., 2017; Beer Noa et al., 2017; Betz Linda et al., 2017; Kuraoka et al., 2019; Perpetuini et al., 2019; Wittstein et al., 2019; Kaltsatou et al., 2020), of which 24 included a control population in form of a case-control trial design (Piepoli et al., 1996; Cacioppo et al., 2000; Wood et al., 2002; Bartels Matthew et al., 2003; Steptoe et al., 2005; Davrath Linda et al., 2006; Virtanen et al., 2007; Mayumi et al., 2008; Petrofsky et al., 2009; Alves Naiane Ferraz et al., 2011; Sales et al., 2011; Wang Norman et al., 2011; Capuana et al., 2012; Christensen Stephanie and Wright Heather, 2014; Collste et al., 2014; Ahmadian and Dabidi Roshan, 2015; Norcliffe-Kaufmann et al., 2016; Beer et al., 2017; Beer Noa et al., 2017; Betz Linda et al., 2017; Lin et al., 2017a; Perpetuini et al., 2019; Wittstein et al., 2019; Kaltsatou et al., 2020). Additionally, four studies were designed as controlled clinical trials (Karavirta et al., 2009; Millar et al., 2011; Junior Adalberto et al., 2019; Rodrigues Jhennyfer et al., 2019). The control populations included HA (*k* = 8) (Wood et al., 2002; Steptoe et al., 2005; Petrofsky et al., 2009; Capuana et al., 2012; Ahmadian and Dabidi Roshan, 2015; Kuraoka et al., 2019; Wittstein et al., 2019; Kaltsatou et al., 2020), as well as older adults with mNCD (*k* = 2) (Lin et al., 2017a; Perpetuini et al., 2019), neurological diseases (*k* = 2) (Beer et al., 2017; Beer Noa et al., 2017), cardiovascular diseases (*k* = 7) (Piepoli et al., 1996; Virtanen et al., 2007; Mayumi et al., 2008; Alves Naiane Ferraz et al., 2011; Wang Norman et al., 2011; Collste et al., 2014; Norcliffe-Kaufmann et al., 2016), respiratory tract diseases (*k* = 1) (Bartels Matthew et al., 2003), metabolic diseases (*k* = 2) (Petrofsky et al., 2009; Sales et al., 2011), psychological diseases (*k* = 1) (Betz Linda et al., 2017), or other clinical conditions (*k* = 1) (Christensen Stephanie and Wright Heather, 2014).

The reported study interventions consisted of cognitive exercises (*k* = 21) (Cacioppo et al., 2000; Steptoe et al., 2002, 2005; Wood et al., 2002; Kunz-Ebrecht et al., 2003; Steptoe and Marmot, 2005, 2006; Hamer and Steptoe, 2007; Capuana et al., 2012; Christensen Stephanie and Wright Heather, 2014; Collste et al., 2014; Lin et al., 2014, 2017a; Crowley Olga et al., 2016; Norcliffe-Kaufmann et al., 2016; Wawrzyniak Andrew et al., 2016; Beer et al., 2017; Beer Noa et al., 2017; Betz Linda et al., 2017; Kuraoka et al., 2019; Perpetuini et al., 2019), cardiorespiratory exercises (*k* = 18) (Perini et al., 2000; Bartels Matthew et al., 2003; Takahashi et al., 2003; Davrath Linda et al., 2006; Virtanen et al., 2007; Mayumi et al., 2008; Karavirta et al., 2009; Dourado et al., 2010; Alves Naiane Ferraz et al., 2011; Sales et al., 2011; Wang Norman et al., 2011; Corrêa et al., 2013; Ahmadian and Dabidi Roshan, 2015; Beer et al., 2017; Junior Adalberto et al., 2019; Rodrigues Jhennyfer et al., 2019; Wittstein et al., 2019; Kaltsatou et al., 2020), resistance exercises (*k* = 6) (Piepoli et al., 1996; Petrofsky et al., 2009; Millar et al., 2011; Machado-Vidotti et al., 2014; Beer et al., 2017; Beer Noa et al., 2017), inspiratory loaded breathing exercise (*k* = 1) (Archiza et al., 2013), and simultaneous cognitive-motor training (*k* = 2) (Beer et al., 2017; Beer Noa et al., 2017). Among the cognitive exercises, the study interventions included paper-and-pencil and computerized versions of mental arithmetic (*k* = 9) (Cacioppo et al., 2000; Collste et al., 2014; Lin et al., 2014, 2017a; Crowley Olga et al., 2016; Beer et al., 2017; Beer Noa et al., 2017; Betz Linda et al., 2017; Kuraoka et al., 2019), Stroop color-word (*k* = 10) (Steptoe et al., 2002; Kunz-Ebrecht et al., 2003; Steptoe and Marmot, 2005, 2006; Hamer and Steptoe, 2007; Lin et al., 2014, 2017a; Crowley Olga et al., 2016; Norcliffe-Kaufmann et al., 2016; Wawrzyniak Andrew et al., 2016), mirror tracing (*k* = 7) (Steptoe et al., 2002; Kunz-Ebrecht et al., 2003; Steptoe and Marmot, 2005, 2006; Hamer and Steptoe, 2007; Wawrzyniak Andrew et al., 2016; Kuraoka et al., 2019), Go-NoGo (*k* = 1) (Capuana et al., 2012), working memory inhibitory control (*k* = 1) (Capuana et al., 2012), reaction time (*k* = 1) (Wood et al., 2002) and Rey–Osterrieth complex figure (*k* = 1) (Perpetuini et al., 2019) tasks administered for durations between one (Cacioppo et al., 2000; Beer et al., 2017; Beer Noa et al., 2017)−10 min (Lin et al., 2017a). Additionally, Steptoe et al. (2005) administered three tasks of the Wechsler Memory Scale (WMS) (Steptoe et al., 2005). Regarding the cardiorespiratory exercises, incremental ramp tests on bicycle ergometers (*k* = 6) (Bartels Matthew et al., 2003; Virtanen et al., 2007; Mayumi et al., 2008; Karavirta et al., 2009; Sales et al., 2011; Ahmadian and Dabidi Roshan, 2015) and treadmills (*k* = 3) (Takahashi et al., 2003; Davrath Linda et al., 2006; Rodrigues Jhennyfer et al., 2019) were most frequently performed. Four studies implemented constant load cycling (*k* = 6) (Perini et al., 2000; Alves Naiane Ferraz et al., 2011; Wang Norman et al., 2011; Beer et al., 2017; Beer Noa et al., 2017; Kaltsatou et al., 2020) or walking (*k* = 2) (Corrêa et al., 2013; Wittstein et al., 2019) exercises with durations ranging from 2 min (Beer et al., 2017) to 16 min (Wang Norman et al., 2011) at moderate (Alves Naiane Ferraz et al., 2011; Wang Norman et al., 2011; Beer et al., 2017; Beer Noa et al., 2017; Wittstein et al., 2019; Kaltsatou et al., 2020) up to vigorous (Perini et al., 2000; Corrêa et al., 2013) intensities. Resistance exercise included handgrip (*k* = 5) (Piepoli et al., 1996; Petrofsky et al., 2009; Millar et al., 2011; Beer et al., 2017; Beer Noa et al., 2017), bench press (*k* = 1) (Machado-Vidotti et al., 2014), and leg press (*k* = 1) (Machado-Vidotti et al., 2014) exercises. Handgrip exercises consisted of static (Petrofsky et al., 2009; Millar et al., 2011; Beer et al., 2017; Beer Noa et al., 2017), repetitive (Piepoli et al., 1996) and/or intermitted (Petrofsky et al., 2009; Millar et al., 2011) isometric handgrip exercise protocols at intensities between 10% (Petrofsky et al., 2009) and 50% (Piepoli et al., 1996) of maximal voluntary contraction for 16 × 30 s (15 s rest) (Millar et al., 2011) up to 4 × 2 min (1 min rest) (Millar et al., 2011). Machado-Vidotti et al. (2014) applied an incremental bench- and leg press exercise test starting at an exercise load of 10% one-repetition maximum continuing with stepwise increases until exhaustion at 12 repetitions per minute and a controlled breathing pattern (Machado-Vidotti et al., 2014).

Most of the studies assessed R-R intervals using laboratory ECG machines (*k* = 16) (Cacioppo et al., 2000; Wood et al., 2002; Bartels Matthew et al., 2003; Takahashi et al., 2003; Davrath Linda et al., 2006; Virtanen et al., 2007; Alves Naiane Ferraz et al., 2011; Millar et al., 2011; Capuana et al., 2012; Christensen Stephanie and Wright Heather, 2014; Ahmadian and Dabidi Roshan, 2015; Crowley Olga et al., 2016; Norcliffe-Kaufmann et al., 2016; Betz Linda et al., 2017; Lin et al., 2017a; Wittstein et al., 2019) or portable ECG measurement devices (*k* = 13) (Steptoe et al., 2002, 2005; Kunz-Ebrecht et al., 2003; Steptoe and Marmot, 2005, 2006; Hamer and Steptoe, 2007; Collste et al., 2014; Wawrzyniak Andrew et al., 2016; Beer et al., 2017; Beer Noa et al., 2017; Kuraoka et al., 2019; Rodrigues Jhennyfer et al., 2019; Kaltsatou et al., 2020). Other studies relied on portable devices measuring pulse-wave frequencies based on photoplethysmography (*k* = 7) (Karavirta et al., 2009; Dourado et al., 2010; Sales et al., 2011; Archiza et al., 2013; Corrêa et al., 2013; Machado-Vidotti et al., 2014; Junior Adalberto et al., 2019), functional infrared imaging (*k* = 1) (Perpetuini et al., 2019), or didn't report the exact measurement device (*k* = 6) (Piepoli et al., 1996; Perini et al., 2000; Mayumi et al., 2008; Petrofsky et al., 2009; Wang Norman et al., 2011; Lin et al., 2014). The portable measurement devices included different models of the Polar heart rate monitor watches [i.e., RS800(CX) (*k* = 4) (Corrêa et al., 2013; Beer et al., 2017; Beer Noa et al., 2017; Junior Adalberto et al., 2019), S810i (*k* = 4) (Karavirta et al., 2009; Dourado et al., 2010; Sales et al., 2011; Archiza et al., 2013), Team Pro Sensor (*k* = 1) (Rodrigues Jhennyfer et al., 2019), and Vantage (*k* = 1) (Machado-Vidotti et al., 2014)], and ambulatory ECG systems including the VU University Ambulatory Monitoring System (VU-AMS; *k* = 6) (Steptoe et al., 2002, 2005; Kunz-Ebrecht et al., 2003; Steptoe and Marmot, 2005, 2006; Hamer and Steptoe, 2007), Holter Monitors (*k* = 2) (Collste et al., 2014; Kaltsatou et al., 2020), the ActiHeart monitoring device (*k* = 1) (Wawrzyniak Andrew et al., 2016), the Nihon Kohden Pocket ECG Monitor WEC-7101 (*k* = 1) (Kuraoka et al., 2019), and the Custo Cardio 100 (*k* = 1) (Ahmadian and Dabidi Roshan, 2015). Resting-state HRV was extracted from baseline measurements with durations of 1 min (Virtanen et al., 2007; Corrêa et al., 2013; Ahmadian and Dabidi Roshan, 2015)−30 min (Piepoli et al., 1996) conducted in a seated (*k* = 25) (Cacioppo et al., 2000; Perini et al., 2000; Wood et al., 2002; Bartels Matthew et al., 2003; Kunz-Ebrecht et al., 2003; Steptoe and Marmot, 2005; Davrath Linda et al., 2006; Virtanen et al., 2007; Petrofsky et al., 2009; Wang Norman et al., 2011; Capuana et al., 2012; Archiza et al., 2013; Christensen Stephanie and Wright Heather, 2014; Lin et al., 2014; Machado-Vidotti et al., 2014; Ahmadian and Dabidi Roshan, 2015; Crowley Olga et al., 2016; Wawrzyniak Andrew et al., 2016; Beer et al., 2017; Beer Noa et al., 2017; Betz Linda et al., 2017; Kuraoka et al., 2019; Rodrigues Jhennyfer et al., 2019; Wittstein et al., 2019; Kaltsatou et al., 2020), supine (*k* = 6) (Piepoli et al., 1996; Perini et al., 2000; Karavirta et al., 2009; Alves Naiane Ferraz et al., 2011; Millar et al., 2011; Norcliffe-Kaufmann et al., 2016) or upright (*k* = 1) (Takahashi et al., 2003) posture. For a more detailed overview of the study characteristics, consider Appendix A.

### Risk of Bias Within Studies

With 67% of studies rated “moderate” to “strong” (Table 1), the average methodological quality of the selected studies was reasonable. Twenty studies exhibited a possible selection bias caused by a lack of reporting of the selection process (Piepoli et al., 1996; Steptoe et al., 2002; Wood et al., 2002; Kunz-Ebrecht et al., 2003; Takahashi et al., 2003; Mayumi et al., 2008; Petrofsky et al., 2009; Dourado et al., 2010; Alves Naiane Ferraz et al., 2011; Sales et al., 2011; Wang Norman et al., 2011; Archiza et al., 2013; Corrêa et al., 2013; Machado-Vidotti et al., 2014; Ahmadian and Dabidi Roshan, 2015; Norcliffe-Kaufmann et al., 2016; Beer et al., 2017; Beer Noa et al., 2017; Junior Adalberto et al., 2019; Kuraoka et al., 2019; Kaltsatou et al., 2020). Strong study designs included randomized controlled trials (*k* = 2) (Karavirta et al., 2009; Junior Adalberto et al., 2019) and controlled clinical trials (*k* = 2) (Millar et al., 2011; Rodrigues Jhennyfer et al., 2019), while observational studies were assigned to “moderate” study designs (Piepoli et al., 1996; Cacioppo et al., 2000; Perini et al., 2000; Steptoe et al., 2002, 2005; Wood et al., 2002; Bartels Matthew et al., 2003; Kunz-Ebrecht et al., 2003; Takahashi et al., 2003; Steptoe and Marmot, 2005, 2006; Davrath Linda et al., 2006; Hamer and Steptoe, 2007; Virtanen et al., 2007; Mayumi et al., 2008; Petrofsky et al., 2009; Dourado et al., 2010; Alves Naiane Ferraz et al., 2011; Sales et al., 2011; Wang Norman et al., 2011; Capuana et al., 2012; Archiza et al., 2013; Corrêa et al., 2013; Christensen Stephanie and Wright Heather, 2014; Collste et al., 2014; Lin et al., 2014, 2017a; Machado-Vidotti et al., 2014; Ahmadian and Dabidi Roshan, 2015; Crowley Olga et al., 2016; Norcliffe-Kaufmann et al., 2016; Wawrzyniak Andrew et al., 2016; Beer et al., 2017; Beer Noa et al., 2017; Betz Linda et al., 2017; Kuraoka et al., 2019; Perpetuini et al., 2019; Wittstein et al., 2019; Kaltsatou et al., 2020). The controlling of confounders in each study is summarized in Appendix A. Studies were rated “strong” if they had no important between-group differences before the measurements or controlled ≥80% of predefined confounding variables (*k* = 19) (Perini et al., 2000; Steptoe et al., 2002; Kunz-Ebrecht et al., 2003; Takahashi et al., 2003; Steptoe and Marmot, 2005, 2006; Hamer and Steptoe, 2007; Karavirta et al., 2009; Dourado et al., 2010; Millar et al., 2011; Archiza et al., 2013; Corrêa et al., 2013; Lin et al., 2014; Machado-Vidotti et al., 2014; Crowley Olga et al., 2016; Wawrzyniak Andrew et al., 2016; Junior Adalberto et al., 2019; Kuraoka et al., 2019; Rodrigues Jhennyfer et al., 2019), “moderate” for ≥60% (*k* = 2) (Cacioppo et al., 2000; Kaltsatou et al., 2020), and “weak” for <60% (*k* = 22) (Piepoli et al., 1996; Wood et al., 2002; Bartels Matthew et al., 2003; Steptoe et al., 2005; Davrath Linda et al., 2006; Virtanen et al., 2007; Mayumi et al., 2008; Petrofsky et al., 2009; Alves Naiane Ferraz et al., 2011; Sales et al., 2011; Wang Norman et al., 2011; Capuana et al., 2012; Christensen Stephanie and Wright Heather, 2014; Collste et al., 2014; Ahmadian and Dabidi Roshan, 2015; Norcliffe-Kaufmann et al., 2016; Beer et al., 2017; Beer Noa et al., 2017; Betz Linda et al., 2017; Lin et al., 2017a; Perpetuini et al., 2019; Wittstein et al., 2019). Only one study reported blinding of participants or outcome assessors (*k* = 1) (Archiza et al., 2013). Notably, 31 out of 43 studies either used a laboratory ECG machine (*k* = 16) (Cacioppo et al., 2000; Wood et al., 2002; Bartels Matthew et al., 2003; Takahashi et al., 2003; Davrath Linda et al., 2006; Virtanen et al., 2007; Alves Naiane Ferraz et al., 2011; Millar et al., 2011; Capuana et al., 2012; Christensen Stephanie and Wright Heather, 2014; Ahmadian and Dabidi Roshan, 2015; Crowley Olga et al., 2016; Norcliffe-Kaufmann et al., 2016; Betz Linda et al., 2017; Lin et al., 2017a; Wittstein et al., 2019) or reported the validity and reliability of their measurement device (*k* = 15) (Piepoli et al., 1996; Perini et al., 2000; Steptoe et al., 2002, 2005; Kunz-Ebrecht et al., 2003; Steptoe and Marmot, 2005, 2006; Hamer and Steptoe, 2007; Petrofsky et al., 2009; Wang Norman et al., 2011; Archiza et al., 2013; Collste et al., 2014; Lin et al., 2014; Kuraoka et al., 2019; Kaltsatou et al., 2020) and were rated “strong” in their data collection method. In 10 studies, the measurement device for HRV was only reported to be valid (*k* = 5) (Karavirta et al., 2009; Sales et al., 2011; Wawrzyniak Andrew et al., 2016; Beer et al., 2017; Beer Noa et al., 2017) or the validity and reliability properties were not described within the study (*k* = 5) (Dourado et al., 2010; Corrêa et al., 2013; Machado-Vidotti et al., 2014; Junior Adalberto et al., 2019; Rodrigues Jhennyfer et al., 2019) but was derivable from secondary literature (Dobbs et al., 2019). Perpetuini et al. (2019) used functional infrared imaging (fIRI) signals to calculate HRV. This technology was mentioned to be “not particularly strong” (Perpetuini et al., 2019), but was still used, since “it indeed shows the existence of a functional relation between fIRI and cardiac activity” (Perpetuini et al., 2019). Nonetheless, validity and reliability of this method has not been ensured which resulted in a “weak” rating. Mayumi et al. (2008) failed to report data collection tools for HRV which also resulted in a “weak” rating (Mayumi et al., 2008). Reporting withdrawals and drop-outs was complete with follow-up rates >80% (*k* = 11) (Steptoe et al., 2002; Steptoe and Marmot, 2005; Karavirta et al., 2009; Millar et al., 2011; Sales et al., 2011; Collste et al., 2014; Machado-Vidotti et al., 2014; Betz Linda et al., 2017; Junior Adalberto et al., 2019; Rodrigues Jhennyfer et al., 2019; Kaltsatou et al., 2020). Four studies didn't report the percentage of participants completing the study (*k* = 4) (Takahashi et al., 2003; Alves Naiane Ferraz et al., 2011; Wang Norman et al., 2011; Wittstein et al., 2019) resulting in a “weak” rating. The majority of studies were rated “moderate,” since a reporting of withdrawals and drop-outs was not applicable (i.e., one-time measurements; Cacioppo et al., 2000; Perini et al., 2000; Steptoe et al., 2002; Wood et al., 2002; Bartels Matthew et al., 2003; Kunz-Ebrecht et al., 2003; Steptoe and Marmot, 2005, 2006; Davrath Linda et al., 2006; Hamer and Steptoe, 2007; Virtanen et al., 2007; Mayumi et al., 2008; Petrofsky et al., 2009; Dourado et al., 2010; Capuana et al., 2012; Archiza et al., 2013; Corrêa et al., 2013; Christensen Stephanie and Wright Heather, 2014; Lin et al., 2014, 2017a; Ahmadian and Dabidi Roshan, 2015; Crowley Olga et al., 2016; Norcliffe-Kaufmann et al., 2016; Wawrzyniak Andrew et al., 2016; Beer et al., 2017; Beer Noa et al., 2017; Kuraoka et al., 2019; Perpetuini et al., 2019).

**Table 1:**
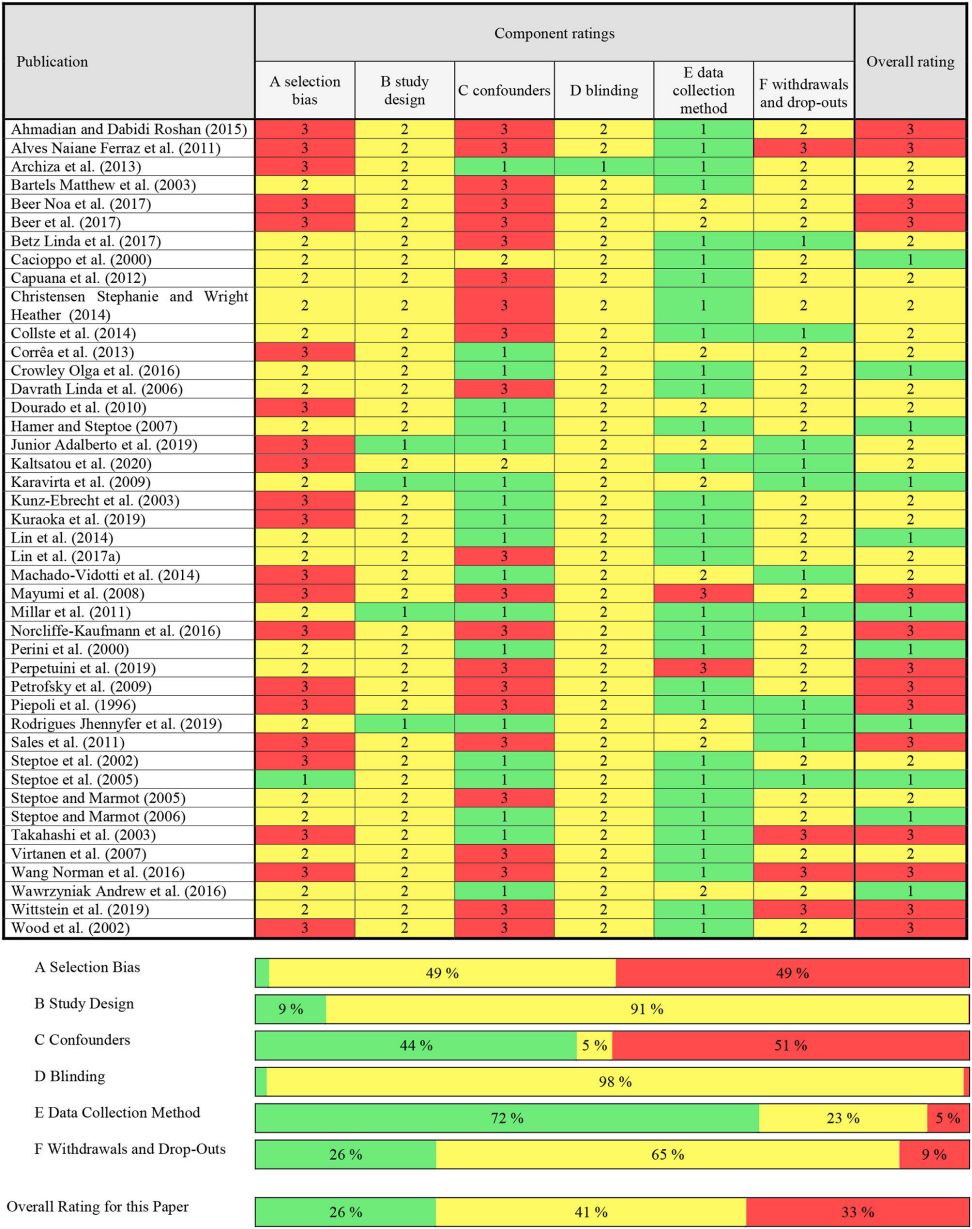
Assessment of methodological quality on basis of the quality assessment tool for quantitative studies (QATQA).

### Qualitative Synthesis

The phasic responses of HRV as well as reported moderator variables to cognitive and physical exercises as well as cognitive-motor training are summarized in Table 2. The results of each individual study are summarized in Appendix B. Forty-one out of the 43 studies reported at least one time- or frequency-domain variable of HRV, with RMSSD (*k* = 20) (Steptoe et al., 2002, 2005; Kunz-Ebrecht et al., 2003; Steptoe and Marmot, 2005, 2006; Hamer and Steptoe, 2007; Virtanen et al., 2007; Millar et al., 2011; Sales et al., 2011; Wang Norman et al., 2011; Archiza et al., 2013; Corrêa et al., 2013; Machado-Vidotti et al., 2014; Ahmadian and Dabidi Roshan, 2015; Norcliffe-Kaufmann et al., 2016; Wawrzyniak Andrew et al., 2016; Beer et al., 2017; Beer Noa et al., 2017; Junior Adalberto et al., 2019; Rodrigues Jhennyfer et al., 2019) and HF (*k* = 19) (Piepoli et al., 1996; Perini et al., 2000; Wood et al., 2002; Bartels Matthew et al., 2003; Takahashi et al., 2003; Davrath Linda et al., 2006; Mayumi et al., 2008; Karavirta et al., 2009; Alves Naiane Ferraz et al., 2011; Wang Norman et al., 2011; Archiza et al., 2013; Lin et al., 2014, 2017a; Machado-Vidotti et al., 2014; Crowley Olga et al., 2016; Norcliffe-Kaufmann et al., 2016; Kuraoka et al., 2019; Rodrigues Jhennyfer et al., 2019; Kaltsatou et al., 2020) being the most frequently reported parameters.

**Table 2:** Summary table of HRV reactivity and its moderators in healthy middle-aged to older adults (≥50 years).

**Exercise**	**HRV reactivity**																			**Moderators of HRV responses**		
	**Time-domain**					**Frequency-domain**							**Non-linear**							**Significant relation (** ***p*** **<** **0.05)**		**No effect**
**Type**	**mRR [ms]**	**SDNN [ms]**	**SDRR [ms]**	**pNN50 [%]**	**RMSSD [ms]**	**VLF [ms^**2**^]**	**LF [ms^**2**^]**	**LFnu [nu]**	**RSA [ms^**2**^]**	**HF [ms^**2**^]**	**HFnu [nu]**	**LF/HF [%]**	**SD1 [ms]**	**SD2 [ms]**	**SD1/SD2 [%]**	**DFα1 []**	**SampEn**	**CoV**	**References**	**Variable**	**Relations**	**Variable**
Cognitive Tasks		↑^1^						↑^1^			↑^1^								Wood et al., 2002; Collste et al., 2014; Perpetuini et al., 2019	Age Brain Activity Cognition HRV (rest) Modality Physical Fitness Stress Response Task Difficulty	Aging was associated with blunted HF reactivities (Crowley Olga et al., 2016). Anterior cingulate cortex hyperactivity (as a compensatory mechanism for neurodegeneration in frontal regions) resulted in stronger HF declines during the tasks (Lin et al., 2017a). Better cognition was related to lower reactivities in HF (Lin et al., 2017a) and RMSSD (Wawrzyniak Andrew et al., 2016), as well as higher reactivities in LF (Lin et al., 2014). Higher HRV at rest was related to higher HF on-task (Crowley Olga et al., 2016). HRV reactivity (i.e. HF and LF) varied between cognitive task modalities [i.e. Stroop > mental arithmetic (Lin et al., 2014), mental arithmetic > mirror tracing (Kuraoka et al., 2019)] Higher physical fitness was related to lower reactivities of RMSSD (Hamer and Steptoe, 2007). Higher subjective strain was related to larger reductions in SDNN (Betz Linda et al., 2017) and RMSSD (Kunz-Ebrecht et al., 2003), whereas larger reactivities in RMSSD were associated with greater increases of TNF-α (Hamer and Steptoe, 2007) and cortisol (Kunz-Ebrecht et al., 2003). Higher task difficulty was related to higher LF reactivity (Christensen Stephanie and Wright Heather, 2014).	Gender [i.e. RMSSD (Kunz-Ebrecht et al., 2003; Steptoe and Marmot, 2005)] Cognition [i.e. SDNN (Beer Noa et al., 2017), RSA (Capuana et al., 2012), and HF (Lin et al., 2014)] Heart rate [i.e. SDNN (Betz Linda et al., 2017)] Level of physical activity [i.e. RMSSD (Steptoe et al., 2002)] Respiration (i.e. HF; Crowley Olga et al., 2016) Smoking and Alcohol Consumption [i.e. RMSSD (Steptoe et al., 2002)] Stress [i.e. IL-6 response; RMSSD (Hamer and Steptoe, 2007)] Task type [i.e. verbal vs. spatial; LF (Christensen Stephanie and Wright Heather, 2014)]
		→^1^			→^4^				→^1^			→^1^							Steptoe and Marmot, 2005, 2006; Capuana et al., 2012; Beer et al., 2017; Beer Noa et al., 2017; Betz Linda et al., 2017; Kuraoka et al., 2019			
	↘^1^	↘^1^			↘^1^														Steptoe et al., 2002; Norcliffe-Kaufmann et al., 2016; Beer Noa et al., 2017			
	↓^2^	↓^2^			↓^4^		↓^3^		↓^1^	↓^4^	↓^1^								Cacioppo et al., 2000; Wood et al., 2002; Kunz-Ebrecht et al., 2003; Steptoe et al., 2005; Hamer and Steptoe, 2007; Christensen Stephanie and Wright Heather, 2014; Lin et al., 2014, 2017a; Crowley Olga et al., 2016; Wawrzyniak Andrew et al., 2016; Beer et al., 2017; Beer Noa et al., 2017; Junior Adalberto et al., 2019; Kuraoka et al., 2019			
Cardiorespiratory Exercise							↑^1^	↑^1^			↑^1^	↑^1^							Perini et al., 2000; Bartels Matthew et al., 2003	Age Body fat Intensity Modality	Aging was associated with increased mRR intervals (but not mRR reactivity; Corrêa et al., 2013) and suppressed levels of LF (Kaltsatou et al., 2020), LF/HF (Kaltsatou et al., 2020) and DFA-α1 (Karavirta et al., 2009; Kaltsatou et al., 2020) during exercise. Age-related changes in HRV vanished when controlling for body fat (Kaltsatou et al., 2020). Higher intensities of exercise and higher heart rate were related to larger reductions of mRR (Virtanen et al., 2007), HF (Mayumi et al., 2008; Archiza et al., 2013), LFnu (Perini et al., 2000), LF/HF (Perini et al., 2000), and DFA-α1 (Karavirta et al., 2009) as well as larger increases in HFnu (Perini et al., 2000) during exercise. The addition of gait synchronization elevated DFA-α during walking (Wittstein et al., 2019).	Age [i.e. RMSSD (Corrêa et al., 2013), VLF (Kaltsatou et al., 2020), LF(nu) (Bartels Matthew et al., 2003), HF(nu) (Bartels Matthew et al., 2003; Kaltsatou et al., 2020), LF/HF (Bartels Matthew et al., 2003), SD1 (Corrêa et al., 2013; Kaltsatou et al., 2020), SD2 (Corrêa et al., 2013; Kaltsatou et al., 2020), DFA-α1 (Kaltsatou et al., 2020), and CoV (Kaltsatou et al., 2020)] Gender [i.e. mRR (Corrêa et al., 2013), RMSSD (Corrêa et al., 2013), LF(nu) (Perini et al., 2000; Bartels Matthew et al., 2003), HF(nu) (Perini et al., 2000; Bartels Matthew et al., 2003; Takahashi et al., 2003), LF/HF (Perini et al., 2000; Bartels Matthew et al., 2003), SD1 (Corrêa et al., 2013), and SD2 (Corrêa et al., 2013)] Body mass index [i.e. LF(nu) (Bartels Matthew et al., 2003), HF(nu) (Bartels Matthew et al., 2003), and LF/HF (Bartels Matthew et al., 2003)] Intensity [i.e. mRR (Wittstein et al., 2019), SDRR (Wittstein et al., 2019), HF (Takahashi et al., 2003), CoV (Wittstein et al., 2019), and DFA-α1 (Wittstein et al., 2019)] Physical fitness (i.e. DFA-α1; Karavirta et al., 2009)
							↗^1^			↗^1^		↗^1^		↗^1^					Rodrigues Jhennyfer et al., 2019; Kaltsatou et al., 2020			
		→^1^				→^1^	→^1^	→^1^		→^1^	→^2^		→^1^						Bartels Matthew et al., 2003; Alves Naiane Ferraz et al., 2011; Beer et al., 2017; Kaltsatou et al., 2020			
				↘^1^	↘^1^					↘^1^		↘^2^	↘^1^			↘^1^			Dourado et al., 2010; Alves Naiane Ferraz et al., 2011; Ahmadian and Dabidi Roshan, 2015; Rodrigues Jhennyfer et al., 2019; Kaltsatou et al., 2020			
	↓^4^	↓^2^			↓^4^		↓^1^	↓^1^		↓^3^		↓^1^	↓^2^	↓^2^		↓^1^			Perini et al., 2000; Takahashi et al., 2003; Davrath Linda et al., 2006; Virtanen et al., 2007; Mayumi et al., 2008; Karavirta et al., 2009; Wang Norman et al., 2011; Corrêa et al., 2013; Ahmadian and Dabidi Roshan, 2015; Beer et al., 2017			
Resistance Exercise								↗^1^				↗^1^							Machado-Vidotti et al., 2014	Duration Intensity Modality	SampEn was reduced for longer durations (at constant load) (Millar et al., 2011). HRV reactivity increased with increasing exercise loads (Machado-Vidotti et al., 2014) (i.e. RMSSD, HF, HFnu and SD1 decrease, LFnu and LF/HF increase). HRV reactivity (i.e. LFnu and HF) was more pronounced to upper- than lower limb exercise (Machado-Vidotti et al., 2014).	Duration (i.e. SDNN, RMSSD, pNN50, and DFA-α1; Millar et al., 2011)
		→^1^		→^1^	→^3^		→^1^									→^1^		→^1^	Piepoli et al., 1996; Petrofsky et al., 2009; Millar et al., 2011; Beer Noa et al., 2017			
		↘^1^																	Beer Noa et al., 2017			
	↓^2^	↓^1^			↓^1^					↓^2^	↓^1^		↓^1^				↓^1^		Piepoli et al., 1996; Millar et al., 2011; Machado-Vidotti et al., 2014; Beer Noa et al., 2017			
Simultaneous cognitive-motor training		→^1^																	Beer et al., 2017	NR	NR	NR
		↘^1^																	Beer Noa et al., 2017			
	↓^1^				↓^2^														Beer et al., 2017; Beer Noa et al., 2017			

*↑^n^ = significant (p < 0.05) increase from resting HRV, ↗^n^ = increasing trend (not significant or not statistically tested) → ^n^ = no significant change from resting HRV, ↘^n^ = decreasing trend (not significant or not statistically tested), ↓^n^ = significant (p < 0.05) decrease from resting HRV, n = number of studies reporting this effect, NR = not reported*.

#### Phasic HRV Responses to Cognitive Exercises

The vast majority of studies reported decreases in vagally-mediated HRV (i.e., RMSSD (*k* = 5) [Steptoe et al., 2002; Kunz-Ebrecht et al., 2003; Steptoe and Marmot, 2005; Hamer and Steptoe, 2007; Wawrzyniak Andrew et al., 2016), HFnu (*k* = 1) (Wood et al., 2002)], and HF (*k* = 4) [Lin et al., 2014, 2017a; Crowley Olga et al., 2016; Kuraoka et al., 2019)] during cognitive exercises. Additionally, decreases in primarily sympathetic or mixed parasympathetic-sympathetic HRV indices (HRV_mixed_) were reported (*k* = 8) [Cacioppo et al., 2000; Wood et al., 2002; Christensen Stephanie and Wright Heather, 2014; Lin et al., 2014; Norcliffe-Kaufmann et al., 2016; Beer et al., 2017; Beer Noa et al., 2017; Kuraoka et al., 2019)].

Moderating effects of demographic variables on phasic HRV responses were assessed by five studies. The reactivity of vagally-mediated HRV was blunted at higher age [i.e., HF (Crowley Olga et al., 2016)] and with increased physical fitness levels [i.e., RMSSD (Hamer and Steptoe, 2007)], whereas no influences of gender [i.e., RMSSD (Kunz-Ebrecht et al., 2003; Steptoe and Marmot, 2005)] and other lifestyle factors [i.e., smoking, alcohol consumption and physical activity; RMSSD (Steptoe et al., 2002)] were found. When additionally considering comparisons of different age-groups (i.e., HOA vs. HA), three studies reported significant between-group differences: HOA exhibited lower HRV_mixed_ on-task (Wood et al., 2002) as well as blunted reactivities of vagally-mediated HRV [i.e., RMSSD (Steptoe et al., 2005)]. In contrast, two studies showed no between-group differences in vagally-mediated HRV [i.e., HFnu (Wood et al., 2002), lnHF (Kuraoka et al., 2019)] and HRV_mixed_ (Wood et al., 2002; Kuraoka et al., 2019) on-task, as well as the reactivity of HRV_mixed_ (Capuana et al., 2012). The relation of the reactivity of vagally-mediated HRV and cognition was analyzed by multiple studies. Higher values of vagally-mediated HRV on-task were associated with higher HRV at rest [i.e., HF (Crowley Olga et al., 2016)], while larger withdrawals of vagally-mediated HRV predicted worse cognition [i.e., global cognition (i.e., HF (Lin et al., 2017a)] and processing speed [i.e., RMSSD (Wawrzyniak Andrew et al., 2016)], but not executive functioning [i.e., HF (Lin et al., 2014, 2017a)]. Looking at specific brain regions, anterior cingulate cortex hyperactivity (as a compensatory mechanism for neurodegeneration in frontal regions) resulted in high levels of HF at rest, as well as stronger HF declines during cognitive exercises (Lin et al., 2017a). Resting and on-task values of HRV_mixed_ were not related to cognitive functioning [i.e., SDNN (Beer Noa et al., 2017), RSA (Capuana et al., 2012)], but higher reactivities of LF predicted better executive functioning [i.e., LF (Lin et al., 2014)]. Task demands and the individual responses to the cognitive challenges also moderated HRV reactivity: Higher task difficulties and a higher subjective strain were related to larger withdrawals of vagally-mediated HRV [i.e., RMSSD (Kunz-Ebrecht et al., 2003)] and HRV_mixed_ [i.e., SDNN (Betz Linda et al., 2017), LF (Christensen Stephanie and Wright Heather, 2014)]. Larger withdrawals of vagally-mediated HRV were additionally accompanied by greater increases of TNF-α [i.e., RMSSD (Hamer and Steptoe, 2007)] and cortisol [i.e., RMSSD (Kunz-Ebrecht et al., 2003)], but not IL-6 [i.e., RMSSD (Hamer and Steptoe, 2007)]. Moreover, HRV reactivity was moderated by the task modality: According to Lin et al. (2014), the Stroop color-word task induced significantly larger HF- and LF reactivities than mental arithmetic test (Lin et al., 2014), whereas Christensen Stephanie and Wright Heather (2014) reported no differences in LF between a verbal and spatial n-back task (Christensen Stephanie and Wright Heather, 2014).

#### Phasic HRV Responses to Cardiorespiratory Exercises

Despite the large heterogeneity of interventions and HRV measurement and analysis methodologies, consistent patterns in phasic HRV responses during cardiorespiratory exercises were identified: Decreases in vagally-mediated HRV indices were observed compared to rest, particularly in RMSSD (*k* = 5) (Virtanen et al., 2007; Corrêa et al., 2013; Ahmadian and Dabidi Roshan, 2015; Beer et al., 2017; Rodrigues Jhennyfer et al., 2019), HF (*k* = 4) (Takahashi et al., 2003; Davrath Linda et al., 2006; Mayumi et al., 2008; Rodrigues Jhennyfer et al., 2019), and SD1 (*k* = 3) (Virtanen et al., 2007; Dourado et al., 2010; Corrêa et al., 2013). Additionally, decreases in HRV_mixed_ (*k* = 6) (Virtanen et al., 2007; Karavirta et al., 2009; Corrêa et al., 2013; Ahmadian and Dabidi Roshan, 2015; Beer et al., 2017; Wang et al., 2018) were reported, but the response patterns of LF (*k* = 4) (Bartels Matthew et al., 2003; Davrath Linda et al., 2006; Rodrigues Jhennyfer et al., 2019; Kaltsatou et al., 2020), LFnu (*k* = 3) (Perini et al., 2000; Bartels Matthew et al., 2003; Alves Naiane Ferraz et al., 2011), and LF/HF (*k* = 5) (Perini et al., 2000; Bartels Matthew et al., 2003; Alves Naiane Ferraz et al., 2011; Rodrigues Jhennyfer et al., 2019; Kaltsatou et al., 2020) were inconsistent.

Moderating effects of demographic variables on phasic HRV responses were assessed by six studies. Vagally-mediated HRV reactivity was independent of age [i.e., RMSSD (Corrêa et al., 2013), HFnu (Bartels Matthew et al., 2003; Kaltsatou et al., 2020), SD1 (Corrêa et al., 2013; Kaltsatou et al., 2020)], gender (i.e., RMSSD (Corrêa et al., 2013), HFnu (Perini et al., 2000; Bartels Matthew et al., 2003; Takahashi et al., 2003), SD1 (Corrêa et al., 2013)], and body mass index [i.e., Hfnu (Bartels Matthew et al., 2003)]. The reactivity of HRV_mixed_ was also mostly independent of age [i.e., VLF (Kaltsatou et al., 2020), LFnu (Bartels Matthew et al., 2003), LF/HF (Bartels Matthew et al., 2003), SD2 (Corrêa et al., 2013; Kaltsatou et al., 2020), DFA-α1 (Kaltsatou et al., 2020), CoV (Kaltsatou et al., 2020)], gender [i.e., mRR (Corrêa et al., 2013), LFnu (Perini et al., 2000; Bartels Matthew et al., 2003), LF/HF (Perini et al., 2000; Bartels Matthew et al., 2003), SD2 (Corrêa et al., 2013)], body mass index [i.e., LFnu (Bartels Matthew et al., 2003), LF/HF (Bartels Matthew et al., 2003)], and physical fitness (i.e., DFA-α1) (Karavirta et al., 2009). In case of suppressed HRV_mixed_ at higher ages [i.e., LF (Kaltsatou et al., 2020), LF/HF (Kaltsatou et al., 2020), DFA-α1 (Karavirta et al., 2009; Kaltsatou et al., 2020)], these effects vanished when controlling for body fat in the study of Kaltsatou et al. (2020).

Five studies found moderating effects of exercise intensity on phasic HRV responses: Higher intensities and higher metabolic demands of exercise (measured as % VO_2, peak_) were related to larger responses of vagally-mediated HRV [i.e., decrease in HF (*k* = 2) (Mayumi et al., 2008; Archiza et al., 2013), increase in HFnu (*k* = 1) (Perini et al., 2000)] and HRV_mixed_ [i.e., mRR (*k* = 1) (Virtanen et al., 2007), LFnu (*k* = 1) (Perini et al., 2000), LF/HF (*k* = 1) (Perini et al., 2000), and DFA-α1 (*k* = 1) (Karavirta et al., 2009)]. In contrast, two studies found no significant relation between exercise intensity and vagally-mediated HRV (Takahashi et al., 2003) or HRV_mixed_ (Wittstein et al., 2019).

#### Phasic HRV Responses to Resistance Exercises

Primarily vagally-mediated indices of HRV exhibited either a decrease [i.e., RMSSD (*k* = 1) (Machado-Vidotti et al., 2014), HFnu (*k* = 1) (Machado-Vidotti et al., 2014), HF (*k* = 2) (Piepoli et al., 1996; Machado-Vidotti et al., 2014), and SD1 (*k* = 1) (Machado-Vidotti et al., 2014)] or remained unchanged [i.e., RMSSD (*k* = 3) (Millar et al., 2011; Beer et al., 2017; Beer Noa et al., 2017), pNN50 (*k* = 1) (Millar et al., 2011)] compared to rest. HRV_mixed_ mainly decreased (*k* = 3) (Piepoli et al., 1996; Beer et al., 2017; Beer Noa et al., 2017) or remained unchanged (*k* = 3) (Piepoli et al., 1996; Petrofsky et al., 2009; Millar et al., 2011) compared to rest.

For resistance exercises, no moderating effects of demographic variables on phasic HRV responses were assessed. Associations between exercise demands and HRV revealed that HRV reactivities increased with increasing exercise intensities (Machado-Vidotti et al., 2014), while most HRV indices did not show a significant sensitivity to exercise duration (Millar et al., 2011). Additionally, HRV reactivity was more pronounced to upper- than lower limb exercise (Machado-Vidotti et al., 2014).

#### Phasic HRV Responses to Simultaneous Cognitive-Motor Training

Simultaneous execution of a physical (i.e., cycling at comfortable speed) and a cognitive task (i.e., mental arithmetic) induced significant reductions of vagally-mediated HRV [i.e., RMSSD (*k* = 2) (Beer et al., 2017; Beer Noa et al., 2017)]. HRV_mixed_ remained unchanged [i.e., SDNN (Beer et al., 2017)] or decreased [i.e., mRR (Beer et al., 2017), SDNN (Beer Noa et al., 2017)] in response to cognitive-motor training. None of the studies reported moderating parameters influencing phasic HRV responses during cognitive-motor training.

### Quantitative Synthesis

Eighteen studies met the inclusion criteria for the quantitative synthesis and were extracted for a synthesis of HRV reactivity in HOA (Table 3) and for a comparison of HRV between HOA and HA (Table 4).

**Table 3:**
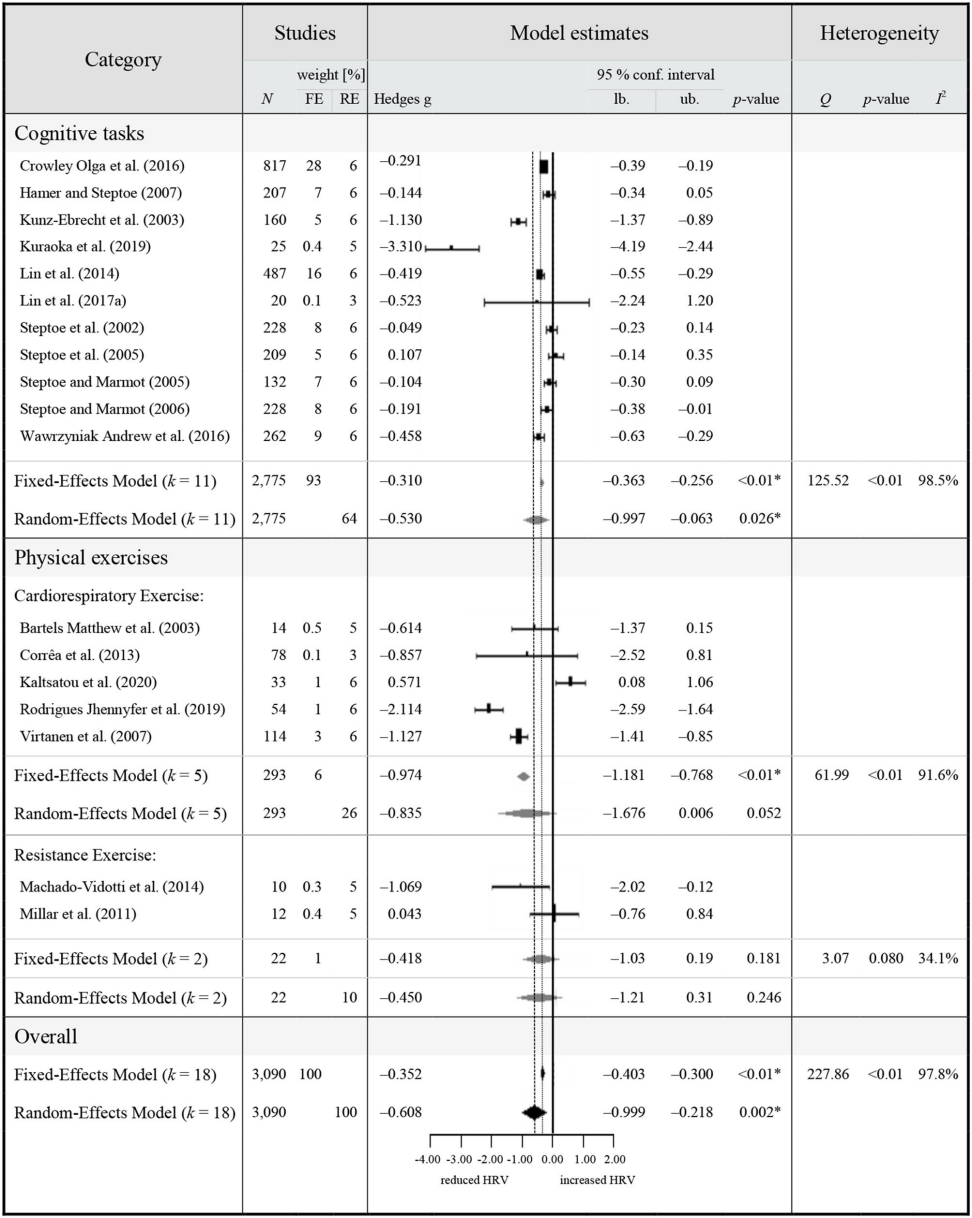
Meta-analytic results of HRV reactivity in healthy middle-aged to older adults (≥50 years).

*FE, Fixed-Effects Model; RE, Random-Effects Model; lb., lower bound; ub., upper bound.*

*Significant at p < 0.05.

**Table 4:** Meta-analytic results of HRV on-task in healthy middle-aged to older (≥50 years) adults compared to younger adults.

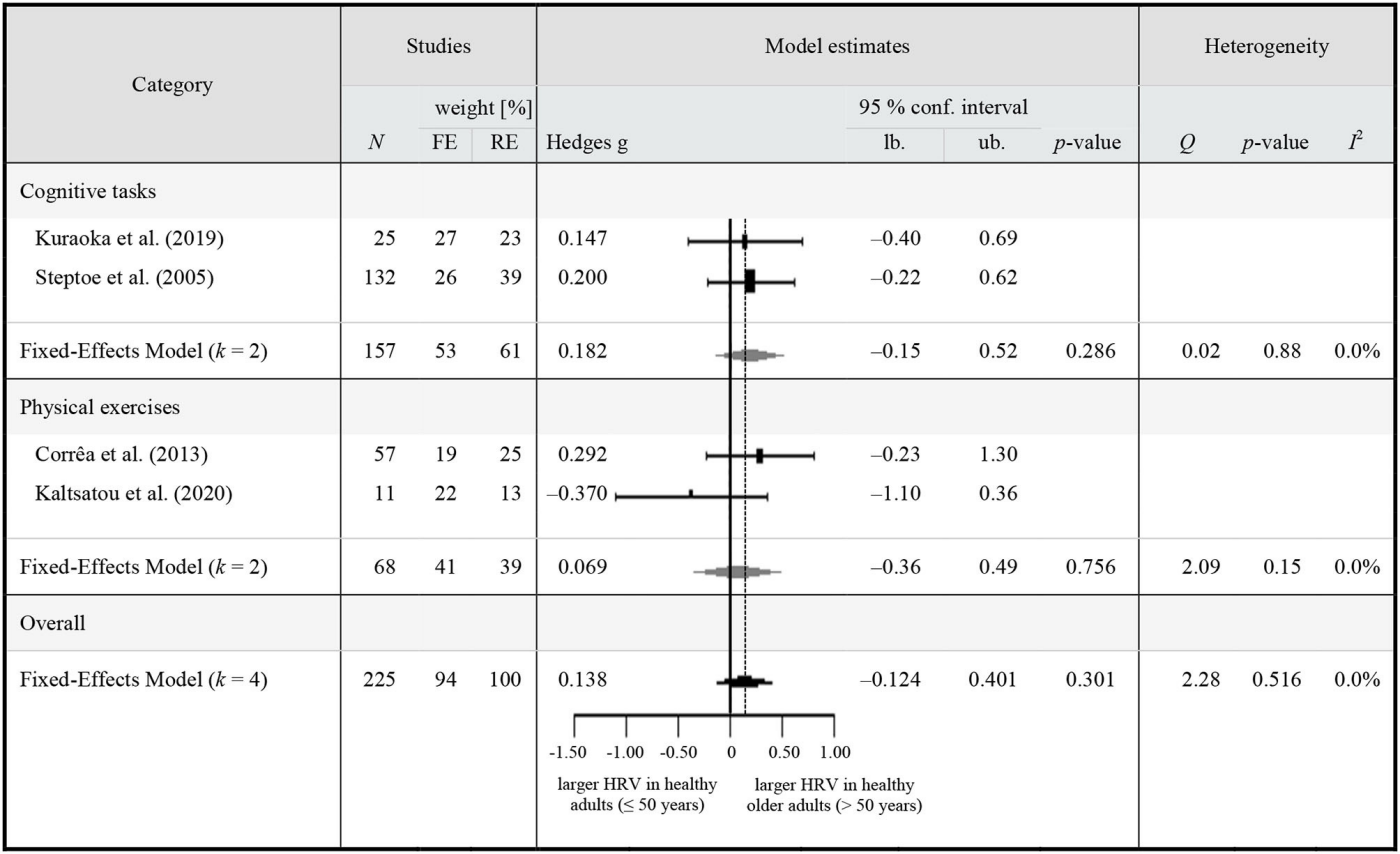

*FE, Fixed-Effects Model; RE, Random-Effects Model; lb., lower bound; ub., upper bound.*

*Significant at p < 0.05.

#### Meta-Analysis 1: HRV Reactivity During Cognitive and Physical Exercise Interventions

The overall pooled estimate revealed a significant reduction of vagally-mediated HRV during study interventions in HOA (Hedge's g = −0.352, 95% CI [−0.403 to −0.300], *p* < 0.001, *I*^2^ = 97.84%, *k* = 18, *N* = 3,090), indicating a significant parasympathetic withdrawal compared to rest. Due to the significant heterogeneity across studies (*Q* = 227.86, *p* < 0.01), a random-effects model was adopted. The random effects model also retrieved significant reductions of vagally-mediated HRV during study interventions in HOA, with a slightly larger magnitude (Hedge's g = −0.608, 95% CI [−0.999 to −0.218], *p* = 0.002, *I*^2^ = 97.84%, *k* = 18, *N* = 3,090) compared to the fixed-effects model. The level of true heterogeneity was large (*I*^2^ = 97.84%), indicating that <3% of heterogeneity was attributable to sampling error. Visual inspection of funnel plots as well as Egger's test revealed no significant funnel plot asymmetry (*z* = −1.359, *p* = 0.174).

The planned sub-group analyses revealed significant reductions of vagally-mediated HRV during cognitive (Hedge's g = −0.530, 95% CI [−0.997 to −0.063], *p* = 0.026, *I*^2^ = 98.5%, *k* = 11, *N* = 2,775) as well as physical exercises (Hedge's g = −0.742, 95% CI [−1.407 to −0.078], *p* < 0.029, *I*^2^ = 88.23%, *k* = 7, *N* = 315) in HOA. For the physical exercises, vagally-mediated HRV reactivity (i.e., decrease in vagally-mediated HRV compared to rest) was significant during cardiorespiratory exercises (Hedge's g = −0.835, 95% CI [−1.676 to −0.006], *p* = 0.006, *I*^2^ = 91.6%, *k* = 5, *N* = 293), but not during resistance exercises (Hedge's g = −0.450, 95% CI [−1.211 to 0.310], *p* = 0.246, *I*^2^ = 34.1%, *k* = 2, *N* = 22). None of the between-group differences in HRV reactivity were significant. Visual inspections and Egger's test indicated a significant funnel plot asymmetry among HRV reactivity during cognitive exercises (*z* = −2.437, *p* = 0.015). Using trim and fill did not affect the estimated effect size (Hedge's g = −0.5299, 95% CI [−0.9970 to −0.0628], *p* = 0.0262). No asymmetries of HRV reactivity during cardiorespiratory- (*z* = 0.161, *p* = 0.873) and resistance exercises were reported.

#### Meta-Analysis 2: Effects of Age on HRV During the Task

The pooled estimate revealed no significant difference in vagally-mediated HRV in HOA (*n* = 225) compared to HA (*n* = 94) during study interventions (*k* = 4; Hedge's g = 0.138, 95% CI [−0.124 to 0.401], *p* = 0.301, *I*^2^ = 0.0%, *k* = 4, *N* = 319). There was no significant heterogeneity across studies (*Q* = 2.28, *p* = 0.516). Visual inspection of funnel plots as well as Egger's test revealed no significant funnel plot asymmetry (*z* = −1.228, *p* = 0.220).

The planned sub-group analyses revealed no significant difference in vagally-mediated HRV between HOA (*n* = 157) compared to HA (*n* = 53) during cognitive exercises (Hedge's g = 0.182, 95% CI [−0.15 to 0.52], *p* = 0.286, *I*^2^ = 0.0%, *k* = 2, *N* = 210). During physical exercises, there was also no significant difference in vagally-mediated HRV between HOA (*n* = 68) and HA (*n* = 41) (Hedge's g = 0.069, 95% CI [−0.356 to 0.493], *p* = 0.756, *I*^2^ = 0.00%, *k* = 2, *N* = 109). The between-group difference was not significant (Δ Hedge's g = 0.113, SE difference = 0.275, *z* = 0.410, *p* = 0.682, *Q*_within_ = 2.11, *Q*_between_ = 0.168).

## Discussion

The aim of this systematic review was: (a) to summarize relevant literature monitoring phasic HRV responses of HOA to: (1) cognitive exercises, (2) physical exercises; and (3) simultaneous cognitive-motor training, and (b) to evaluate key moderating parameters influencing phasic HRV responses during these modes of exercises. The results suggest three main findings: First, vagally-mediated HRV indices are reduced during exercise indicating a significant parasympathetic withdrawal compared to resting state. Second, for cognitive exercises, associations between HRV reactivity and participant characteristics (i.e., age, cognitive functioning, and physical fitness), task demands (i.e., task complexity and -modality) and the individual responses to these cognitive challenges were found. Third, the reduction of vagally-mediated HRV during physical exercises is mainly moderated by exercise intensity. The quality of evidence for these findings is limited, however, since the large majority of identified studies applied observational designs, which do not allow analyzing causal relationships.

### Phasic HRV Responses to Cognitive Exercises

Cognitive exercises were accompanied by a moderate but significant reduction of vagally-mediated HRV compared to rest in HOA. This result is consistent with findings of previous systematic reviews and meta-analyses in HOA (Ranchet et al., 2017) and HA (Castaldo et al., 2015; Hughes et al., 2019) indicating a sensitivity of parasympathetic modulation to conditions with increased cognitive demands.

Several factors were identified to influence the pattern of phasic HRV responses during cognitive exercises. On-task values of vagally-mediated HRV did not differ between HOA and HA (i.e., meta-analysis 2) (Kuraoka et al., 2019) (i.e., study with moderate methodological quality) and (Steptoe et al., 2005) (i.e., study with high methodological quality), but HRV reactivity was reported to be blunted at higher age within the population of HOA (Crowley Olga et al., 2016) (i.e., study with a high methodological quality) or when compared to HA (Steptoe et al., 2005) (i.e., study with a high methodological quality). The reactivity of vagally-mediated HRV in HOA to cognitive exercises was independent of gender [i.e., RMSSD (Kunz-Ebrecht et al., 2003; Steptoe and Marmot, 2005) (i.e., both studies with a moderate methodological quality)] and other lifestyle factors [i.e., smoking, alcohol consumption and the level of physical activity; RMSSD (Steptoe et al., 2002) (i.e., study with moderate methodological quality)], but lower reactivities of vagally-mediated HRV were associated with increased physical fitness levels [i.e., RMSSD (Hamer and Steptoe, 2007) (i.e., study with high methodological quality)]. At the same time, higher task demands (i.e., task difficulty or complexity) and more pronounced individual responses to these demands were related to larger withdrawals of vagally-mediated HRV [i.e., RMSSD (Kunz-Ebrecht et al., 2003) (i.e., study with moderate methodological quality)] and [Wawrzyniak Andrew et al., 2016) (i.e., study with high methodological quality)]. Larger HRV reactivities predicted worse global cognition [i.e., HF (Lin et al., 2017a) (i.e., study with moderate methodological quality)] and processing speed [i.e., RMSSD (Wawrzyniak Andrew et al., 2016) (i.e., study with high methodological quality)], but not executive functioning [i.e., HF (Lin et al., 2014) [i.e., study with high methodological quality) and (Lin et al., 2017a) (i.e., study with moderate methodological quality)].

These observations are consistent with multiple studies supporting the predictions of the vagal tank theory (Laborde et al., 2018) by showing that a higher parasympathetic withdrawal during the cognitive exercises was associated with a higher cognitive load and worse cognitive performance (Mukherjee et al., 2011; Suriya-Prakash et al., 2015; Ranchet et al., 2017). It is well-documented that HRV decreases with increasing task complexity, mental effort and sustained attention required by a cognitive exercise in healthy adults as well as in the elderly population (Mukherjee et al., 2011; Luque-Casado et al., 2015; Hughes et al., 2019; Hillmert et al., 2020). According to Silvestrini (2017), the CAN (in particular the dorsal ACC) determines proportional adjustments of the cardiovascular reactivity and executive functioning in situations when automatic cognitive processes are insufficient (Silvestrini, 2017). Consequently, subjects with limited cognitive abilities are required to invest a higher mental effort to perform a task (Ranchet et al., 2017) which, in turn, can be measured by a larger reactivity of vagally-mediated HRV. Lin et al. (2017a) were able to corroborate these assumptions in older adults at risk for cognitive impairment and dementia by showing that ACC hyperactivity—that serves as a compensatory mechanism for neurodegeneration in frontal regions accompanied by a cognitive decline—mediates the relation to HF-HRV reactivities (Lin et al., 2017a). “As indicated in the literature, hyperactive ACC often comes from insufficient neural efficiency of frontal regions or the compensatory mechanism for neural loss of posterior regions” (Li et al., 2014; Lin et al., 2017a). This provides further support for a relationship between vagally-mediated HRV and the neural efficiency of the CAN (Thayer and Lane, 2000; Thayer, 2009; Lin et al., 2017a; Smith et al., 2017). However, these findings are mainly observational. This does not allow conclusions about the causal relationships between neural efficiency and the reactivity of vagally-mediated HRV in dependence of the specific cognitive exercises and sub-regions of the CAN.

### Phasic HRV Responses to Physical Exercises

Similar to cognitive exercises, there was a significant reduction of HRV during physical exercises. Consistent reductions of HRV during physical exercises have been summarized in multiple systematic reviews and were mainly investigated during cardiorespiratory exercises in HA and athletes (Dong, 2016; Michael et al., 2017; Vitale et al., 2019; Gronwald and Hoos, 2020).

In this systematic review, these vagally-mediated HRV responses were synthesized to be independent of age [(Bartels Matthew et al., 2003; Corrêa et al., 2013; Kaltsatou et al., 2020) (i.e., all studies with moderate methodological quality)], gender [(Bartels Matthew et al., 2003; Corrêa et al., 2013) (i.e., both studies with moderate methodological quality), (Perini et al., 2000) (i.e., study with high methodological quality), and (Takahashi et al., 2003) (i.e., study with low methodological quality)], and body mass index [(Bartels Matthew et al., 2003) (i.e., study with moderate methodological quality)] in HOA. The physical exercise intensity was the most often reported moderator variable, as shown by a positive relation between exercise intensity and the reactivities of vagally-mediated HRV [(Archiza et al., 2013) (i.e., study with moderate methodological quality), (Mayumi et al., 2008) (i.e., study with low methodological quality), and (Perini et al., 2000) (i.e., study with high methodological quality)] and HRV_mixed_ [(Perini et al., 2000; Karavirta et al., 2009) (i.e., both studies with high methodological quality) and (Virtanen et al., 2007) (i.e., study with moderate methodological quality)]. In contrast, two studies found no significant relation between exercise intensity and vagally-mediated HRV [(Takahashi et al., 2003) (i.e., study with low methodological quality)] or HRV_mixed_ [(Wittstein et al., 2019) (i.e., study with low methodological quality)].

This is in line with a comprehensive analysis of moderating variables of exercise dosages on HRV responses during exercise provided by Michael et al. (2017). Intensity was identified to be the strongest determinant of HRV responses during exercise. HRV shows a somewhat consistent dose-dependent response in form of a curvilinear decay as a function of exercise intensity and usually reaches its minimum at moderate exercise intensities. This pattern is closely related to the exercise heart rate (Michael et al., 2017). The findings of mostly unchanged (*k* = 3) (Millar et al., 2011; Beer et al., 2017; Beer Noa et al., 2017) or decreased (*k* = 2) (Piepoli et al., 1996; Machado-Vidotti et al., 2014) vagally-mediated HRV during resistance exercises might be explained by the low exercise intensities and predominantly static exercise modalities (Michael et al., 2017). Furthermore, a serious methodological issue arises when monitoring HRV during resistance exercises: Steady-state of exercise intensity needs to be warranted as a necessity for spectral HRV analysis (Aubert et al., 2003). This is often not possible at moderate to high resistance exercise intensities, which limits the applicability of HRV monitoring during resistance exercises.

### Phasic HRV Responses to Cognitive-Physical Exercise

Only two studies have analyzed HRV during simultaneous cognitive-motor training. Beer Noa et al. (2017) and Beer et al. (2017) applied a classic dual-task paradigm including the simultaneous performance of a mental arithmetic task and cycling at comfortable pace. Both studies reported a significant reduction of RMSSD compared to rest and no significant differences in SDNN during task performance. No significant differences in HRV were found compared to the isolated physical or cognitive exercises (Beer et al., 2017; Beer Noa et al., 2017) (i.e., both studies with low methodological quality). In line with the neurovisceral integration model (Thayer and Lane, 2000; Thayer, 2009; Smith et al., 2017), higher resting RMSSD were shown to be associated with better dual-task performance (Beer Noa et al., 2017) (i.e., study with low methodological quality).

To sum up, the body of evidence of cardiac vagal modulation during cognitive-motor training is strongly limited and rather in a fledgling state. Dual-task exercises are likely to reduce HRV while a higher resting cardiac vagal activity may predict better performance in cognitive-motor exercise, but further research is required to clarify these associations also taking into account the type of cognitive-motor training (i.e., sequential, simultaneous additional, and simultaneous incorporated cognitive-motor training (Herold et al., 2018)].

### Applications of HRV

Having evaluated the current evidence of HRV reactivity and its moderating variables, possible relevant applications using HRV as a biomarker and monitoring tool are discussed:

#### Phasic HRV Responses as a Biomarker to Monitor Internal Training Load

An optimal parameter for assessing internal training load in real-time should reflect the “actual psychophysiological response that the body initiates to cope with the requirements elicited by the external load” (Impellizzeri et al., 2019). According to the neurovisceral integration model (Thayer and Lane, 2000) and its advancements (Thayer, 2009; Smith et al., 2017), HRV is able to index the functional integrity of the CAN. The CAN regulates physiological, emotional and cognitive responses to environmental challenges (Thayer, 2009), which is central to understand an individual's adaptability to the situation (Laborde et al., 2018). Therefore, by monitoring HRV reactivity during cognitive or physical demands, the fundamental requirements to quantify internal training load are met.

During simultaneous cognitive-motor training, cognitive performance is mainly determined by external loads like the exercise duration, -frequency, -intensity, and task complexity (Lauenroth et al., 2016). Exercise intensity and task complexity were considered to be the main determinants to increase neuroplasticity and cognition (Netz, 2019). This systematic review has synthesized evidence indicating that vagally-mediated HRV is indeed sensitive to task demands (i.e., task difficulty, physical intensity) and the individual responses to these demands. The behavior of HRV at different intensities is largely coherent with heart rate during physical exercise. Heart rate was already recognized as a valid marker for the relative exercise intensity of cardiorespiratory exercises (Impellizzeri et al., 2019). Additionally, HRV is increasingly seen as a promising marker for exercise prescription and monitoring of the internal training load during cardiorespiratory exercises (Dong, 2016; Singh et al., 2018b; Gronwald and Hoos, 2020; Gronwald et al., 2020). For cognitive exercises, it was proposed, that the cardiovascular reactivity is proportionally adjusted to the intensity of controlled cognitive processes (e.g., task difficulty) (Silvestrini, 2017). Although there are already investigations showing that multiple HRV parameters are sensitive and reliable to quantify mental effort during cognitive exercises (Mukherjee et al., 2011), the relation of HRV reactivity and cognition differs between cognitive task domains (Lin et al., 2014, 2017a; Wawrzyniak Andrew et al., 2016). Nonetheless, HRV shows promising characteristics as a marker for internal training load during physical- and cognitive exercises. HRV reactivity during simultaneous cognitive-motor training in HOA is not thoroughly investigated, yet. Therefore, the implementation of HRV to measure the internal training load during simultaneous cognitive-motor training is currently not applicable and requires further investigations. Such research is warranted because of the rising incidence of the motoric cognitive risk syndrome in aging societies (Verghese et al., 2014) that calls for the development of preventive interventions that consider both motoric as well as cognitive training elements (Herold et al., 2018).

#### Phasic HRV Responses as a Biomarker to Guide Training Interventions

Indications of a relationship between vagally-mediated HRV and the neural efficiency of the CAN have been reported (Thayer and Lane, 2000; Thayer, 2009; Lin et al., 2017a; Smith et al., 2017). However, in this systematic review only three interventional studies measuring HRV reactivity before and after a training intervention were identified. Such an “intervention approach may provide a viable pathway to determine the causal relationship between neural efficiency and […] HRV” (Lin et al., 2017a). Karavirta et al. (2009) examined “the effects of combining endurance and strength training compared with endurance or strength training alone on HR dynamics and physical fitness in older previously untrained men aged 40–67 years” (Karavirta et al., 2009): HF increased at exercise intensities of 90–130 W in all groups except in the isolated strength-training group. The fractal HR behavior (i.e., DFA-α1) improved after a 21-week intervention period, but only in the combined endurance and strength-training group. The training-related HRV changes only correlated with the changes of resting HR (*r* = 0.49, *p* = 0.009) and the initial level of DFA-α1 (*r* = −0.48, *p* = 0.009) for DFA-α1 at rest, and with age (*r* = −0.44, *p* = 0.023) for DFA-α1 at near-maximal exercise. More importantly, no significant correlations were found between “the changes in VO_2, max_ and the changes in DFA-α1 at rest or at any relative exercise intensity” (Karavirta et al., 2009). The studies of Junior Adalberto et al. (2019) and Rodrigues Jhennyfer et al. (2019) applied a walking training with and without blood-flow restriction for 6 weeks and a combined strength- and endurance training intervention for 12 weeks comparing different periodization models, respectively. Unfortunately, they did not analyze the relations between training-related changes and changes in HRV at rest or on-task (Rodrigues Jhennyfer et al., 2019).

Fortunately, there are other reports which offer some more preliminary findings in older adults at risk for cognitive decline or dementia. The intervention studies of Lin et al. (2017b, 2020) reported, that cognitive training strengthened the efficiency of the striatum-prefrontal connectivity, and that there was a “consistent link between HF-HRV and training-induced improvements” (Lin et al., 2017b) showing that “changes in task-related HF-HRV but not resting HF-HRV were also related to the cognitive and neural changes in response to intervention” (Lin et al., 2017b, 2020). This may be viable in the observation of neurobiological effects of training interventions aiming to improve cognition in populations at risk for cognitive decline, since “strengthening pathways regulating PNS may be efficacious for maintaining functional health in later life” (Lin et al., 2017a). With that, monitoring HRV reactivity may be considered as a promising and readily applicable approach to study dose-response relationships of training interventions aiming to improve brain structure and function. This approach would facilitate the process of investigating and identifying the optimal intervention characteristics to maximize exercise effectiveness.

To the best of our knowledge, there have only been only studies using this approach for resting HRV. For example, a recent meta-analysis by Raffin et al. (2019) has identified physical exercise frequency to be a key factor in promoting increases in cardiac vagal control at rest in HOA (Raffin et al., 2019). Since higher resting HRV was shown to predict better cognitive functioning (Thayer and Lane, 2000; Thayer, 2009; Lin et al., 2014; Crowley Olga et al., 2016; Wawrzyniak Andrew et al., 2016; Beer Noa et al., 2017; Smith et al., 2017; Forte et al., 2019), higher exercise frequencies might be a central element to be considered in the design of exercise interventions. Regarding the type of training intervention, a recent randomized controlled trial of Eggenberger et al. (2020) has shown, that a cognitive-motor exergame training was able to improve vagally-mediated HRV at rest, whereas a dual-task training paradigm and an exclusively physical training intervention did not affect HRV. Moreover, cognitive executive functioning (i.e., measured by the Trail-Making-Test (TMT)-B) was the most prominent significant predictor of variance in vagally-mediated HRV (i.e., RMSSD, HF; Eggenberger et al., 2020). This is consistent with the findings, that cognitively engaging exercises (i.e., simultaneous cognitive-motor training) appear to have the strongest effect on cognition in HOA (Diamond and Ling, 2016; Biazus-Sehn et al., 2020; Chen et al., 2020) and older adults at risk for cognitive impairment and Dementia (Wu et al., 2019; Biazus-Sehn et al., 2020). Moreover, HRV might also be useful to personalize training programs. Training prescription guided by resting HRV was already shown to enhance training effects of endurance training in younger adults (Düking et al., 2020; Granero Gallegos et al., 2020; Ruiz et al., 2020).

Nonetheless, absolute values of resting HRV often show large interindividual variations that are additionally dependent on factors like measurement methodology, controlling of confounding variables, and the complex interactions influencing HRV (Nunan et al., 2010; Laborde et al., 2017). Comparing HRV reactivities to controlled task scenarios (e.g., as part of a cognitive assessment) in addition to resting HRV might, therefore, allow more consistent observations. Therefore, future investigations should try to establish the causal relation between HRV and training-related adaptations on brain structure and function while applying recommendations for experiment planning, data analysis, and data reporting (Laborde et al., 2017) and considering tonic (i.e., resting) HRV as well as phasic HRV responses (i.e., HRV reactivity and possibly also -recovery) (Laborde et al., 2018) in within-subjects experimental designs (Laborde et al., 2017).

#### Phasic HRV Responses as a Biomarker to Predict Cognitive Decline

An early identification of cognitive impairment is important to prevent further deteriorations (Morley et al., 2015). In subjects with amnestic mild cognitive impairment (aMCI), a significant faster decline in memory and executive functioning is observed compared to HOA. The deteriorations in memory and executive functioning, accompanied by changes in various brain structures (including the hippocampus and the cingulate cortex), are considered critical to distinguish aMCI from healthy aging (Johnson et al., 2012; Janelidze and Botchorishvili, 2018; Chehrehnegar et al., 2020). Under the assumption of the neurovisceral integration model (Thayer and Lane, 2000; Thayer, 2009; Smith et al., 2017) and the vagal tank theory (Laborde et al., 2018), these changes might affect the cardiac vagal activity at rest and in response to cognitive demands. In fact, parasympathetic dysfunctions have been shown to be prevalent in aMCI and may likely be caused by neuroanatomical changes in the CAN (Collins et al., 2012). Hence, HRV can be considered a promising early biomarker of cognitive deteriorations (Collins et al., 2012). A systematic review of da Silva et al. (2018) identified blunted resting HRV for almost all indices in dementia patients compared to HOA. Due to the small effect sizes and the large heterogeneity, they concluded that more research is needed to clarify the value of HRV as a biomarker for cognitive impairments (da Silva et al., 2018). Phasic HRV measurement may be more promising: A systematic review by Ranchet et al. (2017) indicated the sensitivity for detecting changes in cognitive workload of a variety of physiological measures [i.e., electroencephalography, magnetoencephalography, functional magnetic resonance imaging, positron emission tomography, measures from eye tracking or/and pupillometry (e.g., pupil size) and cardiovascular measures (e.g., blood pressure)]. They concluded that “physiological measures could detect early cognitive symptoms in older adults, even prior to the presence of cognitive deficits in behavioral performances” (Ranchet et al., 2017). Unfortunately, they only identified one study measuring HRV (Ranchet et al., 2017). In this review identified only one study involving cognitively impaired populations (MoCA score ≤ 26) reporting vagally-mediated HRV was identified. The study of Lin et al. (2017a) applied a computerized Stroop Color Word task, which requires executive functioning. In such a scenario, the “vagal tank theory” (Laborde et al., 2018) would predict larger vagal-withdrawals to be maladaptive. Corroborating the predictions of the vagal tank theory regarding HRV reactivity but not resting HRV, the study reported higher resting HF-HRV at rest (*r* = −0.38, *p* < 0.024) and larger HRV reactivities during cognitive exercises (*r* = 0.39, *p* < 0.022) for aMCI patients with more severe neurodegeneration. Both results were mediated by an anterior cingulate cortex hyperactivity (*r* = 0.70, *p* < 0.001; *r* = −0.74, *p* < 0.001). Importantly, these relationships were unaffected by the clinical phenotype (i.e., HOA vs. aMCI; Lin et al., 2017a).

To summarize, HRV reactivity measured to cognitively engaging exercises might offer worthwhile features in the early detection of cognitive impairment. More research is warranted to conclude about its value as a predictive biomarker or as a biomarker used to guide cognitive-motor training (e.g., exergaming) interventions.

### Limitations

The outcomes of this systematic review with meta-analyses have to be interpreted with some caution considering the following limitations: **First**, a large variety of study designs (i.e., controlled clinical trials and observational studies), measurement methods and study interventions were included, which resulted in a significant heterogeneity in meta-analysis one. **Second**, multiple studies controlled for covariates (i.e., age, gender, and education) in their statistical analysis, which were not reported in detail. This might have had an influence on the conclusion of this systematic review, since possible moderating effects of these variable could not have been analyzed. **Third**, the analysis for controlling confounders revealed that a substantial number of studies controlled for <50% of selected confounders that could influence HRV. Consequently, these confounders may have influenced the results of the corresponding studies that, in turn, might have distorted the findings of this systematic review. **Fourth**, for the meta-analyses, multiple parameters of HRV were merged into a group of vagally-mediated HRV indices. This was performed on basis of a hierarchical inclusion of HRV indices mainly reflecting cardiac vagal tone: (1) RMSSD, (2) pNN50, (3) HFnu, (4) HF, and (5) SD1 (Electrophysiology TFotESoCtNASoP, 1996; Alvares et al., 2016; Ernst, 2017; Laborde et al., 2017; Shaffer and Ginsberg, 2017; Mika et al., 2020). Future studies should, if possible, analyze each parameter separately to identify differences in their sensitivity during cognitive or physical exercises.

## Conclusion

This systematic review with meta-analyses showed that vagally-mediated HRV is significantly reduced during cognitive and/or physical exercises compared to resting state, indicating a significant parasympathetic withdrawal compared to rest. The key moderating variables of these responses identified included exercise intensity for physical exercises, and participant characteristics (i.e., level of cognitive functioning, physical fitness), task demands (i.e., task complexity and modality) and the individual responses to these cognitive challenges for cognitive exercises. In particular, higher task demands (task complexity, physical intensity) were related to larger HRV reactivities. Better physical fitness and cognition were associated with lower HRV reactivities. Additionally, HRV reactivity appeared to be sensitive to training-induced cognitive and neural changes. HRV reactivity seems to be a promising biomarker for monitoring internal training load and evaluating neurobiological effects of training interventions. Further research is warranted to evaluate the potential of HRV reactivity as a monitoring parameter to guide cognitive-motor training interventions and/or as a biomarker for cognitive impairments. This may facilitate the early detection of cognitive impairment as well as allow individualized training adaptations that, in turn, support the healthy aging process by optimizing individual exercise dose and progression of cognitive-motor training.

## Data Availability Statement

The original contributions presented in the study are included in the article/Supplementary Material, further inquiries can be directed to the corresponding author/s.

## Author Contributions

PM, RK, and EB were responsible for the conception and protocol development of the review. PM and MT were responsible for the literature research and writing of the manuscript. EB supervised the selection of the studies. EB, RK, MT, and MA contributed to the revision of the manuscript. All authors revised, read, and approved the submitted version.

## Conflict of Interest

The authors declare that the research was conducted in the absence of any commercial or financial relationships that could be construed as a potential conflict of interest.

## Publisher's Note

All claims expressed in this article are solely those of the authors and do not necessarily represent those of their affiliated organizations, or those of the publisher, the editors and the reviewers. Any product that may be evaluated in this article, or claim that may be made by its manufacturer, is not guaranteed or endorsed by the publisher.
