# Supplementary material for: Can Reactivity of Heart Rate Variability Be a Potential Biomarker and Monitoring Tool to Promote Healthy Aging? A Systematic Review With Meta-Analyses
Source: Front Physiol. 2021 Jul 29;12:686129. doi: 10.3389/fphys.2021.686129 (PMC8359814; doi:10.3389/fphys.2021.686129)
Supplement: Supplementary file 1 [file Data_Sheet_3.docx]

Appendix

## Appendix A: Study Characteristics

| Table A1: Overview of the Study Characteristics | | | | | | | | | | | | | | | | | | | | | |
| --- | --- | --- | --- | --- | --- | --- | --- | --- | --- | --- | --- | --- | --- | --- | --- | --- | --- | --- | --- | --- | --- |
| Reference | | | Participants | | | | Intervention(s) | | Measurements | | Controlled Confounders | | | | | | | | | | |
|  |  |  |  |  |  |  |  |  |  |  | Stable Variables | | | | | Transient Variables | | | | | |
| Author | Year | Study Type ^(1)^ | Group ^(2)^ | N | Females | mean Age | Exercise Characteristics | Rest ^(3)^  (a), (b)  (time) | Type ^(4)^ | Device | Age and gender | Smoking | Alcohol consumption | Body constitution | Medication | Sleep routine | Physical activity | Food consumption | Caffein intake | Bladder emptying | Alcohol intake |
| Ahmadian et al. ([150](#_ENREF_150)) | 2015 | CC | HOA | 12 | 0 % | 51.5 | Two continuous incremental exercise protocols on (1) arm crank ergometer (start level = 25 W) and (2) cycle ergometer (start level = 50 W); with increases of 25 W/2 min and a cadence of 50 rpm | S, NR (1 min) | ECG | Custo Cardio 100 (Custo Med Gmbh, Germany ) | x | ✓ | x | x | ✓ | ✓ | ✓ | x | ✓ | x | x |
|  |  |  | HA | 12 | 0 % | 34.1 |  |  |  |  |  |  |  |  |  |  |  |  |  |  |  |
| Alves et al. ([151](#_ENREF_151)) | 2011 | CC | HOA | 8 | 100 % | 55.6 | Constant load exercise on cycle ergometer at moderate physical intensity for 40 min | L, NR (10 min) | ECG | Stemtech, Inc.,  GPA-4, model 2 | ✓ | x | x | ✓ | x | ✓ | x | ✓ | ✓ | x | ✓ |
|  |  |  | D_hypertension_ | 9 | 100 % | 54.9 |  |  |  |  |  |  |  |  |  |  |  |  |  |  |  |
|  |  |  | D_hypertension_ | 8 | 100 % | 55.6 |  |  |  |  |  |  |  |  |  |  |  |  |  |  |  |
| Archiza et al. ([152](#_ENREF_152)) | 2013 | ITS | HOA | 25 | 0 % | 66 | Inspiratory resistive load exercise, seated, at 12 breaths per minute for 4 x 4 minutes at 30 %, 60 % and 80% of max. inspiratory load in randomized order | S; SB (5 min) | PPG | Polar S810i | ✓ | ✓ | x | ✓ | ✓ | ✓ | ✓ | x | ✓ | x | x |
| Bartels et al. ([153](#_ENREF_153)) | 2003 | CC | HOA | 14 | 50 % | 60 | Ramp test (start: 0 W, increments of 5 / 10 W/min (depending on max. voluntary ventilation (MVV) < 40 L/min / ≥ 40 L/min) on bicycle ergometer until max. volitional exercise capacity | S; SB (5 min) | ECG | Marquette  Max-1 | ✓ | x | x | ✓ | x | x | x | ✓ | ✓ | x | ✓ |
|  |  |  | D_COPD_ | 53 | 49 % | 63 |  |  |  |  |  |  |  |  |  |  |  |  |  |  |  |
| Beer et al. ([154](#_ENREF_154)) | 2017a | CC | HOA | 15 | 40 % | 63 | (1) 2 min static muscle contraction at 30 % max. voluntary pressure (MVP), (2) mental arithmetic task for 1 min, (3) 1 min mental arithmetic while cycling at comfortable pace | S; NR (10 min) | ECG | Polar RS800CX | ✓ | x | x | x | x | x | x | x | x | x | x |
|  |  |  | D_stroke_ | 13 | 23 % | 60 |  |  |  |  |  |  |  |  |  |  |  |  |  |  |  |
| Beer et al. ([155](#_ENREF_155)) | 2017b | CC | HOA | 16 | 38 % | 63 | (1) 2 min static muscle contraction at 30 % MVP, (2) mental arithmetic task for 1 min, (3) 2 min cycling at comfortable pace, (4) 1 min mental arithmetic while cycling at comfortable pace | S, NR (10 min) | ECG | Polar RS800CX | ✓ | x | x | x | x | x | x | x | x | x | x |
|  |  |  | D_stroke_ | 19 | 37 % | 63.5 |  |  |  |  |  |  |  |  |  |  |  |  |  |  |  |
| Betz et al. ([156](#_ENREF_156)) | 2017 | CC | HOA | 19 | 47 % | 50.7 | Computerized mental arithmetic task for 5 min (45 tasks) | S, NR (5 min) | ECG | BrainVision (Brain Products GmbH, Gilching, Germany) | ✓ | ✓ | ✓ | x | ✓ | x | x | x | x | x | x |
|  |  |  | D_tinitus_ | 19 | 37 % | 47.9 |  |  |  |  |  |  |  |  |  |  |  |  |  |  |  |
| Cacioppo et  al. ([157](#_ENREF_157)) | 2000 | CC | HOA | 37 | 100 % | 67.2 | Mental arithmetic task 6 x 1 min | S, NR (6 min) | ECG | Minnesota  Model 304B | ✓ | x | ✓ | ✓ | x | x | ✓ | ✓ | ✓ | x | ✓ |
|  |  |  | D_Caregivers_ | 27 |  |  |  |  |  |  |  |  |  |  |  |  |  |  |  |  |  |
| Capuana et al. ([158](#_ENREF_158)) | 2012 | CC | HOA | 18 | 61 % | 72.3 | Working memory inhibitory control (WMIC) task, 18 runs (456 Go and 150 NoGo trials at each level of working memory load) | S, NR (5 min) | ECG | BioSemi | x | x | x | x | x | x | x | x | x | x | x |
|  |  |  | HA | 22 | 77 % | 20.5 |  |  |  |  |  |  |  |  |  |  |  |  |  |  |  |
| Christensen et al. ([159](#_ENREF_159)) | 2014 | CC | HOA | 19 | 53 % | 57.1 | Three verbal (i.e. letters) and three spatial (i.e. circles) n-back tasks that varied in processing load: one-back, two-back, and three-back, 33 targets per block, each containing 100 stimuli | S, NR (5 min) | ECG | BIOPAC Student Labs (BSL) PRO MP35 | x | x | x | x | ✓ | x | x | x | ✓ | x | x |
|  |  |  | D_aphasia_ | 8 | 38 % | 54.6 |  |  |  |  |  |  |  |  |  |  |  |  |  |  |  |
| Collste et al. ([160](#_ENREF_160)) | 2014 | CC | HOA | 22 | NR | 63.6 | Mental stress task with two parts: (1) anger recall interview, (2) mental arithmetics task (subtract 7 from 200 in continuing steps) | NR, NR (10 min) | ECG | Novacor Vista Plus (Ruell, Malmaison, France) Holter-ECG | ✓ | x | x | x | x | x | x | x | x | x | x |
|  |  |  | D_cardiomyopathy_ | 22 | NR | 63.2 |  |  |  |  |  |  |  |  |  |  |  |  |  |  |  |
| Corrêa et al. ([161](#_ENREF_161)) | 2013 | COH | HOA | 78 | 54 % | NR | 6-minute walk test performed in a 30-m hallway (6MWT) | NR, NR (1 min) | PPG | Polar RS800 | ✓ | ✓ | ✓ | ✓ | ✓ | x | x | x | ✓ | x | ✓ |
| Crowley et al. ([162](#_ENREF_162)) | 2016 | COH | HOA | 817 | 56 % | 57.1 | Computerized mental arithmetic task and the Stroop color-word conflict task | S, NR  (11 min) | ECG | National  Instruments  A/D board | ✓ | ✓ | x | ✓ | ✓ | x | x | x | ✓ | x | x |
| Davrath et al. ([163](#_ENREF_163)) | 2006 | CC | HOA | 8 | 0 % | 52 | 90 % age-predicted HR_max_ exercise treadmill test using the standard Bruce protocol | S, NR  (10 min) | ECG | MP100-BIOPAC  system | x | x | x | ✓ | ✓ | x | x | x | x | x | x |
|  |  |  | HOA | 20 | 20 % | 56 |  |  |  |  |  |  |  |  |  |  |  |  |  |  |  |
| Dourado et al. ([164](#_ENREF_164)) | 2010 | COH | HOA | 10 | 30 % | 56 | Incremental shuttle walk test consisting of walking a 10-m course delimited by 2 traffic cones at a progressive pace with increments of 0.17 m/s every minute. | NR, NR  (NR) | PPG | Polar S810i | ✓ | ✓ | ✓ | ✓ | ✓ | x | x | x | ✓ | x | ✓ |
| Hamer &  Steptoe ([165](#_ENREF_165)) | 2007 | COH | HOA | 207 | 46 % | 52.0 | 5 min of computerized Stroop colour-word task and 5 min of mirror-tracing stress task | NR, NR (5 min) | ECG | VU-AMS | ✓ | ✓ | ✓ | ✓ | x | x | ✓ | x | ✓ | x | x |
| Junior et al. ([166](#_ENREF_166)) | 2019 | RCT | HOA | 21 | 0 % | 52.0 | 10 min constant load exercise on a treadmill at 60 % of individually determined maximal aerobic speed | NR, NR (NR) | PPG | Polar RS800 | ✓ | x | x | ✓ | ✓ | x | ✓ | x | ✓ | x | ✓ |
| Kaltsatou et al. ([167](#_ENREF_167)) | 2020 | COH | HOA | 11 | 0 % | 62.9 | 4 x 15-min (15 min rest) semi-recumbent cycling at constant rate of metabolic heat production (400 W) in a temperature-controlled, hot-dry environment (35 °C and 20 % relative humidity) | S, NR (5 min) | ECG | Holter monitor | x | ✓ | x | ✓ | ✓ | x | ✓ | ✓ | ✓ | x | ✓ |
|  |  |  | HA | 11 | 0 % | 43.5 |  |  |  |  |  |  |  |  |  |  |  |  |  |  |  |
|  |  |  | HA | 11 | 0 % | 25.8 |  |  |  |  |  |  |  |  |  |  |  |  |  |  |  |
| Karavirta et al. ([168](#_ENREF_168)) | 2009 | RCT | HOA | 93 | 0 % | 55.6 | Ramp test on bicycle ergometer at pedaling frequency of 60 rpm, warm up at light exercise intensity (50 W) for 5 min, start level of ramp test at 50 W, increments of 20 W/2 min until exhaustion | L, NR (5 min) | PPG | Polar S810i | ✓ | x | x | ✓ | ✓ | x | x | ✓ | x | x | x |
| Kunz-Ebrecht et al. ([169](#_ENREF_169)) | 2003 | COH | HOA | 232 | 47 % | 52.3 | Computerized Stroop Color Word and mirror-tracing stress task, each for 5 min in randomized order | S, NR (5 min) | ECG | VU-AMS | ✓ | x | ✓ | ✓ | x | x | x | x | ✓ | x | x |
| Kuraoka et al. ([170](#_ENREF_170)) | 2019 | COH | HOA | 25 | 0 % | 55.9 | Computerized mental arithmetic task and a mirror tracing task for 5 min each | S, NR (5 min) | ECG | Nihon Kohden Pocket ECG Monitor WEC-7101 | x | x | x | x | x | x | x | x | x | x | x |
|  |  |  | HA | 27 | 0 % | 32.4 |  |  |  |  |  |  |  |  |  |  |  |  |  |  |  |
| Lin et al. ([171](#_ENREF_171)) | 2014 | COH | HOA | 487 | 54 % | 65.1 | Mental arithmetic task and Stroop word-color task in randomized order, each for a duration of 6 min | S, NR (5 min) | ECG | NR | ✓ | ✓ | ✓ | x | x | x | x | x | x | x | x |
| Lin et al. ([172](#_ENREF_172)) | 2017 | CC | HOA | 20 | 60 % | 71.0 | Computerized Stroop Color Word and Dual 1-back task, each for 10 min in randomized order | NR, NR (10 min) | ECG | Biopac electrocardiagraphy | ✓ | x | x | x | x | x | x | x | ✓ | x | x |
|  |  |  | COG | 18 | 56 % | 74.4 |  |  |  |  |  |  |  |  |  |  |  |  |  |  |  |
| Machado- Vidotti et al. ([173](#_ENREF_173)) | 2014 | COH | HOA | 10 | 0 % | 65 | Incremental resistance exercise: 4 min incline (45°) bench and leg press, start level at 10 % of 1RM, stepwise increases in load until exhaustion, 12 reps per minute, controlled breathing pattern | S, NR (5 min) | PPG | Polar Vantage | ✓ | ✓ | x | ✓ | ✓ | x | ✓ | ✓ | ✓ | x | ✓ |
| Mayumi et al. ([174](#_ENREF_174)) | 2008 | CC | HOA | 32 | 0 % | 52.2 | Graded and symptom-limited exercise test on bicycle ergometer with a pedaling speed of 50 rotations per min for 2 min at each level of constant load (25 W 50 W, 75 W, 100 W, and 125 W) | NR, NR (2 min) | NR | NR | ✓ | x | x | x | x | x | x | x | x | x | x |
|  |  |  | D_hypertension_ | 37 | 0 % | 53.1 |  |  |  |  |  |  |  |  |  |  |  |  |  |  |  |
| Millar et al. ([175](#_ENREF_175)) | 2011 | CCT | HOA | 12 | 33 % | 70 | Isometric handgrip exercises in supine position at 30 % MVC: (1) 4 x 2 min, 1 min rest; (2) 8 x 1 min, 30 s rest; (3) 16 x 30 s, 15 s rest; (4) sham control trial: 4 x 2 min, 3 % MVC, 1 min rest | L, NR (15 min) | ECG | Powerlab (AD Instruments) | ✓ | ✓ | x | ✓ | ✓ | x | ✓ | x | ✓ | x | x |
| Norcliffe-Kaufmann et al. ([176](#_ENREF_176)) | 2016 | CC | HOA | 10 | 100 % | NR | Standardized Stroop color-word test for 5 minutes (Stroop effect app, version 1.1.0, Codeprincipals.com for Apple iPad) | L, SB (5 min) | ECG | PowerLab 16SP and LabChart 7; AD Instruments | ✓ | x | x | ✓ | ✓ | x | x | ✓ | ✓ | x | x |
|  |  |  | D_cardiomyopathy_ | 10 | 100 % | NR |  |  |  |  |  |  |  |  |  |  |  |  |  |  |  |
| Perini et al. ([177](#_ENREF_177)) | 2000 | COH | HOA | 23 | 48 % | 74.0 | Constant load exercise on cycle ergometer at a pedal frequency of 50 rpm for 5 min with wheel resistance of (1) 4.9 N and (2) 14.7 N for males and (1) 2.45 N and (2) 7.34 N for females | S/L, SB (10 min) | ECG | NR | ✓ | ✓ | x | ✓ | x | x | x | x | x | x | x |
| Perpetuini et al. ([178](#_ENREF_178)) | 2019 | CC | HOA | 15 | 33 % | 69.3 | Rey–Osterrieth complex figure task: (1) reproduce a complex two-dimensional image (copying); (2) 10 min break; (3) draw the complex two-dimensional image again from memory (recall) | NR, NR (5 min) | fITI | digital thermal infrared camera FLIR SC660 | x | x | x | x | x | x | x | x | x | x | x |
|  |  |  | COG | 10 | 40 % | 72.3 |  |  |  |  |  |  |  |  |  |  |  |  |  |  |  |
| Petrofsky et al. ([179](#_ENREF_179)) | 2009 | CC | HOA | 15 | 47 % | 65.8 | Isometric handgrip exercise in supine position: (1) 1 x 1 min at 10 % of MVC, 2 min rest; (2) 1 x 1 min at 25 % of MVC, 5 min rest; (3) 1 x at 40 % of MVC to fatigue (tension drop by ≤ 10 %) | S, NR (20 min) | ECG | NR | x | ✓ | x | ✓ | x | x | x | x | x | x | x |
|  |  |  | HA | 15 | 53 % | 30.6 |  |  |  |  |  |  |  |  |  |  |  |  |  |  |  |
|  |  |  | D_diabetes(type 2)_ | 15 | 47 % | 63.4 |  |  |  |  |  |  |  |  |  |  |  |  |  |  |  |
| Piepoli et al. ([180](#_ENREF_180)) | 1996 | CC | HOA | 10 | 10 % | 59.2 | Repetitive handgrip exercise in seated position at 50 % of MVC with 40 contractions per minute until exhaustion | L, NR (30 min) | ECG | NR | ✓ | x | x | x | x | x | ✓ | ✓ | x | x | x |
|  |  |  | D_heart failure_ | 12 | 25 % | 59.6 |  |  |  |  |  |  |  |  |  |  |  |  |  |  |  |
| Rodrigues et al. ([181](#_ENREF_181)) | 2019 | CCT | HOA | 54 | 100 % | 61.6 | Ramp exercise test on treadmill: 10 min warm up at fast walking speed, then incline of treadmill for 2°/min until voluntary exhaustion (approx. 8 – 12 minutes), maintaining fast walking speed | S, SP (10 min) | ECG | Polar Team Pro Sensor | ✓ | x | x | ✓ | x | x | x | x | ✓ | x | x |
| Sales et al. ([182](#_ENREF_182)) | 2011 | CC | HOA | 10 | 20 % | 50.8 | Incremental exercise test on cycle ergometer with 15 watts of initial workload and 15 watts increments at each 3-min stage until volitional exhaustion, maintaining 60 revolutions per minute. | NR, NR (NR) | PPG | Polar S810i | x | x | x | ✓ | x | x | x | ✓ | x | x | x |
|  |  |  | D_diabetes(type 2)_ | 9 | 56 % | 55.6 |  |  |  |  |  |  |  |  |  |  |  |  |  |  |  |
| Steptoe et al. ([183](#_ENREF_183)) | 2002 | COH | HOA | 228 | 46 % | 52.3 | 5 min of mirror-tracing stress task (Lafayette Instruments) and 5 min of computerized Stroop colour-word task in random order | NR, NR (10 min) | ECG | VU-AMS | ✓ | ✓ | ✓ | ✓ | x | x | x | x | ✓ | x | x |
| Steptoe et al. ([184](#_ENREF_184)) | 2005 | CC | HOA | 132 | 58 % | 70.5 | (1) Verbal Paired Associates (VPA-WMS-III), 3 trials, 10 min rest; (2) matrix reasoning (MR-WAIS-III), 5 min, 10 min rest; and (3) verbal paired associates recall and recognition (VPA-II) | NR, NR  (5 min) | ECG | VU-AMS | x | ✓ | x | ✓ | ✓ | x | x | x | x | x | x |
|  |  |  | HA | 26 | 58 % | 33.5 |  |  |  |  |  |  |  |  |  |  |  |  |  |  |  |
| Steptoe & Marmot ([185](#_ENREF_185)) | 2005 | COH | HOA | 209 | 46 % | 51.7 | 5 min of computerized Stroop colour-word task and 5 min of mirror-tracing stress task | S, NR (10 min) | ECG | VU-AMS | ✓ | ✓ | ✓ | ✓ | x | x | x | x | ✓ | x | x |
| Steptoe et al. ([186](#_ENREF_186)) | 2006 | COH | HOA | 228 | 45 % | 52.3 | 5 min of mirror-tracing stress task (Lafayette Instruments) and 5 min of computerized Stroop colour-word task in random order | NR, NR (5 min) | ECG | VU-AMS | ✓ | x | x | ✓ | x | x | x | x | ✓ | x | x |
| Takahashi et  al. ([187](#_ENREF_187)) | 2003 | ITS | HOA | 7 | 43 % | 72 | Graded exercise tests on motor-driven treadmill (5 % slope), starting at 3.0 km/h, increments of 0.5 km/h every 3 min, until exhaustion. Two trials: (1) on land; and (2) in water, one week apart | U, NR  (3 min) | ECG | MR-30, Teac | ✓ | x | x | ✓ | x | x | ✓ | ✓ | ✓ | x | x |
| Virtanen et al. ([188](#_ENREF_188)) | 2007 | CC | HOA | 114 | 29 % | 58 | Sign- and symptom-limited maximal exercise test on bicycle ergometer with a start level of 20 – 30 W with stepwise increases of 10 – 30 W every minute | S, NR (1 min) | ECG | GE Medical Systems, Waukesha, WI, USA | ✓ | x | x | ✓ | x | x | x | x | x | x | x |
|  |  |  | D_CAD_ | 112 | 28 % | 59 |  |  |  |  |  |  |  |  |  |  |  |  |  |  |  |
| Wang et al. ([189](#_ENREF_189)) | 2016 | CC | HOA | 20 | 74 % | 50.7 | Submaximal exercise protocol on bicycle ergometer for 16 min at 80 rpm’s starting at a workload of 50 watts, increasing to 75 watts at 4 min, and 100 watts at 8 min, as tolerated by the subject. | S, NR (5 min) | ECG | NR | x | ✓ | x | ✓ | x | x | x | x | x | x | x |
|  |  |  | D_CAD_ | 87 | 28 % | 58.1 |  |  |  |  |  |  |  |  |  |  |  |  |  |  |  |
| Wawrzyniak  et al. ([190](#_ENREF_190)) | 2016 | COH | HOA | 262 | 61 % | 63.5 | 5 min of mirror-tracing stress task (Campden Instruments Ltd.) counterbalanced before or after 5 min of Stroop colour-word task | S, NR  (5 min) | ECG | ActiHeart  monitoring  device | ✓ | x | x | ✓ | x | x | ✓ | x | ✓ | x | ✓ |
| Wittstein et  al. ([191](#_ENREF_191)) | 2019 | CC | HOA | 26 | NR | 67.7 | 15 min treadmill walking at preferred walking speed (PWS). Subsequently, 2 x 15 min experimental trial, in counterbalanced order: (1) gait synchronization task; (2) fast walking (at 125 % PWS) | S, NR (8 min) | ECG | Biopac MP-150 | x | x | x | ✓ | x | x | x | x | x | x | x |
|  |  |  | HA | 25 | NR | 24.6 |  |  |  |  |  |  |  |  |  |  |  |  |  |  |  |
| Wood et al. ([192](#_ENREF_192))  (1) Study types according to: ***RCT*** = Randomized Controlled Trials, ***CCT*** = Controlled Clinical Trial, **CC** = Case-Control Study, **COH** = Cohort Study, **ITS** = Interrupted time series NR = not reported  (2) ***HA*** = healthy adult, ***HOA*** = healthy middle-aged to older adults (≥ 50 years); ***COG*** = older adults with cognitive impairment; **D**: pathological group_(type of disease)_  (3) (a) posture: **S** = seated, **L** = lying in supine position, **U** = upright/standing; (b) breathing pattern: **SB** = spontaneous breathing pattern, **PB** = paced breathing pattern  (4) **E*CG*** = electrocardiography, ***PPG*** = photoplethysmography, **fITI** = functional infrared imaging | 2002 | CC | HOA | 19 | 79 % | 85 | Reaction time task (5 min): Response to visual (light) and auditory (sound) cues by pressing a switch as quickly as possible. Two sets of 50 auditory and 50 visual cues in counterbalanced order | S, SB (5 min) | ECG | Biopac data acquisition board | x | x | x | x | ✓ | x | x | x | x | x | x |
|  |  |  | HA | 28 | NR | 21 |  |  |  |  |  |  |  |  |  |  |  |  |  |  |  |

## Appendix B: Data Extraction Table

| Table A2: Data Extraction Table of Reactivity of HRV in Healthy Middle-Aged to Older Adults | | | | | | | | | | | | | | | | | | | | | | | | | | | |  |
| --- | --- | --- | --- | --- | --- | --- | --- | --- | --- | --- | --- | --- | --- | --- | --- | --- | --- | --- | --- | --- | --- | --- | --- | --- | --- | --- | --- | --- |
| Reference | | Exercise | | HRV Reactivity | | | | | | | | | | | | | | | | | | | | Moderators | | | | |
|  |  |  |  | time-domain | | | | | | frequency-domain | | | | | | | | non-linear | | | | | | significant effect (p < 0.05) | | | no effect | |
| Author | Year | Type ^(1)^ | Intensity ^(2)^ | mRR [ms] | SDNN [ms] | SDRR [ms] | pNN50 [%] | RMSSD [ms] |  | VLF [ms^2^] | LF [ms^2^] | LFnu [nu] | RSA [ms^2^] | HF [ms^2^] | HFnu [nu] | LF/HF [%] |  | SD1 [ms] | SD2 [ms] | SD1/SD2 [%] | DF𝛼1 [] | SampEn [] | CoV [] | Variable | Relations | Statistics | Variables | |
|  |  |  |  |  |  |  |  |  |  |  |  |  |  |  |  |  |  |  |  |  |  |  |  |  |  |  |  |  |
| Ahmadian et al. ([150](#_ENREF_150)) | 2015 | PHYS^C^ | light to max | ↓ | ↓ |  | ↘ | ↓ |  |  |  |  |  |  |  |  |  |  |  |  |  |  |  | NR | NR | NR | NR | |
| Alves et al. ([151](#_ENREF_151)) | 2011 | PHYS^C^ | moderate to vigorous |  |  |  |  |  |  |  |  | → |  |  | → | ↘ |  |  |  |  |  |  |  | NR | NR | NR | NR | |
| Archiza et al. ([152](#_ENREF_152)) | 2013 | PHYS^B^ | light, moderate, vigorous |  |  |  |  | ↗ |  |  | ↘ | ↓ |  | ↗ | ↑ |  |  |  |  |  |  |  |  | Intensity | Higher inspiratory resistive loading intensities reduced the absolute values of RMSSD, HF and SD1 during exercise and decreased the reactivity of RMSSD. Changes in mRR, SDNN, LF, LF/HF and SD2 were not associated with intensity. | Rep.-measures ANOVA with post-hoc Tukey Kramer | NR | |
| Bartels et al. ([153](#_ENREF_153)) | 2003 | PHYS^C^ | very light to max |  |  |  |  |  |  |  | ↑ | ↑ |  | → | → | ↑ |  |  |  |  |  |  |  | Population | No differences were found in any of the measured indexes at maximal exercise intensity between HOA and patients with COPD. | Spearman row, Wilcoxon rank sum test | HRV reactivity was not related to age, gender, body mass index and spirometric parameters | |
| Beer et al. ([154](#_ENREF_154)) | 2017a | PHYS^R^ | light |  | ↓ |  |  | → |  |  |  |  |  |  |  |  |  |  |  |  |  |  |  | Population | Mean SDNN values on-task were lower among patients post-stroke compared to HOA. RMSSD was higher in HOA in all testing conditions except DUAL (Z = 0.9, p = 0.19). | Friedman test, Wilcoxon signed rank sum test, | SDNN values during cognitive tasks did not correlate with task performance | |
|  |  | COGN | NR |  | ↓ |  |  | → |  |  |  |  |  |  |  |  |  |  |  |  |  |  |  |  |  |  |  |  |
|  |  | DUAL | moderate |  | → |  |  | ↓ |  |  |  |  |  |  |  |  |  |  |  |  |  |  |  |  |  |  |  |  |
|  |  |  |  |  |  |  |  |  |  |  |  |  |  |  |  |  |  |  |  |  |  |  |  |  |  |  |  |  |
| Beer et al. ([155](#_ENREF_155)) | 2017b | PHYS^R^ | light | ↓ | ↘ |  |  | → |  |  |  |  |  |  |  |  |  |  |  |  |  |  |  | Task & Intensity  Population | The reactivity of mRR was higher during cycling than the cognitive task. RMSSD showed no reactivity to light intensity handgrip exercise whereas a significant drop was observed during moderate intensity cycling and dual task.  Reactivity of mRR was lower among patients post- stroke compared to HOA. | T-test | NR | |
|  |  | PHYS^C^ | moderate | ↓ | → |  |  | ↓ |  |  |  |  |  |  |  |  |  |  |  |  |  |  |  |  |  |  |  |  |
|  |  | COGN | NR | ↓ | ↘ |  |  | → |  |  |  |  |  |  |  |  |  |  |  |  |  |  |  |  |  |  |  |  |
|  |  | DUAL | moderate | ↓ | ↘ |  |  | ↓ |  |  |  |  |  |  |  |  |  |  |  |  |  |  |  |  |  |  |  |  |
|  |  |  |  |  |  |  |  |  |  |  |  |  |  |  |  |  |  |  |  |  |  |  |  |  |  |  |  |  |
| Betz et al. ([156](#_ENREF_156)) | 2017 | COGN | NR |  | → |  |  |  |  |  |  |  |  |  |  |  |  |  |  |  |  |  |  | Stress | HRV measured with SDNN was negatively correlated with strain ratings during the mental arithmetic task in HOA, indicating that a reduction in HRV was associated with higher subjective strain, r(19) = − 0.47, p = 0.042. | Mixed ANOVA, Pearson Correlation | SDNN reactivity did not differ between HOA vs. Tinnitus. Correlation between SDNN and mean HR. | |
| Cacioppo et  al. ([157](#_ENREF_157)) | 2000 | COGN | NR |  |  |  |  |  |  |  |  |  | ↓ |  |  |  |  |  |  |  |  |  |  | NR | NR | T-test | Resting values and reactivity of RSA did not differ between dementia caregivers and non-caregivers. | |
| Capuana et al. ([158](#_ENREF_158)) | 2012 | COGN | low to high |  |  |  |  |  |  |  |  |  | → |  |  |  |  |  |  |  |  |  |  | Population | RSA reactivity did not differ between groups (i.e. younger adults compared to their older counterparts) | Repeated-measures ANOVA | RSA values during the task were not related to cognitive task performance. | |
| Christensen et al. ([159](#_ENREF_159)) | 2014 | COGN | NR |  |  |  |  |  |  |  | ↓ |  |  |  |  |  |  |  |  |  |  |  |  | Difficulty | Main effect of task difficulty on HRV reactivity was not significant F(2,50) = 1.88, but the change in HRV between the one-back (M = 0.51, SD = 0.17) and two-back (M = 0.98, SD = 0.14) was statistically significant (p = .01) in HOA. | Mixed ANOVA, Bonferroni adjusted t-tests | Population (HOA, Aphasia)  Task type | |
| Collste et al. ([160](#_ENREF_160)) | 2014 | COGN | NR |  | ↑ |  |  |  |  |  |  |  |  |  |  |  |  |  |  |  |  |  |  | Population | SDNN reactivity was higher in patients with cardiomyopathy compared to HOA (p = 0.002). However, the absolute on-task values did not differ between groups. | Non-parametric Mann-Whitney U-test | NR | |
| Corrêa et al. ([161](#_ENREF_161)) | 2013 | PHYSC | moderate | ↓ |  |  |  | ↓ |  |  |  |  |  |  |  |  |  | ↓ | ↓ |  |  |  |  | Age | A significant positive correlation between age and absolute values of mRR intervals (r = 0.27; p = 0.015) during the test was observed. HRV reactivity was not age dependent. | Pearson or Spearman correlation, ANOVA, t-test | Sex | |
| Crowley et al. ([162](#_ENREF_162)) | 2016 | COGN | NR |  |  |  |  |  |  |  |  |  |  | ↓ |  |  |  |  |  |  |  |  |  | Age  HRV rest | Age correlated with HRV reactivity (r = - 0.098, p = 0.006; r = 0.142, p = 0.000) before and after adjusting for respiratory rate.  Resting HRV values correlated with on-task HRV before and after adjusting for respiratory rate (r = 0.86, p < .0001; r = 0.72, p < .0001). | Multiple linear regression | NR | |
| Davrath et al. ([163](#_ENREF_163)) | 2006 | PHYS^C^ | light to near maximal |  |  |  |  |  |  |  | ↓ |  |  | ↓ |  |  |  |  |  |  |  |  |  | NR | NR | NR | NR | |
| Dourado et al. ([164](#_ENREF_164)) | 2010 | PHYS^C^ | light to vigorous |  |  |  |  |  |  |  |  |  |  |  |  |  |  | ↘ |  |  |  |  |  | NR | NR | NR | NR | |
| Hamer &  Steptoe ([165](#_ENREF_165)) | 2007 | COGN | NR |  |  |  |  | ↓ |  |  |  |  |  |  |  |  |  |  |  |  |  |  |  | Fitness level  Stress | Higher physical fitness levels were related to lower HRV reactivities during cognitive stress (β = -0.23; p = 0.02).  Higher HRV reactivities were related to greater increases of TNF-ɑ during cognitive stress (r = -0.20; p = 0.01). | Linear regression analysis | No associations between HRV reactivity and IL-6 stress response were observed | |
| Junior et al. ([166](#_ENREF_166)) | 2019 | PHYS^C^ | NR |  |  |  |  | NR |  |  |  |  |  |  |  |  |  |  |  |  |  |  |  | NR | NR | NR | NR | |
| Kaltsatou et al. ([167](#_ENREF_167)) | 2020 | PHYS^C^ | light to moderate |  |  |  |  |  |  | → | → |  |  | ↗ |  | ↘ |  | → | ↗ |  | ↘ |  |  | Age  Duration  Body fat | In older adults, LF, LF/HF, and DFA-ɑ1 during exercise were suppressed compared to younger and middle-aged adults whilst controlling for aerobic fitness (not sign.: VLF, HF, CoV, SD1, SD2, and DFA-ɑ2).  For the overall study population, there was a main effect of time during exercise in CoV, and SD2 (not sig.: VLF, LF, HF, LF/HF, SD1, DFA-ɑ1, and DFA-ɑ2).  When additionally controlling for % body fat, all age- and time-related main or interaction effects vanished. | Mixed ANCOVA, post-hoc t-tests | NR | |
| Karavirta et al. ([168](#_ENREF_168)) | 2009 | PHYS^C^ | light to max |  |  |  |  |  |  |  | NR |  |  | NR |  |  |  |  |  |  | ↓ |  |  | Intensity  Type of  Exercise  Intervention  Age | DFA-ɑ1 decreased gradually with increasing intensities from a mean value of 1.37 (SD = 0.22) at 30% to 0.54 (SD = 0.19) at 90% of maximal power.  After a 21-week intervention, DFA-ɑ1 increased at light, and decreased at moderate exercise intensities, but only in the combined endurance & strength training group. At moderate to high intensities there was an increase in HF-HRV in all groups except the strength training group. At light intensities, LF-HRV increased in the endurance and combined endurance and strength training group.  Relative changes in 𝛼1 correlated with age at the exercise intensity of 90 % of maximal power (r = - 0.44, p = 0.023). | Paired t-test, Repeated measures ANOVA and Bonferroni post-hoc tests | None of the groups showed significant changes in HF or LF during low-intensity exercise after the 21 weeks of exercise intervention.  There were no correlations between the changes in VO_2max_ and the changes in 𝛼1 at any relative exercise intensity. | |
| Kunz-Ebrecht et al. ([169](#_ENREF_169)) | 2003 | COGN | NR |  |  |  |  | ↓ |  |  |  |  |  |  |  |  |  |  |  |  |  |  |  | Stress | A higher cortisol response to stress was associated with larger stress-induced inhibition of HRV (F(1/102) = 5.54, p = 0.021) and more experienced subjective stress during the tasks (F(1/154) = 3.87, p = 0.05). | Repeated measures ANOVA | Gender | |
| Kuraoka et al. ([170](#_ENREF_170)) | 2019 | COGN | NR |  |  |  |  |  |  |  | ↓ |  |  | ↓ |  | → |  |  |  |  |  |  |  | Task | HRV responses were significant during mental arithmetic but not during mirror tracing task. | ANOVA and Bonferroni post-hoc tests | Population (HOA vs. HA) | |
| Lin et al. ([171](#_ENREF_171)) | 2014 | COGN | NR |  |  |  |  |  |  |  | ↓ |  |  | ↓ |  |  |  |  |  |  |  |  |  | Task  Executive Function | The Stroop task induced larger decreases in LF-HRV (mean difference = 0.22, SE = 0.04; t_[424]_ = 5.50) and HF-HRV (mean difference = 0.38, SE = 0.03; t_[424]_ = 12.67) compared to mental arithmetic.  Higher LF-HRV reactivities predicted better executive function (B[SE] = -0.09 (0.05), t_[406]_ = -2.00, p = 0.046), whereas HF-HRV did not (controlling for all covariates (i.e., age, gender, education, hypertension, diabetes, heart attack, regularly smoking, alcohol intake, perceived control, depressive symptoms). | Repeated mea-sures ANOVA, Bonferroni post-hoc tests  Multiple linear Regression | NR | |
| Lin et al. ([172](#_ENREF_172)) | 2017 | COGN | NR |  |  |  |  |  |  |  |  |  |  | ↓ |  |  |  |  |  |  |  |  |  | Cognition  Brain activity | Higher HRV reactivity was related to worse cognition (i.e. MoCA (r = 0.39, p = 0.022) and episodic memory (r = 0.48, p = 0.004), but not to executive function (r = 0.29, p = 0.087) (controlled for age and sex)).  The relation between Alzheimer’s disease-associated neurodegeneration, cognitive performance and HF-HRV were mediated by anterior cingulate cortex hyperactivity. | Partial Pearson’s correlations | The reactivities of HF-HRV were unaffected by the clinical phenotype. | |
| Machado- Vidotti et al. ([173](#_ENREF_173)) | 2014 | PHYS^R^ | very light to max |  |  |  |  | ↓ |  |  |  | ↗ |  | ↓ | ↓ | ↗ |  | ↓ |  |  |  |  |  | Modality  Intensity | Reactivities of LFnu and HF were more pronounced during upper limb than lower limb exercise (p < 0.05).  HRV reactivity increased with increasing exercise loads. | Repeated mea-sures ANOVA, Tukey-Kramer post-hoc tests | The reactivity of HRV was not significant for exercise loads below 20 % of 1RM | |
| Mayumi et al. ([174](#_ENREF_174)) | 2008 | PHYS^C^ | very light to vigorous |  |  |  |  |  |  |  |  |  |  | ↓ |  |  |  |  |  |  |  |  |  | Intensity  Population | HF-HRV decreased with increasing exercise intensities  Hypertensive patients showed significantly lower HF power during 25-watt load exercise than HOA (3.55 ± 1.29 vs. 4.40 ± 1.31 msec2, p < 0.01). | T-test | NR | |
| Millar et al. ([175](#_ENREF_175)) | 2011 | PHYS^R^ | light |  | → |  | → | → |  |  |  |  |  |  |  |  |  |  |  |  | → | ↓ |  | Duration | SampEn was reduced for longer durations of constant load isometric handgrip (≤ 1 min) while remaining unchanged for shorter contractions (< 30 s). Reactivities of SDNN, RMSSD, pNN50 and DFA-ɑ1 were not significantly related to duration. | One-way repeated measures ANOVA | NR | |
| Norcliffe-Kaufmann et al. ([176](#_ENREF_176)) | 2016 | COGN | NR | ↘ | NR |  | NR | NR |  |  |  |  |  | NR |  |  |  |  |  |  |  |  |  | NR | NR | NR | NR | |
| Perini et al. ([177](#_ENREF_177)) | 2000 | PHYS^C^ | between very light to max |  |  |  |  |  |  |  |  | ↓ |  |  | ↑ | ↓ |  |  |  |  |  |  |  | Intensity  Gender | HF% and HFnu increased while total power, LF%, LFnu and LF/HF decreased progressively with increasing exercise intensities. VLF% was not related to exercise intensity. These HRV changes were not observed at very light to light exercise intensities.  At VO_2peak,_ HF% was higher in men than women. No other HRV differences between gender were found. | T-test, linear regression | NR | |
| Perpetuini et al. ([178](#_ENREF_178)) | 2019 | COGN | NR |  |  |  |  |  |  |  |  |  |  |  |  | ↑ |  |  |  |  |  |  |  | Population | Alzheimer’s Disease patients showed larger increases in LF/HF during memory recall (t = 2.073, df = 23, p = 0.049), which was not significant after correction for multiple comparisons. | Pairwise t-test with Bonferroni correction | NR | |
| Petrofsky et al. ([179](#_ENREF_179)) | 2009 | PHYS^R^ | very light to max |  |  |  |  |  |  |  |  |  |  |  |  |  |  |  |  |  |  |  | → | Intensity | At 40 % MVC, the HRV coefficient of variation increased significantly in younger adults but remained unchanged in HOA and diabetes patients. HRV reactivities were not significant at lower intensities (10 % and 25 % MVC). | ANOVA, t-test | NR | |
| Piepoli et al. ([180](#_ENREF_180)) | 1996 | PHYS^R^ | light to max | ↓ |  | NR |  |  |  |  | → |  |  | ↓ |  |  |  |  |  |  |  |  |  | Population | HOA showed larger HRV reactivities compared to chronic heart failure patients. | T-test | No changes in HRV reactivity were observed after a 6-week handgrip exercise program | |
| Rodrigues et al. ([181](#_ENREF_181)) | 2019 | PHYS^C^ | moderate to max |  |  |  |  | ↘ |  |  | ↗ |  |  | ↘ |  | ↗ |  |  |  |  |  |  |  | NR | NR | Mixed-model analysis | HRV during exercise did not change after an exercise intervention with different periodization models. | |
| Sales et al. ([182](#_ENREF_182)) | 2011 | PHYS^C^ | light to max |  |  |  |  | NR |  |  |  |  |  |  |  |  |  | NR |  |  |  |  |  | NR | NR | NR | NR | |
| Steptoe et al. ([183](#_ENREF_183)) | 2002 | COGN | difficult |  |  |  |  | ↘ |  |  |  |  |  |  |  |  |  |  |  |  |  |  |  | Socioeconomic status | Heart rate variability responses varied with grade of employment (p < 0.05), with smaller reactions in the group with medium socio-economic status. | Repeated mea-sures ANOVA | HRV responses was not related to smoking, alcohol consumption and sedentary lifestyle | |
| Steptoe et al. ([184](#_ENREF_184)) | 2005 | COGN | NR |  |  |  |  | → |  |  |  |  |  |  |  |  |  |  |  |  |  |  |  | Population | HRV reactivity was blunted in healthy older adults compared to younger adults (F(2, 210) = 2.99, p = 0.023), after adjustment for gender, body mass index, chronic illness index and medication count. | Repeated mea-sures ANOVA | Education status | |
| Steptoe & Marmot ([185](#_ENREF_185)) | 2005 | COGN | NR |  |  |  |  | ↓ |  |  |  |  |  |  |  |  |  |  |  |  |  |  |  | NR | NR | Repeated mea-sures ANOVA | Gender | |
| Steptoe et al. ([186](#_ENREF_186)) | 2006 | COGN | difficult |  |  |  |  | → |  |  |  |  |  |  |  |  |  |  |  |  |  |  |  | NR | NR | NR | NR | |
| Takahashi et  al. ([187](#_ENREF_187)) | 2003 | PHYS^C^ | light to max |  |  |  |  |  |  |  |  |  |  | ↓ |  |  |  |  |  |  |  |  |  | NR | NR | Repeated mea-sures ANOVA, t-test | HF-HRV reactivity was unaffected by exercise modality, gait speed and gender. | |
| Virtanen et al. ([188](#_ENREF_188)) | 2007 | PHYS^C^ | light to max |  | ↓ |  |  | ↓ |  |  |  |  |  |  |  |  |  | ↓ | ↓ |  |  |  |  | Population  Heart rate | HRV at exercise was lower in coronary artery disease (CAD) patients compared to HOA.  There was a negative correlation between mean HR and RR intervals (0.57 < \|r\| < 0.66, p < 0.001) during exercise. | Mann-Whitney U-test, Pearson’s correlation | NR | |
| Wang et al. ([189](#_ENREF_189)) | 2016 | PHYS^C^ | up to max | ↓ |  | NR |  | NR |  |  | NR |  |  | NR |  | NR |  |  |  |  |  |  |  | Population | Coronary artery disease (CAD) patients showed higher mRR at peak exercise compared to HOA. | ANOVA | NR | |
| Wawrzyniak  et al. ([190](#_ENREF_190)) | 2016 | COGN | NR |  |  |  |  | ↓ |  |  |  |  |  |  |  |  |  |  |  |  |  |  |  | Mean reaction time | Greater HRV reactivities (higher reductions) were associated with slower average response times (B = - 0.0018, SE = 0.001, R^2^ = 0.72, p = 0.05) with HRV at rest, employment grade, and stress task appraisal as covariates (p < 0.05). | Robust regression | The standard deviation and variability of reaction times were not significantly associated with HRV reactivity (p = 0.19, p = 0.10). | |
| Wittstein et  al. ([191](#_ENREF_191)) | 2019 | PHYS^C^ | moderate | NR |  | NR |  |  |  |  |  |  |  |  |  |  |  |  |  |  | NR |  | NR | Population  Modality | Older adults exhibited slower mRR (F = 5.313, p = 0.026, partial η^2^ = 0.102) and less complex (lower standard error) R–R interval patterns (F = 21.181, p < 0.001, partial η^2^ = 0.311) than the younger group during the synchronization task. There was no difference for CoV and DFA-ɑ.  DFA-ɑ was elevated during gait synchronization compared to walking at preferred walking speed (F = 4.085, p = 0.049, partial η^2^ = 0.080). There was no difference for mRR, SDRR, and CoV. | MANOVA | Intensity | |
| Wood et al. ([192](#_ENREF_192)) | 2002 | COGN | NR | ↓ | ↓ |  |  |  |  |  |  | ↑ |  |  | ↓ |  |  |  |  |  |  |  |  | Population | Older adults demonstrated lower SDNN during the cognitive task compared to their younger counterparts (p < 0.05). There were no between group differences in mRR, LFnu and HFnu during the test. | Mixed ANOVA | NR | |

(1) PHYS = Physical Exercise (C = Cardiorespiratory exercise, R = Resistance exercise, B = Resistive breathing exercise); ↑ = significant (p < 0.05) increase from resting HRV
COGN = Cognitive task; ↓ = significant (p < 0.05) decrease from resting HRV
SIM = Simultaneous cognitive-physical exercise → = no significant change from resting HRV

(2) For physical stressors / exercise: According to the classification of physical exercise intensities by the American College of Sports Medicine NR = not reported

For cognitive stressors / tasks: According to level of complexity of cognitive tasks described by the author or assessed via questionnaires ↗︎/↘︎ = increasing / decreasing trend (not significant or not statistically tested)

## Appendix C: PRISM checklist

| Table A3: PRISM checklist | | | |
| --- | --- | --- | --- |
| Section/topic | # | Checklist item | Reported on line number |
| TITLE | | |  |
| Title | 1 | Identify the report as a systematic review, meta-analysis, or both. | 0 |
| ABSTRACT | | |  |
| Structured summary | 2 | Provide a structured summary including, as applicable: background; objectives; data sources; study eligibility criteria, participants, and interventions; study appraisal and synthesis methods; results; limitations; conclusions and implications of key findings; systematic review registration number. | 30 - 57 |
| INTRODUCTION | | |  |
| Rationale | 3 | Describe the rationale for the review in the context of what is already known. | 60 - 212 |
| Objectives | 4 | Provide an explicit statement of questions being addressed with reference to participants, interventions, comparisons, outcomes, and study design (PICOS). | 213 - 217 |
| METHODS | | |  |
| Protocol and registration | 5 | Indicate if a review protocol exists, if and where it can be accessed (e.g., Web address), and, if available, provide registration information including registration number. | 244 - 257 |
| Eligibility criteria | 6 | Specify study characteristics (e.g., PICOS, length of follow-up) and report characteristics (e.g., years considered, language, publication status) used as criteria for eligibility, giving rationale. | 258 - 275 |
| Information sources | 7 | Describe all information sources (e.g., databases with dates of coverage, contact with study authors to identify additional studies) in the search and date last searched. | 276 - 279 |
| Search | 8 | Present full electronic search strategy for at least one database, including any limits used, such that it could be repeated. | 280 - 291 |
| Study selection | 9 | State the process for selecting studies (i.e., screening, eligibility, included in systematic review, and, if applicable, included in the meta-analysis). | 292 - 308 |
| Data collection process | 10 | Describe method of data extraction from reports (e.g., piloted forms, independently, in duplicate) and any processes for obtaining and confirming data from investigators. | 309 - 312 |
| Data items | 11 | List and define all variables for which data were sought (e.g., PICOS, funding sources) and any assumptions and simplifications made. | 313 - 341 |
| Risk of bias in individual studies | 12 | Describe methods used for assessing risk of bias of individual studies (including specification of whether this was done at the study or outcome level), and how this information is to be used in any data synthesis. | 342 - 356 |

*From:*  Moher D, Liberati A, Tetzlaff J, Altman DG, The PRISMA Group (2009). Preferred Reporting Items for Systematic Reviews and Meta-Analyses: The PRISMA Statement. PLoS Med 6(7): e1000097. doi:10.1371/journal.pmed1000097

For more information, visit: **www.prisma-statement.org**.

*From:*  Moher D, Liberati A, Tetzlaff J, Altman DG, The PRISMA Group (2009). Preferred Reporting Items for Systematic Reviews and Meta-Analyses: The PRISMA Statement. PLoS Med 6(7): e1000097. doi:10.1371/journal.pmed1000097

For more information, visit: **www.prisma-statement.org**.

| Section/topic | # | Checklist item | Reported in line number |
| --- | --- | --- | --- |
| Summary measures | 13 | State the principal summary measures (e.g., risk ratio, difference in means). | 374 - 402 |
| Synthesis of results | 14 | Describe the methods of handling data and combining results of studies, if done, including measures of consistency (e.g., I^2^) for each meta-analysis. | 374 - 402 |
| Risk of bias across studies | 15 | Specify any assessment of risk of bias that may affect the cumulative evidence (e.g., publication bias, selective reporting within studies). | 408 - 416 |
| Additional analyses | 16 | Describe methods of additional analyses (e.g., sensitivity or subgroup analyses, meta-regression), if done, indicating which were pre-specified. | 404 - 407 |
| RESULTS | | |  |
| Study selection | 17 | Give numbers of studies screened, assessed for eligibility, and included in the review, with reasons for exclusions at each stage, ideally with a flow diagram. | 418 - 429 |
| Study characteristics | 18 | For each study, present characteristics for which data were extracted (e.g., study size, PICOS, follow-up period) and provide the citations. | 430 - 486 |
| Risk of bias within studies | 19 | Present data on risk of bias of each study and, if available, any outcome level assessment (see item 12). | 487 - 518 |
| Results of individual studies | 20 | For all outcomes considered (benefits or harms), present, for each study: (a) simple summary data for each intervention group (b) effect estimates and confidence intervals, ideally with a forest plot. | 519 - 632 |
| Synthesis of results | 21 | Present results of each meta-analysis done, including confidence intervals and measures of consistency. | 633 - 694 |
| Risk of bias across studies | 22 | Present results of any assessment of risk of bias across studies (see Item 15). | 633 - 694 |
| Additional analysis | 23 | Give results of additional analyses, if done (e.g., sensitivity or subgroup analyses, meta-regression [see Item 16]). | 633 - 694 |
| DISCUSSION | | |  |
| Summary of evidence | 24 | Summarize the main findings including the strength of evidence for each main outcome; consider their relevance to key groups (e.g., healthcare providers, users, and policy makers). | 695 - 918 |
| Limitations | 25 | Discuss limitations at study and outcome level (e.g., risk of bias), and at review-level (e.g., incomplete retrieval of identified research, reporting bias). | 1076 - 1091 |
| Conclusions | 26 | Provide a general interpretation of the results in the context of other evidence, and implications for future research. | 1092 – 1116 |
| FUNDING | | |  |
| Funding | 27 | Describe sources of funding for the systematic review and other support (e.g., supply of data); role of funders for the systematic review. | 1125 - 1130 |

*From:*  Moher D, Liberati A, Tetzlaff J, Altman DG, The PRISMA Group (2009). Preferred Reporting Items for Systematic Reviews and Meta-Analyses: The PRISMA Statement. PLoS Med 6(7): e1000097. doi:10.1371/journal.pmed1000097

For more information, visit: **www.prisma-statement.org**.

# References

150. Ahmadian M, Dabidi Roshan V. Dynamics of heart rate variability in different ages: effect of the arm crank and cycle ergometer Protocols. The Journal of sports medicine and physical fitness. 2015;56.

151. Alves Naiane Ferraz B, Porpino Suênia Karla P, Brito Aline de F, da N, Thereza Karolina S, Freitas Rosimeire de S, et al. Autonomic modulation and chronotropic activity during aerobic exercise in patients using atenolol. ConScientiae Saude. 2011;10(1):51-8.

152. Archiza B, Simões RP, Mendes RG, Fregonezi GAF, Catai AM, Borghi-Silva A. Acute effects of different inspiratory resistive loading on heart rate variability in healthy elderly patients. Brazilian Journal of Physical Therapy. 2013;17(4):401-8.

153. Bartels Matthew N, Jelic S, Ngai P, Basner Robert C, DeMeersman Ronald E. High-frequency modulation of heart rate variability during exercise in patients with COPD. Chest. 2003;124(3):863-9.

154. Beer Noa R, Soroker N, Bornstein Nathan M, Leurer Michal K. Association between cardiac autonomic control and cognitive performance among patients post stroke and age-matched healthy controls-an exploratory pilot study. Neurological sciences : official journal of the Italian Neurological Society and of the Italian Society of Clinical Neurophysiology. 2017;38(11):2037-43.

155. Beer NR, Bornstein N, Leurer MK. The cardiac autonomic nervous system response to different daily physiotherapy tasks in patients at the sub-acute phase post-ischemic stroke and healthy controls. European Journal of Neurology. 2017;24:55.

156. Betz Linda T, Mühlberger A, Langguth B, Schecklmann M. Stress Reactivity in Chronic Tinnitus. Scientific reports. 2017;7:41521.

157. Cacioppo JT, Burleson MH, Poehlmann KM, Malarkey WB, Kiecolt-Glaser JK, Berntson GG, et al. Autonomic and neuroendocrine responses to mild psychological stressors: Effects of chronic stress on older women. Annals of Behavioral Medicine. 2000;22(2):140-8.

158. Capuana LJ, Dywan J, Tays WJ, Segalowitz SJ. Cardiac workload and inhibitory control in younger and older adults. Biological Psychology. 2012;90(1):60-70.

159. Christensen Stephanie C, Wright Heather H. Quantifying the effort individuals with aphasia invest in working memory tasks through heart rate variability. American journal of speech-language pathology. 2014;23(2):S361-S71.

160. Collste O, Tornvall P, Sundin Ö, Alam M, Frick M. No myocardial vulnerability to mental stress in Takotsubo stress cardiomyopathy. PloS one. 2014;9(4):e93697.

161. Corrêa FR, da Silva A, M A, Bianchim MS, Crispim de A, Guerra RLF, et al. Heart rate variability during 6-min walk test in adults aged 40 years and older. International journal of sports medicine. 2013;34(2):111-5.

162. Crowley Olga V, Kimhy D, McKinley Paula S, Burg Matthew M, Schwartz Joseph E, Lachman Margie E, et al. Vagal Recovery From Cognitive Challenge Moderates Age-Related Deficits in Executive Functioning. Research on aging. 2016;38(4):504-25.

163. Davrath Linda R, Akselrod S, Pinhas I, Toledo E, Beck A, Elian D, et al. Evaluation of autonomic function underlying slow postexercise heart rate recovery. Medicine and science in sports and exercise. 2006;38(12):2095-101.

164. Dourado VZ, Banov MC, Marino MC, de S, V L, Antunes LCdO, et al. Training & Testing. A Simple Approach to Assess VT During a Field Walk Test. Int J Sports Med. 2010;31(10):698-703.

165. Hamer M, Steptoe A. Association between physical fitness, parasympathetic control, and proinflammatory responses to mental stress. Psychosomatic medicine. 2007;69(7):660-6.

166. Junior Adalberto F, Schamne Julio C, Perandini Luiz Augusto B, Chimin P, Okuno Nilo M. Effects of Walking Training with Restricted Blood Flow on HR and HRV Kinetics and HRV Recovery. Int J Sports Med. 2019;40(9):585-91.

167. Kaltsatou A, Flouris Andreas D, Herry Christophe L, Notley Sean R, Seely Andrew JE, Beatty Heather W, et al. Age differences in cardiac autonomic regulation during intermittent exercise in the heat. European journal of applied physiology. 2020;120(2):453-65.

168. Karavirta L, Tulppo Mikko P, Laaksonen David E, Nyman K, Laukkanen Raija T, Kinnunen H, et al. Heart rate dynamics after combined endurance and strength training in older men. Medicine and science in sports and exercise. 2009;41(7):1436-43.

169. Kunz-Ebrecht SR, Mohamed-Ali V, Feldman PJ, Kirschbaum C, Steptoe A. Cortisol responses to mild psychological stress are inversely associated with proinflammatory cytokines. Brain, Behavior, and Immunity. 2003;17(5):373-83.

170. Kuraoka H, Kurosaka C, Wada C, Miyake S. Effect of age on heart rate responses and subjective mental workload during mental tasks. 2019;827:316-21.

171. Lin F, Heffner K, Mapstone M, Chen Ding-Geng D, Porsteisson A. Frequency of mentally stimulating activities modifies the relationship between cardiovascular reactivity and executive function in old age. The American journal of geriatric psychiatry : official journal of the American Association for Geriatric Psychiatry. 2014;22(11):1210-21.

172. Lin F, Ren P, Wang X, Anthony M, Tadin D, Heffner KL. Cortical thickness is associated with altered autonomic function in cognitively impaired and non-impaired older adults. Journal of Physiology. 2017;595(22):6969-78.

173. Machado-Vidotti HG, Mendes RG, Simoes RP, Castello-Simoes V, Catai AM, Borghi-Silva A. Cardiac autonomic responses during upper versus lower limb resistance exercise in healthy elderly men. Brazilian Journal of Physical Therapy. 2014;18(1):9-18.

174. Mayumi E, Nishitani A, Yuki Y, Nakatsu T, Toyonaga S, Mashima K, et al. Increased blood pressure levels relative to subjective feelings of intensity of exercise determined with the Borg scale in male patients with hypertension. Clinical and experimental hypertension (New York, NY : 1993). 2008;30(3):191-201.

175. Millar PJ, MacDonald MJ, McCartney N. Effects of Isometric Handgrip Protocol on Blood Pressure and Neurocardiac Modulation. International Journal of Sports Medicine. 2011;32(3):174-80.

176. Norcliffe-Kaufmann L, Kaufmann H, Martinez J, Katz Stuart D, Tully L, Reynolds Harmony R. Autonomic Findings in Takotsubo Cardiomyopathy. The American journal of cardiology. 2016;117(2):206-13.

177. Perini R, Milesi S, Fisher NM, Pendergast DR, Veicsteinas A. Heart rate variability during dynamic exercise in elderly males and females. European Journal of Applied Physiology. 2000;82(1-2):8-15.

178. Perpetuini D, Cardone D, Chiarelli Antonio M, Filippini C, Croce P, Zappasodi F, et al. Autonomic impairment in Alzheimer's disease is revealed by complexity analysis of functional thermal imaging signals during cognitive tasks. Physiological measurement. 2019;40(3):034002.

179. Petrofsky J, Prowse M, Remigio W, Raju C, Salcedo S, Sirichotiratana M, et al. The use of an isometric handgrip test to show autonomic damage in people with diabetes. Diabetes Technology and Therapeutics. 2009;11(6):361-8.

180. Piepoli M, Clark AL, Volterrani M, Adamopoulos S, Sleight P, Coats AJS. Contribution of muscle afferents to the hemodynamic, autonomic, and ventilatory responses to exercise in patients with chronic heart failure - Effects of physical training. Circulation. 1996;93(5):940-52.

181. Rodrigues Jhennyfer AL, Santos Bruna C, Medeiros Leonardo H, Gonçalves Thiago CP, Júnior Carlos RB. Effects of Different Periodization Strategies of Combined Aerobic and Strength Training on Heart Rate Variability in Older Women. Journal of strength and conditioning research. 2019.

182. Sales MM, Campbell CSG, Morais PK, Ernesto C, Soares-Caldeira LF, Russo P, et al. Noninvasive method to estimate anaerobic threshold in individuals with type 2 diabetes. Diabetology and Metabolic Syndrome. 2011;3(1).

183. Steptoe A, Feldman PJ, Kunz S, Owen N, Willemsen G, Marmot M. Stress responsivity and socioeconomic status: a mechanism for increased cardiovascular disease risk? European heart journal. 2002;23(22):1757-63.

184. Steptoe A, Kunz-Ebrecht SR, Wright C, Feldman PJ. Socioeconomic position and cardiovascular and neuroendocrine responses following cognitive challenge in old age. Biological Psychology. 2005;69(2):149-66.

185. Steptoe A, Marmot M. Impaired cardiovascular recovery following stress predicts 3-year increases in blood pressure. Journal of Hypertension. 2005;23(3):529-36.

186. Steptoe A, Marmot M. Psychosocial, hemostatic, and inflammatory correlates of delayed poststress blood pressure recovery. Psychosomatic Medicine. 2006;68(4):531-7.

187. Takahashi T, Okada A, Hayano JI, Takeshima N. Responses of heart rate and vagus tone to treadmill walking on land and in water in healthy older adults. J Aging Phys Activ. 2003;11(1):18-26.

188. Virtanen M, Kähönen M, Nieminen T, Karjalainen P, Tarvainen M, Lehtimäki T, et al. Heart rate variability derived from exercise ECG in the detection of coronary artery disease. Physiological measurement. 2007;28(10):1189-200.

189. Wang Norman C, Chicos A, Banthia S, Bergner Daniel W, Lahiri Marc K, Ng J, et al. Persistent sympathoexcitation long after submaximal exercise in subjects with and without coronary artery disease. American journal of physiology Heart and circulatory physiology. 2011;301(3):H912-H20.

190. Wawrzyniak Andrew J, Hamer M, Steptoe A, Endrighi R. Decreased reaction time variability is associated with greater cardiovascular responses to acute stress. Psychophysiology. 2016;53(5):739-48.

191. Wittstein MW, Starobin JM, Schmitz RJ, Shulz SJ, Haran FJ, Rhea CK. Cardiac and gait rhythms in healthy younger and older adults during treadmill walking tasks. Aging Clinical and Experimental Research. 2019;31(3):367-75.

192. Wood R, Maraj B, Lee CM, Reyes R. Short-term heart rate variability during a cognitive challenge in young and older adults. Age and ageing. 2002;31(2):131-5.
