# Supplementary material for: Can Reactivity of Heart Rate Variability Be a Potential Biomarker and Monitoring Tool to Promote Healthy Aging? A Systematic Review With Meta-Analyses
Source: Front Physiol. 2021 Jul 29;12:686129. doi: 10.3389/fphys.2021.686129 (PMC8359814; doi:10.3389/fphys.2021.686129)
Supplement: Supplementary file 2 [file Data_Sheet_1.pdf]

Supplementary File A – Study Protocol and Search Strategy

1 Introduction

1.1 Rationale

Maintaining good cognitive and physical functioning plays a fundamental role in healthy aging and well-being (1-4). Nevertheless, the normal aging process is associated with structural and functional changes in the brain, that are associated with a gradual decline in physical and cognitive abilities, possibly limiting functional abilities of daily life and quality of life (1, 5-11). The functional decline exists on a continuum from healthy aging to pathological states like “mild cognitive impairment” or “dementia” (12-16). In 2015, 46.8 million people were living with dementia (17). The prevalence for mild neurocognitive disorders (mNCD) is more than twice as high as for dementia and ranges between 3 % and 54 % depending on the clinical classification (13, 14, 18-21). The globally growing life expectancy serves as a risk factor for cognitive decline and is accordingly expected to boost incidence and prevalence of neurocognitive disorders including dementia (13, 16, 18, 19, 22-24). A physically or cognitively sedentary lifestyle is another highly prevalent risk factor associated with cognitive decline and increased risk for cognitive impairment (e.g. dementia) in the aging population (25-28). Consequently, the worldwide prevalence of dementia is expected to nearly double every 20 years (12).

To counteract expected cognitive decline in individuals at risk, early detection and prevention of cognitive impairment is crucial (29). Adaptations in lifestyle can endorse a healthier aging process, improve the ageing immune system and slow down cognitive decline (30-33). Recent investigations have shown that non-pharmacological interventions (e.g. changes in lifestyle like physical activity, cognitive stimulation, and/or reductions of vascular risk factors) are powerful protectors for brain atrophy and cognitive decline (34-50). Especially simultaneous cognitive-motor training, often incorporated in exergames, seems to be effective to improve cognition and treating cognitive impairment in both HOA and older adults with mNCD (51-63) while they are, at the same time, able to improve physical (i.e. gait, mobility, activities of daily living) and psychosocial (i.e. motivation, anxiety, well-being, quality of life) (57, 64-70). Nevertheless, despite numerous investigations, it is currently difficult to draw reliable conclusions about the underlying mechanisms and effectiveness of exergames. This is mainly due to the large heterogeneities between studies and inconsistencies in reporting training compounds (52-55, 66-68, 71). Therefore, further investigations are needed “to establish the neurobiological mechanisms and effective components of exergames for cognition and apply this understanding in the development of evidence-based exergame interventions“ (54).

In most training studies, exercise programs are developed and applied based on scientific literature, guidelines, and recommendations in combination with the practical experience of coaches. This approach requires that training programs are prescribed on a group level without information on how the individual has responded to previous training sessions. However, such an approach may lead to success on a group level but might, at the same time, might hide inter-individual differences in training response. The response of (older) individuals to different training modalities (e.g. types and intensities) depends on individual capabilities such as cognitive abilities, physical fitness and motor abilities, as well as demographic characteristics (e.g. age, gender, health status, and the socioemotional status

including motivation, mood, or stress) (72-74). To overcome this limitation of a generalized exercise program offering, suggestions are made towards an individualized approach and application of adapted exercise prescription (75). As an example, Herold et al. (2019) recommend tailoring exercise loads (e.g. by manipulating exercise intensity) to the capabilities of each individual person. Optimally, the exercise parameters are operationalized and adapted to the individual, using specific markers of the internal training load, to provide comparable inter-individual exercise doses (75). This approach is believed allowing further insights into dose-response relationships and to result in more distinct training effects (75, 76).

Exercise dose is defined as „a product of exercise variables (e.g. exercise intensity, exercise duration, type of exercise), training variables (e.g. frequency of training sessions), and the application of training principles and should be operationalized by using a specific marker(s) of internal load" (32, 59, 75, 77-81). The internal training load, hence, is supposed to determine training outcomes (82). Thus, internal training load can be used and should be monitored as a primary parameter to maximize training benefits (82). It can be described as acute individual response (i.e. biomechanical, physiological, and/or psychological response(s)) to training characteristics (external load) and other influencing factors (e.g. climatic conditions, equipment, ground condition) (82).

An optimal measure of internal training load should reflect the "actual psychophysiological response that the body initiates to cope with the requirements elicited by the external load" (82). During cognitive-motor training (e.g. exergaming), the internal training load is mainly influenced by neurocognitive task demands and the physical exercise intensity (83). Comprehensive guidelines and checklists are available that provide classifications of training load regarding physical exercise intensity (e.g. percentage of individual maximal heart rate) (75, 84-87). Therefore, objective monitoring of the relative physical intensity is readily applicable. However, for neurocognitive task demands – that serve as the driving mechanisms for task-specific neuroplasticity (83) – it is difficult to quantify the internal training load. So far, subjective measures as rating of perceived task difficulty or cognitive effort, objective performance measures (e.g. reaction time, accuracy, error rate), physiological measures including cardiac measures (e.g. heart rate, HRV, blood pressure), brain activity (e.g. task-evoked electric brain potentials), and eye activity (e.g. pupillary dilation, blink rate) have been used to assess training load related to neurocognitive task demands (88-90).

According to the 'cardiovascular reactivity hypothesis' (91), real-time monitoring of cardiovascular responses to physical or cognitive stressors provide useful insights into individual psychophysiological response patterns. Effort-related cardiovascular reactivity was reported to be related to cognitive (i.e. executive functioning) as well as physical (i.e. aerobic fitness, exercise performance) capabilities (92, 93). Therefore, monitoring cardiovascular reactivity could be useful to evaluate training adaptations and may additionally be predictive of certain health conditions (92, 94-99). In particular, quantifying beat-to-beat variation of the duration between heart beats (i.e. R-R-Interval), referred to as HRV, has gained considerable interest in diverse fields (100). HRV reflects cardiac autonomic activity (i.e. parasympathetic modulation), which indicates the capability of the autonomic nervous system to respond flexibly to external stimuli and is sensitive to psychophysiological stressors (101-105). In fact, recent systematic reviews have concluded, that real-time HRV is sensitive to task demands (e.g. difficulty, complexity, duration) related to cognitive and mental effort in older adults with and without cognitive impairment (89, 106-110). Furthermore, real-time HRV measures are suitable to distinguish between different intensities and durations of physical exercises (e.g. cardiorespiratory) (111-113).

Taken together, HRV seems to hold promise as a biomarker to monitor internal training load of motor-cognitive training. This would enable individualized training adaptations that, in turn, would allow the

application of the optimal individual exercise dose and progression. To gain a better understanding of the possible applications of HRV reactivity (i.e. the change from resting state to on-task HRV), and to evaluate whether HRV indeed could be used as a proxy measure for internal training load, it is important to establish a comprehensive understanding of moderating variables on HRV reactivity in HOA.

1.2 Objectives

The aim of this systematic review and meta-analysis will be: (a) to summarize relevant literature monitoring HRV reactivity to (1) cognitive exercises, (2) physical exercises, and (3) simultaneous cognitive-motor training in HOA, and; (b) to evaluate key moderating parameters influencing phasic HRV responses during these exercises.

1.2.1 PICOS-scheme

**P**articipants:	healthy older human adults (age $\geq$ 50 years)
**I**nterventions:	(1) physical exercises (e.g. cardiorespiratory exercise, resistance exercises, neuromotor exercise training as defined by the American College of Sports Medicine (ACSM) [82]),<br>(2) cognitive exercise (i.e. cognitive exercises requiring specific cognitive processes (e.g. attentional, executive, memory or visuo-spatial functions)), or<br>(3) simultaneous cognitive-motor training (as defined by [83])
**C**omparison:	resting state
**O**utcomes:	real-time HRV (during exercise/training)
**S**tudy Type:	Controlled Clinical Trials (e.g. randomized controlled trial, non-randomized controlled trial), Epidemiologic Studies (e.g. cohort studies, cross-sectional studies, case-control studies)

1.2.2 Research Questions

What are the key factors affecting phasic HRV responses during (1) physical exercises, (2) cognitive exercises, and (3) simultaneous cognitive-motor training?

2 Methods

2.1 Protocol

This protocol for a systematic review with meta-analysis was developed in accordance with the established guidelines from the „preferred reporting items for systematic review and meta-analysis protocols (PRISMA-P) 2015 statement" [84].

2.2 Information sources

The databases Medline (EBSCO), Embase, Cochrane Library, CINAHL, Psycinfo, Web of Science, Scopus, Pedro will be consulted for publications up to Mai 2020 by a professional librarian of the University of Zurich.

2.3 Search Strategy

In order to identify the key articles for the study objectives, a search strategy was developed based on the PICOS approach and the predefined eligibility criteria. In collaboration with a professional librarian of the University of Zurich, the search strategy was translated into precise search strings for each database. The search string consisted of medical subject headings (MeSH), free text words and Boolean operators. They were constructed to combine predefined terms for population (e.g. adult), intervention (e.g. exercise, training, cognition, cognitive challenge, mental effort, processing speed), outcome (e.g. autonomic nervous system, real-time heart rate variability, cardiac autonomic response, neuro-physiological measure) and study type (e.g. controlled clinical trial, cross-over, epidemiologic study). Within these groups, all terms were combined with OR operators. The search strings were applied without using further filtering options or limits. Please consider Appendix C1 for the complete search strategy and search strings.

2.4 Eligibility Criteria

Controlled clinical trials assessing acute responses in real-time HRV during (1) physical exercises, (2) cognitive exercise, and (3) simultaneous cognitive-motor training will be considered for this systematic review.

Studies will be considered eligible if they fulfill the following criteria:

2.4.1 Inclusion criteria

- Studies written in English
- Subjects: Healthy middle-aged to older adults (≥ 50 years)
- Real-time monitoring of heart rate variability at rest AND during
    (1) physical exercises (e.g. cardiorespiratory exercise, resistance exercises or neuromotor exercise training as defined by the American College of Sports Medicine (ACSM) [82]),
    (2) cognitive exercise (i.e. cognitive tasks requiring specific cognitive processes (e.g. attentional, executive, memory or visuo-spatial functions)), or
    (3) simultaneous cognitive-motor training (as defined by [83])
- heart rate variability measured by validated devices based on electrocardiography, photoplethysmography or pulsoxymetry

2.4.2 Exclusion criteria

- Studies published before 1996
- Reviews, Meta-Analysis, Preliminary Reports, Dissertations, Conference Abstracts, Posters
- HRV measurement do not meet standards of measurement defined by [85]
- No quantitative HRV parameters reported
- Studies focusing on chronic stress, stress management or HRV-biofeedback training

2.5 Study records

2.5.1 Data management and selection process

All records will be systematically screened using the software EPPI-Reviewer Web (Version: 4.11.1.1) [86]. The provided standard coding schema will be adapted to meet all eligibility criteria. The screening and selection process will be pilot tested and executed by two independent reviewers (PM, MT). After removing duplicates, title and abstract of all records will be screened according to the PICOS-criteria. The remaining studies will be screened for eligibility criteria by executing a full-text analysis. Finally, the retrieved results will be matched and discussed for final inclusion by (PM, MT). In case of disagreements, (EdB) will serve as referee. By calculating Cohen's kappa, the strength of the inter-rater agreement of the study selection process will be rated to be poor (0), slight (0.1 – 0.20), fair (0.21 – 0.40), moderate (0.41 – 0.60), substantial (0.61 – 0.80), and almost perfect (0.81 – 1.0) [87-89].

2.5.2 Data collection process

Data will be extracted by two independent reviewers (PM, MT) using the software EPPI-Reviewer Web (Version: 4.11.1.1) [86]. The extracted data will be compared after completion of the data collection process. In case of mismatches, (MA) will inspect the discrepancies and approve the final data set.

2.6 Data items and Outcomes

Information will be extracted from each included trial on: (1) study characteristics (i.e. author, year of publication, study design), (2) demographic characteristics of all study participants (i.e. number, sex, age, population characteristics), (3) type, duration and intensity/complexity of intervention(s), (4) type and duration of resting-state measurements, (5) HRV measurement techniques and device, (6) overview over the controlling of confounders in each study, and (7) outcome measures (all available HR and HRV-parameters) including moderators. Only outcome-measures meeting the standards of HRV measurement will be included [85].

2.7 Risk of bias in individual studies

Methodological quality of all included studies will be assessed by two independent reviewers (PM, MT) using the Quality Assessment Tool for Quantitative Studies (QATQS) of the Effective Public Health Practice Project assessment tool (EPHPP) and its corresponding guidelines [90-92]. This tool was developed to evaluate the methodological quality of a variety of study designs, including randomized and non-randomized controlled trials, as well as observational studies [92]. The EPHPP was judged to be a suitable and reliable tool for systematic reviews and demonstrated content and construct validity [92, 93]. The tool comprises 14 items separated into six components: (1) sample selection, (2) study design, (3) identification and treatment of confounders, (4) blinding of outcome

assessors and of participants, (5) reliability and validity of data collection methods, and (6) withdrawals and dropouts. Each component will be rated strong, moderate, or weak according to objective criteria of the standardized guidelines and dictionary. The overall methodological quality of each study will be considered strong (i.e. no weak ratings), moderate (i.e. one weak rating) or weak (i.e. two or more weak ratings) [90-92]. In case of disagreements, RK will serve as referee.

Confounders that will be considered in the analysis of methodological quality [94]:

(1) Age and gender
(2) Smoking
(3) Habitual levels of alcohol consumption
(4) Weight, height and waist-to-hip ratio
(5) Cardioactive medication, such as antidepressant, antipsychotic or antihypertensive
(6) Oral contraceptive intake for female participants
(7) Follow a normal sleep routine the day before the experiment, record the typical bedtime and typical waking time
(8) No intense physical training the day before the experiment
(9) No meal the last 2 h before the experiment
(10) No coffee – or caffeinated drinks such as energizing drinks – or in the 2 h before the experiment
(11) Ask if they need to use the bathroom before the experiment begins
(12) No alcohol for 24h prior to the experiment

2.8 Data Synthesis

Only studies with moderate to strong methodological qualities and outcome-measures meeting the standards of HRV measurement evaluated by a validated device will be considered for the quantitative synthesis [85, 95]. Outcome measures reflecting mainly cardiac vagal tone were included in hierarchical order: (1) RMSSD, (2) pNN50, (3) HFnu, (4) HF, and (5) SD1 (119, 120, 125, 130-132). Both, absolute and log-transformed values, were synthesized according to the Cochrane guidelines (133).

A pooled estimate will be calculated for HRV reactivity by conducting a meta-analysis in R (R Version R 3.6.2 GUI 1.70 El Capitan build (7735) (© The R Foundation)) in line with RStudio Version 1.2.5033 (RStudio, Inc.) [99] using a fixed-effects model of the 'metaphor' package [100] to calculate standardized mean differences (hedge's g) [100] and 95 % confidence intervals between HRV on-task and resting HRV. Hedge's g will be used due to its benefit for correcting biases found in small sample sizes. Level of significance will be set to $p \leq 0.05$ and effect sizes will be interpreted to be small ($d < 0.5$), medium ($0.5 \leq d < 0.8$) or large ($d > 0.8$) [101].

A planned subgroup analysis will be performed for cognitive tasks, physical exercises (i.e. cardiorespiratory exercises and resistance training) and cognitive-motor training. Furthermore, to evaluate the effect of age and cognitive function, further planned subgroup analyses will be performed to compare resting- and on-task values of HRV as well as HRV reactivity (i.e. difference between HRV on-task and resting HRV) for all available intervention with (1) healthy adults (i.e. ≤ 50 years), and (2) older adults with cognitive impairment (i.e. defined as a Montreal Cognitive Assessment (MoCA) score below 26, which is used as a cut-off point to detect mild cognitive impairment [102]). Each subgroup analysis will be performed by using random-effects model comparisons.

2.9 Risk of bias across studies

Possible sources of heterogeneity among trials will be investigated by using Cochrane Q in line with I^2 statistics. In case of significant heterogeneity, indicated by significant Q-statistics ($p < 0.05$), random-effect models will be employed [103]. To detect possible publication bias, funnel plots (i.e. standard error) will be assessed both visually and formally with Egger's test [104, 105]. When publication biases will be indicated (i.e. Egger's regression test: $p < 0.1$), sensitivity analyses will be performed by (1) comparing fixed- and random-effect models, and (2) applying a sensitivity analysis based on the trim and fill method for random-effects models. The trim and fill method redresses funnel plot asymmetries by adjusting the point estimated of the pooled effect sizes and 95 % confidence intervals for missing studies [106].

3 References

1. Organization, W.H., World report on ageing and health. 2015: World Health Organization.
2. Reuter-Lorenz, P.A., S.B. Festini, and T.K. Jantz, Executive Functions and Neurocognitive Aging, in Handbook of the Psychology of Aging (Eighth Edition), K.W. Schaie and S.L. Willis, Editors. 2016, Academic Press: San Diego. p. 245-262.
3. Zanjari, N., et al., Successful aging as a multidimensional concept: An integrative review. Med J Islam Repub Iran, 2017. 31: p. 100.
4. Yam, A. and M. Marsiske, Cognitive longitudinal predictors of older adults' self-reported IADL function. J Aging Health, 2013. 25(8 Suppl): p. 163s-85s.
5. Harada, C.N., M.C. Natelson Love, and K.L. Triebel, Normal cognitive aging. Clinics in geriatric medicine, 2013. 29(4): p. 737-752.
6. Lindbergh, C.A., R.K. Dishman, and L.S. Miller, Functional Disability in Mild Cognitive Impairment: A Systematic Review and Meta-Analysis. Neuropsychol Rev, 2016. 26(2): p. 129-59.
7. Prince, M., et al., World Alzheimer Report 2015. The Global Impact of Dementia. An Analysis of Prevalence, Incidence, Cost and Trends. 2015.
8. Janelidze, M. and N. Botchorishvili, Mild Cognitive Impairment. Alzheimer's Disease: The 21st Century Challenge, 2018. 91.
9. Levine, M.E., A. Harrati, and E.M. Crimmins, Predictors and Implications of Accelerated Cognitive Aging. Biodemography Soc Biol, 2018. 64(2): p. 83-101.
10. Kontis, V., et al., Future life expectancy in 35 industrialised countries: projections with a Bayesian model ensemble. Lancet, 2017. 389(10076): p. 1323-1335.
11. Guthold, R., et al., Worldwide trends in insufficient physical activity from 2001 to 2016: a pooled analysis of 358 population-based surveys with 1· 9 million participants. The Lancet Global Health, 2018. 6(10): p. e1077-e1086.
12. Licher, S., et al., Lifetime risk and multimorbidity of non-communicable diseases and disease-free life expectancy in the general population: A population-based cohort study. PLoS Med, 2019. **16**(2): p. e1002741.
13. Morley, J.E., et al., *Brain health: the importance of recognizing cognitive impairment: an IAGG consensus conference.* Journal of the American Medical Directors Association, 2015. **16**(9): p. 731-739.
14. Nieman, D.C. and L.M. Wentz, *The compelling link between physical activity and the body's defense system.* Journal of Sport and Health Science, 2019. **8**(3): p. 201-217.
15. Bosnes, I., et al., Lifestyle predictors of successful aging: A 20-year prospective HUNT study. PLOS ONE, 2019. **14**(7): p. e0219200.
16. Erickson, K.I., et al., Physical Activity, Cognition, and Brain Outcomes: A Review of the 2018 Physical Activity Guidelines. Med Sci Sports Exerc, 2019. **51**(6): p. 1242-1251.
17. Daskalopoulou, C., et al., Physical activity and healthy ageing: A systematic review and meta-analysis of longitudinal cohort studies. Ageing Research Reviews, 2017. **38**: p. 6-17.

18. Liang, J.H., et al., Comparison of multiple interventions for older adults with Alzheimer disease or mild cognitive impairment: A PRISMA-compliant network meta-analysis. Medicine (Baltimore), 2018. **97**(20): p. e10744.

19. Brasure, M., et al., Physical Activity Interventions in Preventing Cognitive Decline and Alzheimer-Type Dementia: A Systematic Review. Ann Intern Med, 2018. **168**(1): p. 30-38.

20. Sanders, L.M.J., et al., Dose-response relationship between exercise and cognitive function in older adults with and without cognitive impairment: A systematic review and meta-analysis. PLoS One, 2019. **14**(1): p. e0210036.

21. Nguyen, L., K. Murphy, and G. Andrews, Cognitive and neural plasticity in old age: A systematic review of evidence from executive functions cognitive training. Ageing Res Rev, 2019. **53**: p. 100912.

22. Gomes-Osman, J., et al., Exercise for cognitive brain health in aging: A systematic review for an evaluation of dose. Neurol Clin Pract, 2018. **8**(3): p. 257-265.

23. Butler, M., et al., Does Cognitive Training Prevent Cognitive Decline?: A Systematic Review. Ann Intern Med, 2018. **168**(1): p. 63-68.

24. Mewborn, C.M., C.A. Lindbergh, and L. Stephen Miller, Cognitive Interventions for Cognitively Healthy, Mildly Impaired, and Mixed Samples of Older Adults: A Systematic Review and Meta-Analysis of Randomized-Controlled Trials. Neuropsychol Rev, 2017. **27**(4): p. 403-439.

25. Hill, N.T., et al., Computerized Cognitive Training in Older Adults With Mild Cognitive Impairment or Dementia: A Systematic Review and Meta-Analysis. Am J Psychiatry, 2017. **174**(4): p. 329-340.

26. Joubert, C. and H. Chainay, Aging brain: the effect of combined cognitive and physical training on cognition as compared to cognitive and physical training alone - a systematic review. Clin Interv Aging, 2018. **13**: p. 1267-1301.

27. Lauenroth, A., A.E. Ioannidis, and B. Teichmann, Influence of combined physical and cognitive training on cognition: a systematic review. BMC Geriatr, 2016. **16**: p. 141.

28. Eggenberger, P., et al., Does multicomponent physical exercise with simultaneous cognitive training boost cognitive performance in older adults? A 6-month randomized controlled trial with a 1-year follow-up. Clin Interv Aging, 2015. **10**: p. 1335-49.

29. Sokolov, A.A., A. Collignon, and M. Bieler-Aeschlimann, Serious video games and virtual reality for prevention and neurorehabilitation of cognitive decline because of aging and neurodegeneration. Curr Opin Neurol, 2020. **33**(2): p. 239-248.

30. Stojan, R. and C. Voelcker-Rehage, A Systematic Review on the Cognitive Benefits and Neurophysiological Correlates of Exergaming in Healthy Older Adults. J Clin Med, 2019. **8**(5).

31. Moreno, A., et al., A systematic review of the use of virtual reality and its effects on cognition in individuals with neurocognitive disorders. Alzheimers Dement (N Y), 2019. **5**: p. 834-850.

32. Stanmore, E., et al., The effect of active video games on cognitive functioning in clinical and non-clinical populations: A meta-analysis of randomized controlled trials. Neurosci Biobehav Rev, 2017. **78**: p. 34-43.

33. Oh, Y. and S.J.P.o.M.P. Yang, *Defining exergames & exergaming.* 2010: p. 1-17.

34. Theill, N., et al., Effects of simultaneously performed cognitive and physical training in older adults. BMC Neurosci, 2013. **14**(1): p. 103.

35. Levin, O., Y. Netz, and G. Ziv, The beneficial effects of different types of exercise interventions on motor and cognitive functions in older age: a systematic review. European review of aging and physical activity : official journal of the European Group for Research into Elderly and Physical Activity, 2017. **14**: p. 20-20.

36. Schoene, D., et al., The effect of interactive cognitive-motor training in reducing fall risk in older people: a systematic review. BMC geriatrics, 2014. **14**: p. 107-107.

37. Knols, R.H., et al., Exergames for Patients in Acute Care Settings: Systematic Review of the Reporting of Methodological Quality, FITT Components, and Program Intervention Details. Games for Health Journal, 2016. **5**(3): p. 224-235.

38. Hautala, A.J., et al., *Cardiovascular autonomic function correlates with the response to aerobic training in healthy sedentary subjects.* American Journal of Physiology-Heart and Circulatory Physiology, 2003. **285**(4): p. H1747-H1752.

39. Bouchard, C. and T. Rankinen, *Individual differences in response to regular physical activity.* Med Sci Sports Exerc, 2001. **33**(6 Suppl): p. S446-51; discussion S452-3.

40. Vollaard, N.B., et al., Systematic analysis of adaptations in aerobic capacity and submaximal energy metabolism provides a unique insight into determinants of human aerobic performance. J Appl Physiol (1985), 2009. **106**(5): p. 1479-86.

41. Herold, F., et al., Dose–Response Matters! – A Perspective on the Exercise Prescription in Exercise–Cognition Research. Frontiers in Psychology, 2019. **10**(2338).

42. Ross, R., et al., *Precision exercise medicine: understanding exercise response variability.* British journal of sports medicine, 2019. **53**(18): p. 1141-1153.

43. Falck, R.S., et al., Impact of exercise training on physical and cognitive function among older adults: a systematic review and meta-analysis. Neurobiology of aging, 2019.

44. Etnier, J.L., E.S. Drollette, and A.B. Slutsky, *Physical activity and cognition: A narrative review of the evidence for older adults.* Psychology of Sport and Exercise, 2019. **42**: p. 156-166.

45. Cabral, D.F., et al., Exercise for brain health: an investigation into the underlying mechanisms guided by dose. Neurotherapeutics, 2019: p. 1-20.

46. Northey, J.M., et al., Exercise interventions for cognitive function in adults older than 50: a systematic review with meta-analysis. Br J Sports Med, 2018. **52**(3): p. 154-160.

47. Wasfy, M.M. and A.L. Baggish, *Exercise Dose in Clinical Practice.* Circulation, 2016. **133**(23): p. 2297-2313.

48. Impellizzeri, F., S. Marcora, and A. Coutts, *Internal and External Training Load: 15 Years On.* International Journal of Sports Physiology and Performance, 2019. **14**: p. 1-4.

49. Halson, S.L., *Monitoring training load to understand fatigue in athletes.* Sports medicine (Auckland, N.Z.), 2014. **44 Suppl 2**(Suppl 2): p. S139-S147.

50. Bourdon, P.C., et al., *Monitoring Athlete Training Loads: Consensus Statement.* Int J Sports Physiol Perform, 2017. **12**(Suppl 2): p. S2161-s2170.

51. Burgess, D.J., The Research Doesn't Always Apply: Practical Solutions to Evidence-Based Training-Load Monitoring in Elite Team Sports. Int J Sports Physiol Perform, 2017. **12**(Suppl 2): p. S2136-s2141.

52. Vanrenterghem, J., et al., Training Load Monitoring in Team Sports: A Novel Framework Separating Physiological and Biomechanical Load-Adaptation Pathways. Sports Med, 2017. **47**(11): p. 2135-2142.

53. McLaren, S.J., et al., The Relationships Between Internal and External Measures of Training Load and Intensity in Team Sports: A Meta-Analysis. Sports Med, 2018. **48**(3): p. 641-658.

54. Medicine, A.C.o.S., et al., *ACSM's Guidelines for Exercise Testing and Prescription. 10th*. 2017, Philadelphia: Lippincott Williams & Wilkins.

55. Slade, S.C., et al., *Consensus on Exercise Reporting Template (CERT): explanation and elaboration statement.* British journal of sports medicine, 2016. **50**(23): p. 1428-1437.

56. Hoffman, J., Physiological aspects of sport training and performance. 2014: Human Kinetics.

57. Brünken, R., T. Seufert, and F. Paas, *Measuring Cognitive Load*, in *Cognitive Load Theory*, J.L. Plass, R. Brünken, and R. Moreno, Editors. 2010, Cambridge University Press: Cambridge. p. 181-202.

58. Obrist, P.A., *Cardiovascular psychophysiology: A perspective*. 2012: Springer Science & Business Media.

59. D'Agosto, T., et al., Cardiac autonomic responses at onset of exercise: effects of aerobic fitness. Int J Sports Med, 2014. **35**(10): p. 879-85.

60. Silvestrini, N., Psychological and neural mechanisms associated with effort-related cardiovascular reactivity and cognitive control: An integrative approach. Int J Psychophysiol, 2017. **119**: p. 11-18.

61. Borresen, J. and M.I. Lambert, Autonomic control of heart rate during and after exercise : measurements and implications for monitoring training status. Sports Med, 2008. **38**(8): p. 633-46.

62. Lovallo, W.R., Cardiovascular reactivity: mechanisms and pathways to cardiovascular disease. Int J Psychophysiol, 2005. **58**(2-3): p. 119-32.

63. Phillips, A.C., Blunted as well as exaggerated cardiovascular reactivity to stress is associated with negative health outcomes 1. Japanese Psychological Research, 2011. **53**(2): p. 177-192.

64. Phillips, A.C., Blunted cardiovascular reactivity relates to depression, obesity, and self-reported health. Biological Psychology, 2011. **86**(2): p. 106-113.

65. Treiber, F.A., et al., Cardiovascular reactivity and development of preclinical and clinical disease states. Psychosom Med, 2003. **65**(1): p. 46-62.

66. Schiweck, C., et al., Heart rate and high frequency heart rate variability during stress as biomarker for clinical depression. A systematic review. Psychological Medicine, 2019. **49**(2): p. 200-211.

67. Thayer, J.F., *Heart Rate Variability: A Neurovisceral Integration Model.* In R.S. Larry (Ed.), Encyclopedia of neuroscience, 2009: p. 1041-1047.

68. Singh, N., et al., Heart Rate Variability: An Old Metric with New Meaning in the Era of using mHealth Technologies for Health and Exercise Training Guidance. Part One: Physiology and Methods. Arrhythm Electrophysiol Rev, 2018. **7**(3): p. 193-198.

69. Hillmert, M., et al., Psychophysiological strain correlates during cognitive workload: A laboratory study using EEG and ECG. Zentralblatt fur Arbeitsmedizin, Arbeitsschutz und Ergonomie, 2020.

70. Giannakakis, G., et al., *Review on psychological stress detection using biosignals.* IEEE Transactions on Affective Computing, 2019.

71. Rajendra Acharya, U., et al., *Heart rate variability: a review.* Med Biol Eng Comput, 2006. **44**(12): p. 1031-51.

72. Forte, G., F. Favieri, and M. Casagrande, *Heart Rate Variability and Cognitive Function: A Systematic Review.* Frontiers in neuroscience, 2019. **13**: p. 710-710.

73. Hughes, A.M., et al., *Cardiac Measures of Cognitive Workload: A Meta-Analysis.* Human factors, 2019. **61**(3): p. 393-414.

74. Ranchet, M., et al., Cognitive workload across the spectrum of cognitive impairments: A systematic review of physiological measures. Neurosci Biobehav Rev, 2017. **80**: p. 516-537.

75. Castaldo, R., et al., Acute mental stress assessment via short term HRV analysis in healthy adults: A systematic review with meta-analysis. Biomedical Signal Processing and Control, 2015. **18**: p. 370-377.

76. Kim, H.-G., et al., Stress and Heart Rate Variability: A Meta-Analysis and Review of the Literature. Psychiatry investigation, 2018. **15**(3): p. 235-245.

77. Charles, R.L. and J. Nixon, Measuring mental workload using physiological measures: A systematic review. Applied ergonomics, 2019. **74**: p. 221-232.

78. Tao, D., et al., *A Systematic Review of Physiological Measures of Mental Workload.* International Journal of Environmental Research and Public Health, 2019. **16**: p. 2716.

79. Michael, S., K.S. Graham, and G.M.O. Davis, Cardiac Autonomic Responses during Exercise and Post-exercise Recovery Using Heart Rate Variability and Systolic Time Intervals-A Review. Front Physiol, 2017. **8**: p. 301.

80. Dong, J.-G., *The role of heart rate variability in sports physiology.* Experimental and therapeutic medicine, 2016. **11**(5): p. 1531-1536.

81. Gronwald, T. and O. Hoos, Correlation properties of heart rate variability during endurance exercise: A systematic review. Annals of Noninvasive Electrocardiology, 2020. **25**(1): p. e12697.

82. Garber, C.E., et al., American College of Sports Medicine position stand. Quantity and quality of exercise for developing and maintaining cardiorespiratory, musculoskeletal, and neuromotor fitness in apparently healthy adults: guidance for prescribing exercise. Med Sci Sports Exerc, 2011. **43**(7): p. 1334-59.

83. Herold, F., et al., Thinking While Moving or Moving While Thinking – Concepts of Motor-Cognitive Training for Cognitive Performance Enhancement. Frontiers in Aging Neuroscience, 2018. **10**(228).

84. Moher, D., et al., Preferred reporting items for systematic review and meta-analysis protocols (PRISMA-P) 2015 statement. Systematic reviews, 2015. **4**(1): p. 1.

85. Malik, M., Heart rate variability: Standards of measurement, physiological interpretation, and clinical use. Circulation, 1996. **93**: p. 1043-1065.

86. Thomas, J., J. Brunton, and S. Graziosi, EPPI-Reviewer 4.0: software for research synthesis. EPPI-Centre Software. London: Social Science Research Unit. Institute of education, University of london, 2010.

87. Landis, J.R. and G.G. Koch, *The Measurement of Observer Agreement for Categorical Data.* Biometrics, 1977. **33**(1): p. 159-174.

88. Cohen, J., *A coefficient of agreement for nominal scales.* Educational and psychological measurement, 1960. **20**(1): p. 37-46.

89. McHugh, M.L., *Interrater reliability: the kappa statistic.* Biochemia medica, 2012. **22**(3): p. 276-282.

90. Thomas, B., et al. Quality assessment tool for quantitative studies: the Effective Public Health Practice Project (EPHPP). 2008.

91. Thomas, B., et al. Quality assessment tool for quantitative studies dictionary: the Effective Public Health Practice Project (EPHPP). 2008.

92. Thomas, B.H., et al., A process for systematically reviewing the literature: providing the research evidence for public health nursing interventions. Worldviews Evid Based Nurs, 2004. **1**(3): p. 176-84.

93. Deeks, J., et al., Evaluating non-randomised intervention studies. Health Technol Assess (Winchester, England). 7(27):iii–x, 2003: p. 1-173.

94. Laborde, S., E. Mosley, and J.F. Thayer, Heart Rate Variability and Cardiac Vagal Tone in Psychophysiological Research - Recommendations for Experiment Planning, Data Analysis, and Data Reporting. Frontiers in psychology, 2017. **8**: p. 213-213.

95. Shaffer, F. and J.P. Ginsberg, *An Overview of Heart Rate Variability Metrics and Norms.* Frontiers in public health, 2017. **5**: p. 258-258.

96. Alvares, G.A., et al., Autonomic nervous system dysfunction in psychiatric disorders and the impact of psychotropic medications: a systematic review and meta-analysis. Journal of psychiatry & neuroscience : JPN, 2016. **41**(2): p. 89-104.

97. , T.F.o.t.E.S.o.C.t.N.A.S.o.P.E., *Heart Rate Variability.* Circulation, 1996. **93**(5): p. 1043-1065.

98. Julian PT Higgins, T.L., Jonathan J Deeks, Choosing effect measures and computing estimates of effect, in Cochrane Handbook for Systematic Reviews of Interventions. 2019. p. 143-176.

99. Team, R.C., *R: A Language and Environment for Statistical Computing*. 2019, R Foundation for Statistical Computing.

100. Viechtbauer, W., *Conducting meta-analyses in R with the metafor package.* Journal of statistical software, 2010. **36**(3): p. 1-48.

101. Cohen, J., Statistical power analysis for the behavioral sciences. 1988: Routledge.

102. Nasreddine, Z.S., et al., *The Montreal Cognitive Assessment, MoCA: A Brief Screening Tool For Mild Cognitive Impairment.* Journal of the American Geriatrics Society, 2005. **53**(4): p. 695-699.

103. Higgins, J.P. and S.G. Thompson, *Quantifying heterogeneity in a meta-analysis.* Statistics in medicine, 2002. **21**(11): p. 1539-1558.

104. Egger, M., et al., Bias in meta-analysis detected by a simple, graphical test. Bmj, 1997. **315**(7109): p. 629-634.

105. Sterne, J.A.C. and M. Egger, *Funnel plots for detecting bias in meta-analysis: Guidelines on choice of axis.* Journal of Clinical Epidemiology, 2001. **54**(10): p. 1046-1055.

106. Duval, S. and R. Tweedie, Trim and fill: a simple funnel-plot–based method of testing and adjusting for publication bias in meta-analysis. Biometrics, 2000. **56**(2): p. 455-463.

107. Manera, V., et al., Recommendations for the Use of Serious Games in Neurodegenerative Disorders: 2016 Delphi Panel. Front Psychol, 2017. **8**: p. 1243.

3.1 Appendix C1: Search Strategy

3.2 Step 1: Define search terms

3.2.1 Mesh-Terms:

**P**atient:	„Adult", and corresponding subgroups
**I**ntervention:	(a) „Exercise", „Sports", and corresponding subgroups<br>(b) „Cognition", "Attention", "Awareness", "Learning", "Executive Functions", "Memory" and corresponding subgroups<br>(c)
**C**omparison:	none
**O**utcome:	„Autonomic Nervous System", and corresponding subgroups
**S**tudy Type:	"Controlled Clinical Trial", "Epidemiologic Studies", and corresponding subgroups

3.2.2 Text words:

**P**atient:	adults
**I**ntervention:	(a) exercis*, training*, endurance*, "physical conditioning*", HIIT, strength*, running*, jogging*, swimming*, walking*, sport*,<br>(b) cognition, cognitive workload*, cognitive task*, cognitive performance*, cognitive processing*, cognitive overload*, cognitive effort*, cognitive load*, cognitive challenge*, cognitive stress*, cognitive demand*, mental workload*, mental task*, mental performance*, mental processing*, mental overload*, mental effort*, mental load*, mental challenge*, mental stress*, mental demand*, attention*, awareness*, learning*, "processing speed*", "executive function*", "visuo-spatial abilit*", memory,<br>(c) exergam*, "virtual reality", VR, active videogame*
**C**omparison:	none
**O**utcome:	HRV, "beat-to-beat", "heart rate variability", cardiac autonomic response, autonomic flexib*, autonomic response*, "cardiovagal activit*", "neuro-physiological measure*", "real-time", "short-term" acute measure*, acute response*, immediate*, while, perform*, execut*
**S**tudy Type:	control* group*, control* stud*, control* trial*, random*, assign*, allocat*, volunteer*, crossover, "cross-over", participant*

3.3 Step 2: Define relevant databases

- Medline (EBSCO)
- Embase
- Cochrane Library
- CINAHL
- Psycinfo
- Web of Science
- Scopus, Pedro

3.4 Step 3: Define structure of search strings

- o Link population, intervention, outcome and study type using the Boolean operator "OR"
- o Limit outcomes to real-time measurements of HRV:
  - o Term "acute" has to be in proximity to the terms "measure*", "response*"
  - o Terms "cardiac", "flexib*", "response*" hve to be in proximity to the term "autonomic"
- o Link search terms within these groups using the Boolean operator „OR"
- o Exclude animals, infants, children, adolescents

3.5 Step 4: Synthesize strategy into search strings for each database

Table 1: Search strings for each database.

Database	Search String	No. of articles retrieved
Medline (EBSCO)	((MH "Autonomic Nervous System+") OR TI ((hrv OR "beat-to-beat" OR "heart rate variability" OR ((cardiac OR flexib* OR response*) N3 autonomic) OR "cardiovagal activit*" OR "neuro-physiological measure*")) OR AB ((hrv OR "beat-to-beat" OR "heart rate variability" OR ((cardiac OR flexib* OR response*) N3 autonomic) OR "cardiovagal activit*" OR "neuro-physiological measure*"))) AND (TI (("real-time" OR "short-term" OR (acute N5 (measure* OR response*)) OR immediate* OR while OR perform* OR execut*)) OR AB (("real-time" OR "short-term" OR (acute N5 (measure* OR response*)) OR immediate* OR while OR perform* OR execut*))) AND (((MH "Exercise+") OR (MH "Sports+") OR (MH "Cognition+") OR (MH "Attention+") OR (MH "Awareness") OR (MH "Learning+") OR (MH "Executive Function") OR (MH "Memory+")) OR TI ((exercis* OR training* OR endurance* OR "physical conditioning*" OR HIIT OR strength* OR running* OR jogging* OR swimming* OR walking* OR sport* OR cognition OR ((cognitive OR mental) N3 (workload* OR task* OR performance* OR processing* OR overload* OR effort* OR load* OR challenge* OR stress* OR demand*)) OR attention* OR awareness* OR learning* OR "processing speed*" OR "executive function*" OR "visuo-spatial abilit*" OR memory OR exergam* OR "virtual reality" OR VR OR (active N3 videogame*))) OR AB ((exercis* OR training* OR endurance* OR "physical conditioning*" OR HIIT OR strength* OR running* OR jogging* OR swimming* OR walking* OR sport* OR cognition OR ((cognitive OR mental) N3 (workload* OR task* OR performance* OR processing* OR overload* OR effort* OR load* OR challenge* OR stress* OR demand*)) OR attention* OR awareness* OR learning* OR "processing speed*" OR "executive function*" OR "visuo-spatial abilit*" OR memory OR exergam* OR "virtual reality" OR VR OR (active N3 videogame*)))) AND (((MH "Controlled Clinical Trial+") OR (MH "Epidemiologic Studies+")) OR TI (((control* N3 (group* OR stud* OR trial*))) OR (random* OR assign* OR allocat* OR volunteer* OR crossover OR "cross- over" OR participant*)) OR AB (((control* N3 (group* OR stud* OR trial*))) OR (random* OR assign* OR allocat* OR volunteer* OR crossover OR "cross-over" OR participant*))) NOT (((MH "Animals+") NOT (MH "Humans"))) NOT ((((MH "Child+") OR (MH "Infant+") OR (MH "Adolescent")) NOT (MH "Adult+")))	2,293 results (May 5, 2020)
Embase	'heart rate variability'/exp OR hrv:ti,ab OR 'beat-to-beat':ti,ab OR 'heart rate variability':ti,ab OR (((cardiac OR flexib* OR response*) NEAR/3 autonomic):ti,ab) OR ('cardiovagal activit*':ti,ab OR 'neuro-physiological measure*':ti,ab) AND ('real-time':ti,ab OR 'short-term':ti,ab OR ((acute NEAR/5 (measure* OR response*)):ti,ab) OR immediate*:ti,ab OR while:ti,ab OR perform*:ti,ab OR execut*:ti,ab) AND ('exercise'/exp OR 'sport'/exp OR 'cognition'/exp OR 'attention'/exp OR 'awareness'/exp OR 'learning'/exp OR 'executive function'/exp OR 'visuospatial ability'/exp OR 'memory'/exp OR exercis*:ti,ab OR training*:ti,ab OR endurance*:ti,ab OR 'physical conditioning*':ti,ab OR hiit:ti,ab OR strength*:ti,ab OR running*:ti,ab OR jogging*:ti,ab OR swimming*:ti,ab OR walking*:ti,ab OR sport*:ti,ab OR cognition:ti,ab OR (((cognitive OR mental) NEAR/3 (workload* OR task* OR performance* OR processing* OR overload* OR effort* OR load* OR challenge* OR stress* OR demand*)):ti,ab) OR attention*:ti,ab OR awareness*:ti,ab OR learning*:ti,ab OR 'processing speed*':ti,ab OR 'executive function*':ti,ab OR 'visuo-spatial abilit*':ti,ab OR memory:ti,ab OR exergam*:ti,ab OR 'virtual reality':ti,ab OR vr:ti,ab OR ((active NEAR/3 videogame*):ti,ab)) AND ('controlled study'/exp OR 'controlled clinical trial'/exp OR ((control* NEAR/3 (group* OR stud* OR trial*)):ti,ab) OR random*:ti,ab OR assign*:ti,ab OR allocat*:ti,ab OR volunteer*:ti,ab OR crossover:ti,ab OR 'cross-over':ti,ab OR participant*:ti,ab) NOT ([animals]/lim NOT [humans]/lim) NOT (([infant]/lim OR [child]/lim OR [adolescent]/lim) NOT ([adult]/lim OR [aged]/lim))	3,677 results (May 5, 2020)
Cochrane Library	((hrv OR "beat-to-beat" OR "heart rate variability" OR ((cardiac OR flexib* OR response*) NEAR/3 autonomic) OR "cardiovagal activit*" OR "neuro-physiological measure*"):ti,ab,kw) AND (("real-time" OR "short-term" OR (acute NEAR/5 (measure* OR response*)) OR immediate* OR while OR perform* OR execut*):ti,ab,kw) AND ((exercis* OR training* OR endurance* OR "physical conditioning*" OR HIIT OR strength* OR running* OR jogging* OR swimming* OR walking* OR sport* OR cognition OR ((cognitive OR mental) NEAR/3 (workload* OR task* OR performance* OR processing* OR overload* OR effort* OR load* OR challenge* OR stress* OR demand*)) OR attention* OR awareness* OR learning* OR "processing speed*" OR "executive function*" OR "visuo-spatial abilit*" OR memory OR exergam* OR "virtual reality" OR VR OR (active NEAR/3 videogame*)):ti,ab,kw)	1,077 results (May 5, 2020)

Psycinfo	(DE "Heart Rate Variability OR TI ((hrv OR "beat-to-beat" OR "heart rate variability" OR ((cardiac OR flexib* OR response*) N3 autonomic) OR "cardiovagal activit*" OR "neuro-physiological measure*")) OR AB ((hrv OR "beat-to-beat" OR "heart rate variability" OR ((cardiac OR flexib* OR response*) N3 autonomic) OR "cardiovagal activit*" OR "neuro-physiological measure*"))) AND (TI (("real-time" OR "short-term" OR (acute N5 (measure* OR response*)) OR immediate* OR while OR perform* OR execut*)) OR AB (("real-time" OR "short-term" OR (acute N5 (measure* OR response*)) OR immediate* OR while OR perform* OR execut*))) AND (TI ((exercis* OR training* OR endurance* OR "physical conditioning*" OR HIIT OR strength* OR running* OR jogging* OR swimming* OR walking* OR sport* OR cognition OR ((cognitive OR mental) N3 (workload* OR task* OR performance* OR processing* OR overload* OR effort* OR load* OR challenge* OR stress* OR demand*)) OR attention* OR awareness* OR learning* OR "processing speed*" OR "executive function*" OR "visuo-spatial abilit*" OR memory OR exergam* OR "virtual reality" OR VR OR (active N3 videogame*))) OR AB ((exercis* OR training* OR endurance* OR "physical conditioning*" OR HIIT OR strength* OR running* OR jogging* OR swimming* OR walking* OR sport* OR cognition OR ((cognitive OR mental) N3 (workload* OR task* OR performance* OR processing* OR overload* OR effort* OR load* OR challenge* OR stress* OR demand*)) OR attention* OR awareness* OR learning* OR "processing speed*" OR "executive function*" OR "visuo-spatial abilit*" OR memory OR exergam* OR "virtual reality" OR VR OR (active N3 videogame*))) OR (DE "Exercise" OR DE "Sports" OR DE "Athletes" OR DE "Swimming" DE "Cognition" DE "Attention" OR DE "Learning" OR DE "Executive Function" OR DE "Cognitive Control" OR DE "Memory")) AND (TI (((control* N3 (group* OR stud* OR trial*))) OR (random* OR assign* OR allocat* OR volunteer* OR crossover OR "cross- over" OR participant*)) OR AB (((control* N3 (group* OR stud* OR trial*))) OR (random* OR assign* OR allocat* OR volunteer* OR crossover OR "cross-over" OR participant*)))	598 results (May 5, 2020)
Web of Science	((TOPIC: ((hrv OR "beat-to-beat" OR "heart rate variability" OR ((cardiac OR flexib* OR response*) NEAR/3 autonomic) OR "cardiovagal activit*" OR "neuro-physiological measure*") AND ("real-time" OR "short-term" OR (acute NEAR/5 (measure*)) OR immediate* OR while OR perform* OR execut*))) AND (TOPIC: ((exercise* OR training* OR endurance* OR "physical conditioning*" OR HIIT OR strength* OR running* OR jogging* OR swimming* OR walking* OR sport* OR cognition OR ((cognitive OR mental) NEAR/3 (workload* OR task* OR performance* OR processing* OR overload* OR effort* OR load* OR challenge* OR stress* OR demand*)) OR attention* OR awareness* OR learning* OR "processing speed*" OR "executive function*" OR "visuo-spatial ability*" OR memory OR exergame* OR *virtual reality" OR VR OR (active NEAR/3 videogame*)))) AND (TOPIC: (((control* NEAR/3 (group* OR stud* OR trial*))) OR (random* OR assign* OR allocate* OR volunteer* OR crossover OR "cross-over" OR participant*)))	2,352 results (May 5, 2020)
Scopus	((TITLE-ABS-KEY ((hrv OR "beat-to-beat" OR "heart rate variability" OR ((cardiac OR flexib* OR response*) NEAR/3 autonomic) OR "cardiovagal activit*" OR "neuro-physiological measure*") AND ("real-time" OR "short-term" OR (acute NEAR/5 (measure*)) OR immediate* OR while OR perform* OR execut*))) AND (TITLE-ABS-KEY ((exercise* OR training* OR endurance* OR "physical conditioning*" OR HIIT OR strength* OR running* OR jogging* OR swimming* OR walking* OR sport* OR cognition OR ((cognitive OR mental) NEAR/3 (workload* OR task* OR performance* OR processing* OR overload* OR effort* OR load* OR challenge* OR stress* OR demand*)) OR attention* OR awareness* OR learning* OR "processing speed*" OR "executive function*" OR "visuo-spatial ability*" OR memory OR exergame* OR *virtual reality" OR VR OR (active NEAR/3 videogame*)))) AND (TITLE-ABS-KEY (((control* NEAR/3 (group* OR stud* OR trial*))) OR (random* OR assign* OR allocate* OR volunteer* OR crossover OR "cross-over" OR participant*)))	3'147 results (May 5, 2020)
Pedro	("heart rate variability" AND "short-term" AND exercis*) OR ("heart rate variability" AND "short-term" AND training*) OR ("heart rate variability" AND "short-term" AND learning) OR ("heart rate variability" AND "real-time")	35 results (May 5, 2020)
Pool		13,895 results (May 5, 2020)

3.6 Appendix C2: PRISMA-P 2015 Checklist

This checklist has been adapted for use with protocol submissions to Systematic Reviews from Table 3 in Moher D et al: Preferred reporting items for systematic review and meta-analysis protocols (PRISMA-P) 2015 statement. *Systematic Reviews* 2015 4:1 [84]

Section/topic	#	Checklist item	Information reported		Line number(s)
			Yes	No	
ADMINISTRATIVE INFORMATION					
Title					
Identification	1a	Identify the report as a protocol of a systematic review	☒	☐	1 - 4
Update	1b	If the protocol is for an update of a previous systematic review, identify as such	☐	☒	not applicable
Registration	2	If registered, provide the name of the registry (e.g., PROSPERO) and registration number in the Abstract	☒	☐	12 - 13
Authors					
Contact	3a	Provide name, institutional affiliation, and e-mail address of all protocol authors; provide physical mailing address of corresponding author	☒	☐	6, 9 - 11
Contributions	3b	Describe contributions of protocol authors and identify the guarantor of the review	☐	☒	
Amendments	4	If the protocol represents an amendment of a previously completed or published protocol, identify as such and list changes; otherwise, state plan for documenting important protocol amendments	☐	☒	not applicable
Support					
Sources	5a	Indicate sources of financial or other support for the review	☐	☒	not applicable
Sponsor	5b	Provide name for the review funder and/or sponsor	☐	☒	not applicable
Role of sponsor/funder	5c	Describe roles of funder(s), sponsor(s), and/or institution(s), if any, in developing the protocol	☐	☒	not applicable
INTRODUCTION					
Rationale	6	Describe the rationale for the review in the context of what is already known	☒	☐	14 - 78

frontiers

Section/topic	#	Checklist item	Information reported		Line number(s)
			Yes	No	
Objectives	7	Provide an explicit statement of the question(s) the review will address with reference to participants, interventions, comparators, and outcomes (PICO)	☒	☐	79 - 100
METHODS					
Eligibility criteria	8	Specify the study characteristics (e.g., PICO, study design, setting, time frame) and report characteristics (e.g., years considered, language, publication status) to be used as criteria for eligibility for the review	☒	☐	149 - 167
Information sources	9	Describe all intended information sources (e.g., electronic databases, contact with study authors, trial registers, or other grey literature sources) with planned dates of coverage	☒	☐	106 - 109
Search strategy	10	Present draft of search strategy to be used for at least one electronic database, including planned limits, such that it could be repeated	☒	☐	110 - 148
STUDY RECORDS					
Data management	11a	Describe the mechanism(s) that will be used to manage records and data throughout the review	☒	☐	169 - 175
Selection process	11b	State the process that will be used for selecting studies (e.g., two independent reviewers) through each phase of the review (i.e., screening, eligibility, and inclusion in meta-analysis)	☐	☐	169 - 175
Data collection process	11c	Describe planned method of extracting data from reports (e.g., piloting forms, done independently, in duplicate), any processes for obtaining and confirming data from investigators	☒	☐	177 - 180
Data items	12	List and define all variables for which data will be sought (e.g., PICO items, funding sources), any pre-planned data assumptions and simplifications	☒	☐	181 - 187
Outcomes and prioritization	13	List and define all outcomes for which data will be sought, including prioritization of main and additional outcomes, with rationale	☒	☐	186 - 191
Risk of bias in individual studies	14	Describe anticipated methods for assessing risk of bias of individual studies, including whether this will be done at the outcome or study level, or both; state how this information will be used in data synthesis	☒	☐	188 - 194
DATA					
Synthesis	15a	Describe criteria under which study data will be quantitatively synthesized	☒	☐	195 - 215

Section/topic	#	Checklist item	Information reported		Line number(s)
			Yes	No	
	15b	If data are appropriate for quantitative synthesis, describe planned summary measures, methods of handling data, and methods of combining data from studies, including any planned exploration of consistency (e.g., I^2, Kendall's tau)	☒	☐	195 - 215
	15c	Describe any proposed additional analyses (e.g., sensitivity or subgroup analyses, meta-regression)	☒	☐	195 - 215
	15d	If quantitative synthesis is not appropriate, describe the type of summary planned	☒	☐	195 - 215
Meta-bias(es)	16	Specify any planned assessment of meta-bias(es) (e.g., publication bias across studies, selective reporting within studies)	☒	☐	216 - 217
Confidence in cumulative evidence	17	Describe how the strength of the body of evidence will be assessed (e.g., GRADE)	☐	☒	
