# Supplementary material for: Can Reactivity of Heart Rate Variability Be a Potential Biomarker and Monitoring Tool to Promote Healthy Aging? A Systematic Review With Meta-Analyses
Source: Front Physiol. 2021 Jul 29;12:686129. doi: 10.3389/fphys.2021.686129 (PMC8359814; doi:10.3389/fphys.2021.686129)
Supplement: Supplementary file 3 [file Data_Sheet_2.PDF]

Supplementary File B - Screening Protocol

1 Screening on Title & Abstract

1.1 Preparation

1: Create Screening Tool AND 2: Hierarchy of Screening Tool

Guidelines: “Create an abstract screening tool with questions that are clear and concise. It should include items that (a) are objective, (b) are “single-barreled,” (c) use the same sentence structure, and (d) include yes/no/unsure answers only.” [1] AND “Ensure that the abstract screening tool is organized hierarchically, with the easiest questions at the beginning of the tool.” [1]

The abstract screening tool was developed based on the inclusion criteria of the study protocol. One screening question was defined for each PICOS criteria. These screening questions were organized hierarchically. During the screening process, eligibility will be assessed from top down. As soon as the first screening question is answered as a definite “no”, the record will be excluded. The remaining questions don’t need to be further assessed.

If all questions can be answered as a yes, or if eligibility remains unclear, records will be included on title & abstract. For all other records, the reasons for exclusion have to be provided. For this purpose, the following coding schema was prepared (table 1):

Table 1: Coding schema for Screening on Title & Abstract.

PICOS	Screening Question	Criteria	Eligibility	Coding
Study Report Characteristics:	(1) Does the study meet the defined study report characteristics?		Yes / unclear	continue with (2)
		○ Abstract is accessible	No	EXCLUDE on STUDY Report Characteristics: Abstract not accessible
		○ Written in English	No	EXCLUDE on STUDY Report Characteristics: Not written in English
		○ Published after 1996	No	EXCLUDE on STUDY Report Characteristics: Published before 1996
		○ Not a study protocol	No	EXCLUDE on STUDY Report Characteristics: Only Study Protocol
		○ Not a preliminary report	No	EXCLUDE on STUDY Report Characteristics: Only Preliminary Report
		○ Primary study publication	No	EXCLUDE on STUDY Report Characteristics: Secondary Publication of same study/database
**S**tudy Type:	(2) Was the study a:		Yes / unclear	continue with (3)
	○ Controlled Clinical Trial	e.g. randomized controlled trial, non-randomized controlled trial	No	EXCLUDE on STUDY TYPE: No Controlled Clinical Trial / Epidemiological Study
	○ Observational Study	e.g. cohort studies, cross-sectional studies, case-control studies		
**P**articipants:	(3) Did the study include human adults?		Yes / unclear	continue with (4)
		○ ≥ 18 years	No	EXCLUDE on PATIENT Characteristics: Not human adults

Table 1: Coding schema for Screening on Title & Abstract (continued)

PICOS	Screening Question	Criteria	Eligibility	Coding
**I**ntervention:	(4) Did the study apply any type of:		Yes / unclear	continue with (5)
	a) physical exercises	Physical exercises as defined by the American College of Sports Medicine (ACSM) [2] (e.g. cardiorespiratory exercise, resistance exercises, neuromotor exercise training)	No	EXCLUDE on INTERVENTION: No Physical and/or Cognitive Exercise Intervention
	b) cognitive exercise and/or tasks	cognitive exercise and/or tasks demanding motoric training related cognitive functions (e.g. attentional, executive and visuo-spatial functions, memory or processing speed)		
	c) simultaneous cognitive-motor training?	(as defined by [3])		
**O**utcomes:	(5) Did the study measure real-time HRV?	Measurement during the intervention	Yes / unclear	continue with (6)
			No	EXCLUDE on OUTCOME: Real-time HRV not assessed
**C**omparison:	(6) Did the study compare outcomes?	a) to resting state measurements?	Yes / unclear	Continue with (7)
			No	EXCLUDE on COMPARISON: No resting-state measurement of HRV
(7) Were all screening questions answered with yes / unclear?			Yes	INCLUDE on Title & Abstract

3: Pilot testing of Screening Tool

Guideline: "Conduct introductory abstract screening trainings where screeners learn and pilot test the tool by screening the same 20 to 30 abstracts. Repeat as necessary until team reaches consensus." [1]

The screening tool was pilot tested on the 20 most recent records on the database Pubmed including the terms "heart rate variability" AND "exercise". The following results were obtained:

Table 2: Pilot Screening of Screening Tool.

Reference	Coding		Agreement
	Reviewer 1 (PM)	Reviewer 2 (MT)	
Barrero et al. 2019 [4]	INCLUDE on Title & Abstract	INCLUDE on Title & Abstract	Yes
Berger et al. 2019 [5]	EXCLUDE on OUTCOME: Real-time HRV not measured	EXCLUDE on OUTCOME: Real-time HRV not measured	Yes
Boudet et al. 2017 [6]	EXCLUDE on OUTCOME: Real-time HRV not measured	EXCLUDE on OUTCOME: Real-time HRV not measured	Yes
Carpenter et al. 2017 [7]	INCLUDE on Title & Abstract	INCLUDE on Title & Abstract	Yes
Caruso et al. 2015 [8]	EXCLUDE on OUTCOME: Real-time HRV not measured	EXCLUDE on OUTCOME: Real-time HRV not measured	Yes
De Paula et al. 2019 [9]	EXCLUDE on OUTCOME: Real-time HRV not measured	EXCLUDE on OUTCOME: Real-time HRV not measured	Yes
Esco et al. 2017 [10]	EXCLUDE on OUTCOME: Real-time HRV not measured	EXCLUDE on OUTCOME: Real-time HRV not measured	Yes
Flogbé et al. 2018 [11]	EXCLUDE on OUTCOME: Real-time HRV not measured	EXCLUDE on OUTCOME: Real-time HRV not measured	Yes
Gonzaga et al. 2017 [12]	INCLUDE on Title & Abstract	INCLUDE on Title & Abstract	Yes
Hernando et al. 2018 [13]	INCLUDE on Title & Abstract	INCLUDE on Title & Abstract	Yes
Liu et al. 2018 [14]	EXCLUDE on OUTCOME: Real-time HRV not measured	EXCLUDE on OUTCOME: Real-time HRV not measured	Yes
Lu et al. 2016 [15]	INCLUDE on Title & Abstract	INCLUDE on Title & Abstract	Yes
Ludyga et al. 2019 [16]	INCLUDE on Title & Abstract	INCLUDE on Title & Abstract	Yes
Malfliet et al. 2018 [17]	INCLUDE on Title & Abstract	INCLUDE on Title & Abstract	Yes
Mankowski et al. 2017 [18]	INCLUDE on Title & Abstract	INCLUDE on Title & Abstract	Yes
Masroor et al. 2018 [19]	INCLUDE on Title & Abstract	INCLUDE on Title & Abstract	Yes
McNarry et al. 2019 [20]	EXCLUDE on PATIENT Characteristics:	EXCLUDE on PATIENT Characteristics:	Yes
Toni et al. 2016 [21]	INCLUDE on Title & Abstract	INCLUDE on Title & Abstract	Yes
Van der Zwan et al. 2015 [22]	EXCLUDE on INTERVENTION: No Physical and/or Cognitive Exercise Intervention	EXCLUDE on INTERVENTION: No Physical and/or Cognitive Exercise Intervention	Yes
Vesterinen et al. 2016 [23]	EXCLUDE on OUTCOME: Real-time HRV not measured	EXCLUDE on OUTCOME: Real-time HRV not measured	Yes

1.2 During the Screening Process:

4: Meeting of Screening Team

Guideline: "Meet with the abstract screening team on a weekly or every other week basis." [1]

The two independent reviewers will meet at least one a week to discuss the screening progress.

5: Modifications of Screening Tool

Guideline: "Minimize changes to the screening tool." [1]

The Screening Tool was modified to include an additional exclusion criterion:

- EXCLUDE on COMPARISON: No healthy control group

6: Text Mining

Guideline: "Use a text-mining abstract screening application." [1]

7: Double Screening Process

Guideline: "Require independent double-screening of each abstract." [1]

The screening and selection process will be pilot tested and executed by two independent reviewers (PM, MT). After completing screening on title & abstract, the retrieved results will be matched and discussed for final inclusion by (PM, MT).

8: Handling of disagreements

Guideline: „Reconcile disagreements throughout the abstract screening process." [1]

In case of disagreement, (EdB) will serve as referee.

9: Encouragement of Screening Process

Guideline: "Encourage screening through intellectual buy-in and incentives." [1]

1.3 After the Screening Process:

Analyze Screening Procedure

Guideline: "Analyze the process and decisions after screening has been completed." [1]

The inter-rater agreement was high, and a final decision could be made for all records.

2 Screening on Full Text

For screening on full text, all eligibility criteria were assessed in detail. The following coding schema was prepared (table 2):

Table 3: Coding schema for Screening on Full Text

PICOS	Screening Question	Criteria	Eligibility	Coding
Study Report Characteristics:	(1) Does the study meet the defined study report characteristics?		Yes / unclear	continue with (2)
		○ Full text is accessible	No	EXCLUDE on STUDY Report Characteristics: Full text not accessible
		○ Written in English	No	EXCLUDE on STUDY Report Characteristics: Not written in English
		○ Not a conference abstract/poster/letter	No	EXCLUDE on STUDY Report Characteristics: Only Conference Abstract / Poster
		○ Not a study protocol	No	EXCLUDE on STUDY Report Characteristics: Only Study Protocol
		○ Not a preliminary report	No	EXCLUDE on STUDY Report Characteristics: Only Preliminary Report
		○ Primary study publication	No	EXCLUDE on STUDY Report Characteristics: Secondary Publication of same study/database
**P**articipants:	(2) Did the study include human adults?		Yes / unclear	continue with (3)
		○ ≥ 18 years	No	EXCLUDE on PARTICIPANT Characteristics: Not human adults
**S**tudy Type:	(3) Was the study a:		Yes / unclear	continue with (4)
	○ Controlled Clinical Trial	e.g. randomized controlled trial, non-randomized controlled trial	No	EXCLUDE on STUDY TYPE: No Controlled Clinical Trial / Epidemiological Study
	○ Observational Study	e.g. cohort studies, cross-sectional studies, case-control studies		
**O**utcomes:	(4) Did the study measure real-time HRV?		Yes / unclear	continue with (5)
		○ Measurement during the intervention	No	EXCLUDE on OUTCOME: Real-time HRV not assessed
		○ Reporting of numeric data	No	EXCLUDE on OUTCOME: No numeric data reported

Table 3: Coding schema for Screening on Full Text (continued)

PICOS	Screening Question	Criteria	Eligibility	Coding
**C**omparison:	(5) Did the study compare outcomes?		Yes / unclear	Continue with (6)
		○ to resting state measurements?	No	EXCLUDE on COMPARISON:<br>No resting-state measurement of HRV
		○ In case of pathological participants: to a healthy control group	No	EXCLUDE on COMPARISON:<br>No healthy control group
**O**utcomes:	(6) Did the study measure real-time HRV?		Yes / unclear	Continue with (7)
		• Measurements according to the standards of measurement defined by [24]	No	EXCLUDE on OUTCOME:<br>HRV measurement does not meet the standards of measurement
		○ Measurements with a validated device based on electrocardiography, photo-plethysmography or pulsoxymetry	No	EXCLUDE on OUTCOME:<br>Measurement device not validated
**I**ntervention:	(7) Did the study apply any type of:		Yes / unclear	continue with (8)
	a) physical exercises	Physical exercises as defined by the American College of Sports Medicine (ACSM) [2] (e.g. cardiorespiratory exercise, resistance exercises, neuromotor exercise training)	No	EXCLUDE on INTERVENTION:<br>No Physical and/or Cognitive Exercise Intervention
	b) cognitive exercise	cognitive exercises demanding motoric training related cognitive functions (e.g. attentional, executive and visuo-spatial functions, memory or processing speed)		
	c) simultaneous cognitive-motor training?	(as defined by [3])		
(8) Were all screening questions answered with yes / unclear?			Yes	INCLUDE on Full Text

3 References

1. Polanin, J.R., et al., Best practice guidelines for abstract screening large-evidence systematic reviews and meta-analyses. Research Synthesis Methods, 2019. **10**(3): p. 330-342.
2. Garber, C.E., et al., American College of Sports Medicine position stand. Quantity and quality of exercise for developing and maintaining cardiorespiratory, musculoskeletal, and neuromotor fitness in apparently healthy adults: guidance for prescribing exercise. Med Sci Sports Exerc, 2011. **43**(7): p. 1334-59.
3. Herold, F., et al., Thinking While Moving or Moving While Thinking – Concepts of Motor-Cognitive Training for Cognitive Performance Enhancement. Frontiers in Aging Neuroscience, 2018. **10**(228).
4. Barrero, A., et al., Daily fatigue-recovery balance monitoring with heart rate variability in well-trained female cyclists on the Tour de France circuit. PLoS One, 2019. **14**(3): p. e0213472.
5. Berger, M., et al., Effect of exercise training on heart rate variability in patients with obstructive sleep apnea: A randomized controlled trial. Scand J Med Sci Sports, 2019. **29**(8): p. 1254-1262.
6. Boudet, G., et al., Paradoxical dissociation between heart rate and heart rate variability following different modalities of exercise in individuals with metabolic syndrome: The RESOLVE study. Eur J Prev Cardiol, 2017. **24**(3): p. 281-296.
7. Carpenter, R.E., et al., Influence of antenatal physical exercise on heart rate variability and QT variability. J Matern Fetal Neonatal Med, 2017. **30**(1): p. 79-84.
8. Caruso, F.R., et al., Resistance exercise training improves heart rate variability and muscle performance: a randomized controlled trial in coronary artery disease patients. Eur J Phys Rehabil Med, 2015. **51**(3): p. 281-9.
9. de Paula, T., et al., Acute Effect of Aerobic and Strength Exercise on Heart Rate Variability and Baroreflex Sensitivity in Men With Autonomic Dysfunction. J Strength Cond Res, 2019. **33**(10): p. 2743-2752.
10. Esco, M.R., A.A. Flatt, and H.N. Williford, Postexercise heart rate variability following treadmill and cycle exercise: a comparison study. Clin Physiol Funct Imaging, 2017. **37**(3): p. 322-327.
11. Fiogbé, E., et al., Water exercise in coronary artery disease patients, effects on heart rate variability, and body composition: A randomized controlled trial. Physiother Res Int, 2018. **23**(3): p. e1713.
12. Gonzaga, L.A., et al., Caffeine affects autonomic control of heart rate and blood pressure recovery after aerobic exercise in young adults: a crossover study. Sci Rep, 2017. **7**(1): p. 14091.
13. Hernando, D., et al., Methodological framework for heart rate variability analysis during exercise: application to running and cycling stress testing. Med Biol Eng Comput, 2018. **56**(5): p. 781-794.
14. Liu, J., et al., The Effects of Tai Chi on Heart Rate Variability in Older Chinese Individuals with Depression. Int J Environ Res Public Health, 2018. **15**(12).
15. Lu, D.Y., et al., Heart Rate Variability Is Associated with Exercise Capacity in Patients with Cardiac Syndrome X. PLoS One, 2016. **11**(1): p. e0144935.
16. Ludyga, S., et al., Immediate and sustained effects of intermittent exercise on inhibitory control and task-related heart rate variability in adolescents. J Sci Med Sport, 2019. **22**(1): p. 96-100.
17. Malfliet, A., et al., Cerebral Blood Flow and Heart Rate Variability in Chronic Fatigue Syndrome: A Randomized Cross-Over Study. Pain Physician, 2018. **21**(1): p. E13-e24.
18. Mankowski, R.T., et al., Heart-Rate Variability Threshold as an Alternative for Spiro-Ergometry Testing: A Validation Study. J Strength Cond Res, 2017. **31**(2): p. 474-479.

19. Masroor, S., et al., Heart Rate Variability following Combined Aerobic and Resistance Training in Sedentary Hypertensive Women: A Randomised Control Trial. Indian Heart J, 2018. **70 Suppl 3**(Suppl 3): p. S28-s35.

20. McNarry, M.A., et al., Effect of asthma and six-months high-intensity interval training on heart rate variability during exercise in adolescents. J Sports Sci, 2019. **37**(19): p. 2228-2235.

21. Toni, G., et al., Physical Exercise for Late-Life Depression: Effects on Heart Rate Variability. Am J Geriatr Psychiatry, 2016. **24**(11): p. 989-997.

22. van der Zwan, J.E., et al., Physical activity, mindfulness meditation, or heart rate variability biofeedback for stress reduction: a randomized controlled trial. Appl Psychophysiol Biofeedback, 2015. **40**(4): p. 257-68.

23. Vesterinen, V., et al., Individual Endurance Training Prescription with Heart Rate Variability. Med Sci Sports Exerc, 2016. **48**(7): p. 1347-54.

24. Malik, M., Heart rate variability: Standards of measurement, physiological interpretation, and clinical use. Circulation, 1996. **93**: p. 1043-1065.

25. Steptoe, A., et al., Stress responsivity and socioeconomic status: a mechanism for increased cardiovascular disease risk? European heart journal, 2002. **23**(22): p. 1757-1763.
